# Convergent molecular evolution of phosphoenolpyruvate carboxylase gene family in C_4_ and crassulacean acid metabolism plants

**DOI:** 10.7717/peerj.12828

**Published:** 2022-01-20

**Authors:** Jiang-Ping Shu, Yue-Hong Yan, Rui-Jiang Wang

**Affiliations:** 1 Key laboratory of Plant Resources Conservation and Sustainable Utilization, South China Botanical Garden, Chinese Academy of Sciences, Guangzhou, China; 2 Key Laboratory of National Forestry and Grassland Administration for Orchid Conservation and Utilization, The National Orchid Conservation Centre of China and The Orchid Conservation and Research Centre of Shenzhen, Shenzhen, China; 3 Shenzhen Key Laboratory for Orchid Conservation and Utilization, The National Orchid Conservation Centre of China and The Orchid Conservation and Research Centre of Shenzhen, Shenzhen, China; 4 University of Chinese Academy of Sciences, Beijing, China

**Keywords:** PEPC, Convergent evolution, Carbon-concentrating mechanisms, Photosynthetic convergence, Phylogeny

## Abstract

Phosphoenolpyruvate carboxylase (PEPC), as the key enzyme in initial carbon fixation of C_4_and crassulacean acid mechanism (CAM) pathways, was thought to undergo convergent adaptive changes resulting in the convergent evolution of C_4_ and CAM photosynthesis in vascular plants. However, the integral evolutionary history and convergence of PEPC in plants remain poorly understood. In the present study, we identified the members of PEPC gene family across green plants with seventeen genomic datasets, found ten conserved motifs and modeled three-dimensional protein structures of 90 plant-type PEPC genes. After reconstructing PEPC gene family tree and reconciled with species tree, we found PEPC genes underwent 71 gene duplication events and 16 gene loss events, which might result from whole-genome duplication events in plants. Based on the phylogenetic tree of the PEPC gene family, we detected four convergent evolution sites of PEPC in C_4_ species but none in CAM species. The PEPC gene family was ubiquitous and highly conservative in green plants. After originating from gene duplication of ancestral C3-PEPC, C4-PEPC isoforms underwent convergent molecular substitution that might facilitate the convergent evolution of C_4_ photosynthesis in Angiosperms. However, there was no evidence for convergent molecular evolution of PEPC genes between CAM plants. Our findings help to understand the origin and convergent evolution of C_4_ and CAM plants and shed light on the adaptation of plants in dry, hot environments.

## Introduction

Plant photosynthesis is the most important biochemical process on the planet: it provides energy, food and oxygen for the survival and reproduction of vast majority though heterotrophic organisms, including humans (Fischer, Hemp & Johnson, 2016). Since its origin, oxygen levels have gradually risen, and carbon dioxide (CO_2_) concentrations have decreased in the atmosphere (Foster, Royer & Lunt, 2017), which has increased photorespiration and led to bioenergy and carbohydrate losses (Schlüter & Weber, 2020). To obtain adequate CO_2_ and improve photosynthetic efficiency, plants developed several carbon-concentrating mechanisms (CCMs), such as C_4_ photosynthesis and crassulacean acid metabolism (CAM), so it could adapt to the sudden decline of atmospheric CO_2_ approximately 350 million years ago (Mya) (Edwards, 2019; Heyduk et al., 2019). C_4_ photosynthesis, an example of convergent evolution, has repeatedly evolved more than 60 times in angiosperms (Sage, Christin & Edwards, 2011; Sage, 2016); whereas, CAM has independently evolved in approximately 37 families of vascular plants (Silvera et al., 2010; Winter et al., 2021). Recently, several studies using comparative genomics have provided new insights into the genetics and evolution of CCMs (Yang et al., 2017; Heyduk et al., 2019; Wai et al., 2019; Yang et al., 2019; Jaiswal et al., 2021). However, the molecular mechanisms underlying convergent evolution in CCMs remain poorly understood.

In plants, phosphoenolpyruvate carboxylase (PEPC; EC 4.1.1.31) is a key enzyme that catalyzes the primary fixation reaction for CO_2_ assimilation in C_4_ and CAM photosynthesis (Lepiniec et al., 1994; Nimmo, 2000; Svensson, Bläsing & Westhoff, 2003). PEPC is a highly regulated enzyme that catalyzes the irreversible *β*-carboxylation of phosphoenolpyruvate in the presence of bicarbonate and magnesium to yield oxaloacetate and inorganic phosphate, a reaction that serves a variety of physiological functions in plants (Jiao & Chollet, 1991; Lepiniec et al., 1994; Chollet, Vidal & O’Leary, 1996). Additionally, PEPC genes play crucial roles in a variety of non-photosynthetic functions, including carbon and nitrogen metabolism, seed development and germination and response to abiotic stresses (Lepiniec et al., 1994; O’Leary, Park & Plaxton William, 2011; Shi et al., 2015; Ruiz-Ballesta et al., 2016; Wang et al., 2016; Darabi & Seddigh, 2018; Zhao et al., 2019). As housekeeping genes, PEPC genes are highly conserved, which makes them valuable molecular markers for phylogenetic reconstruction of plants (Gehrig, Heute & Kluge, 2001). Although PEPC genes share origins during the evolution of C_4_ and CAM photosynthesis, these genes are distinct copies that were generated by whole-genome duplication (WGD) in angiosperms (Christin et al., 2014).

Interestingly, PEPC genes are essential for regulation of the circadian clock in CAM photosynthesis (Boxall et al., 2020) and share convergent amino acid changes in diverse CAM species (Yang et al., 2017). In C_4_ grasses, PEPC genes have also undergone parallel, adaptive genetic changes (Christin et al., 2007; Besnard et al., 2009; Moreno-Villena et al., 2018). Therefore, PEPC genes are crucial for elucidating the origin and convergent evolution of C_4_ and CAM photosynthesis. Previous studies only examined the convergent evolution of PEPC gene family in a few C_4_or CAM photosynthetic lineages, such as grass (Christin et al., 2007; Besnard et al., 2009; Moreno-Villena et al., 2018) and angiosperms (Yang et al., 2017), but without *Isoetes*, which was the earliest-diverging lineage of CAM plants (Keeley, 1981), and without fern CAM plants such as *Platycerium* (Rut et al., 2008), so the origin and evolution of the PEPC gene family could not be clearly elucidated (Deng et al., 2016). Furthermore, CAM photosynthesis occurs widely in the major clades of vascular plants, including pteridophytes (lycopods and ferns), gymnosperms and angiosperms, while C_4_ photosynthesis is only distributed in angiosperms. Therefore, to understand the convergent molecular evolution of the PEPC gene family in the plant kingdom, species with genomic datasets that represent C_3_, C_4_ and CAM photosynthesis across the major lineages of plants should be sampled.

Fortunately, more and more plant genomes have been sequenced (https://www.plabipd.de/index.ep), which provides an excellent opportunity to resolve the origin and convergent evolution of C_4_ and CAM photosynthesis in plants (Heyduk et al., 2019; Yang et al., 2019; Gilman & Edwards, 2020). However, it is a big challenge to use hundreds of genomic datasets to analyze the convergent evolution of PEPC genes, which have multiple copies in most species. In the present study, we only selected 17 plant genomes, which consisted of C_3_, C_4_ and CAM species across algae, bryophytes, pteridophytes, gymnosperms and angiosperms, especially included *Isoetes* and *Platycerium*, which represented the earliest-diverging lineages of CAM plants. Then we identified the PEPC genes from the 17 genomes and reconstructed the evolutionary history and molecular convergence of PEPC genes in C_4_ and CAM plants. Our study will help to elucidate the origin and evolution of C_4_ and CAM plants and shed light on the adaptation of plants in dry, hot environments.

## Materials & Methods

### Data acquisition

C_4_ and CAM photosynthesis are widely distributed in angiosperms and vascular plants, respectively (Heyduk et al., 2019). To obtain representative sampling of these groups and avoid the difficulties in analysis with big datasets, we only selected 17 plant species that exhibited a variety of photosynthetic pathways (including C_3_, C_4_ and CAM): *Spirogloea muscicola*, which is the closest algae to land plants (Cheng et al., 2019), three bryophytes, two lycopods, two ferns, two gymnosperms and seven angiosperms (Table 1). Seventeen genomic datasets were obtained from public databases, such as Figshare (https://figshare.com/), EnsemblPlants (http://plants.ensembl.org), Dryad (https://datadryad.org), Fernbase (https://www.fernbase.org/), GigaDB (http://gigadb.org/), Congenie (https://congenie.org/), and Phytozome (v13, https://phytozome-next.jgi.doe.gov).

**Table 1:** The information of samples used in this study.

ID	Species	Class	Type	PTPC	BTPC	PEPCase superfamily	Data resources
Spm	*Spirogloea muscicola*	Algae	C3	3	2	4	Figshare
Mp	*Marchantia polymorpha*	Bryophytes	C3	1	1	1	EnsemblPlants
Pp	*Physcomitrella patens*	Bryophytes	C3	22	0	10	EnsemblPlants
Aa	*Anthoceros angustus*	Bryophytes	C3	1	1	2	Dryad
Sm	*Selaginella moellendorffii*	Lycopods	C3	4	4	0	EnsemblPlants
Is	*Isoetes sinensis*	Lycopods	CAM	2	5	0	This study
Af	*Azolla filiculoides*	Ferns	C3	2	0	3	Fernbase
Pb	*Platycerium bifurcatum*	Ferns	CAM	2	0	2	GigaDB
Pa	*Picea abies*	Gymnosperms	C3	0	0	10	Congenie
Gm	*Gnetum montanum*	Gymnosperms	C3	1	1	0	Dryad
Amt	*Amborella trichopoda*	Angiosperms	C3	1	1	0	EnsemblPlants
Ac	*Ananas comosus*	Angiosperms	CAM	2	0	1	EnsemblPlants
Os	*Oryza sativa*	Angiosperms	C3	9	1	4	EnsemblPlants
Zm	*Zea mays*	Angiosperms	C4	22	1	33	EnsemblPlants
At	*Arabidopsis thaliana*	Angiosperms	C3	8	1	0	EnsemblPlants
Ah	*Amaranthus hypochondriacus*	Angiosperms	C4	3	0	0	Phytozome
Kf	*Kalanchoe fedtschenkoi*	Angiosperms	CAM	8	1	0	Phytozome

**Notes.**

PTPC plant-type PEPC BTPC bacterial-type PEPC

### Gene family identification

To identify members of the PEPC gene family, we created a local BLAST database with protein sequences from the 17 plant species and then performed BLASTP searches with default parameters using PEPC protein sequences from Arabidopsis (At1g53310, At1g68750, At2g42600 and At3g14940) as queries (Camacho et al., 2009). Furthermore, the Pfam seed alignment of the PEPcase domain (PF00311, http://pfam.xfam.org/) was used to build the HMMER profile; then, we searched for candidate PEPC genes in the 17 genomic datasets using HMMER v3.2.1 (Mistry et al., 2013). After combining the two PEPC gene sets, we searched for conserved domains from the Conserved Domain Database using Batch CD-Search with default parameters (Lu et al., 2020). Only genes with the PEPcase conserved domain were identified as reliable plant-type PEPC genes, and bacterial-type PEPC gene contained the PRK00009 domain.

### Prediction of conserved motifs and modeling of protein structure

After identifying reliable PEPC genes with our pipeline above, conserved motifs were predicted by MEME v5.1.0 (Bailey et al., 2006) with default parameters. Motif alignment was performed by MAST v5.1.0 with default parameters, and conserved motifs were visualized by TBtools v1.046 (Chen et al., 2020a). The 3D structure homology modelling of PEPC proteins was predicted by SWISS-MODEL (Waterhouse et al., 2018), which integrate up-to-date protein sequence and structure database as the structural templates. We selected the optimal protein model with the highest values of Global Model Quality Estimate (GMQE) and sequence identify, which indicated the highest reliability of the homology modelling. To detected the convergence of 3D protein structure, we simply test that if there is a 3D structure that only exists in CAM or C_4_ plants, we think this structure is convergent.

### Phylogenetic reconstruction and gene tree-species tree reconciliation

To understand the evolution of the PEPC gene family, the alignment of PEPC protein sequences was performed by MAFFT v7.453 (Katoh & Standley, 2013) with the accurate L-INS-i method and 1000 maximum iterative refinements. The conserved blocks were selected by GBLOCKS 0.91b (Castresana, 2000) with the parameters that minimum length of a block was five and allowed gap positions with half. Then, we reconstructed the PEPC gene family tree with maximum likelihood using IQ-TREE v1.6.11 (Nguyen et al., 2015). The best-fit amino acid model, JTTDCMut+R5, was detected by ModelFinder (Kalyaanamoorthy et al., 2017) using the Bayesian information criterion. The ultrafast bootstrap approximation was calculated using 1000 random replicates (Hoang et al., 2017). Phylogenetic reconciliation of the gene tree and species tree was performed by Treerecs (Comte et al., 2020) with default parameters. The species tree used was based on recent phylogenomic reconstruction of green plants (Initiative, 2019).

### Convergent site detection

The convergent site definition of Rey et al. (2018), that “a substitution is convergent if it occurred toward the same amino acid preference on every branch where the phenotype also changed toward the convergent phenotype”, were employed in this study, because several amino acids with similar biochemical properties may have roughly the same fitness at that site (Rey et al., 2018), it indicated that convergent site may be not the exact same amino acid in all species with a convergent phenotype. In addition, only some of PEPC gene copies are possibly involved in CAM or C_4_ and others are not, but it is difficult to identify which one is the isoform of CAM or C_4_. Therefore, we labeled putative convergent clades or gene copies on the phylogenetic tree with three kinds of gene combinations: (1) including all gene copies of species with convergent phenotypes (C_4_ or CAM), (2) only containing one clade within each convergent species and (3) only containing one gene copy within each convergent species. For each combination, convergent amino acid site of PEPC proteins in C_4_ and CAM plants was detected based on the PCOC model with a posterior probability threshold of 0.8 (Rey et al., 2018).

## Results

### Identification of PEPC gene family

As the key carboxylase, PEPC genes are widely distributed in green plants (Table 1). In the present study, we identified 264 homologous genes using BLASTP searching with Arabidopsis PEPC genes (At1g53310, At1g68750, At2g42600 and At3g14940) as queries and 179 homologous genes using the Pfam seed alignment of the PEPcase domain (PF00311) from 17 genomic datasets across green plants. After combination of the two gene sets, we obtained 179 common, homologous genes and then searched for conserved domains using the Conserved Domain Database: 109 genes contained conserved domains, of which 90 contained the plant-type PEPC (PTPC) gene domain (PEPcase) and 19 contained the bacterial-type PEPC (BTPC) gene domain (PRK00009); the remaining 70 genes contained other PEPcase superfamily domains (Tables 1, S1).

**Table 2:** Conserved motifs of PEPC gene family in green plants.

Motifs	Width	Sites	LLR	*E*-value	Consensus sequence
1	29	109	8856	4.7e−3069	RKPSGGIESL**R**AIPWIF**A**WTQTRFHLPVW
2	50	109	14268	9.6e−5072	[VI]KLTMFHGRGG[TS]VG**R**GGGPTHLAILSQPPDT[IV][HN]GSLRVT[VI]QGEVIEQSFG
3	44	108	12398	9.6e−4364	SS**W**MGGDRDGNPRVTPEVTRDVCLLAR[ML]MAANLYFSQIEDLMFE
4	50	104	13093	2.6e−4568	IQAA[FW]RTDEIRRT[PQ]PTPQDEMRAG[ML]SYFHETIW[KN]G[VL]PKFLRRVDTALKNI
5	37	105	10252	2.1e−3574	FSI[DE]WY[RL]NRINGKQEV**M**IGYS**D**SG**K**DAGR[LF]SAAWQ[LM][YF]
6	41	109	10936	4.4e−3771	DVL[DG]TF[HRK]V[IL]AELP[SA]D[SC]FGAY[IV]ISMATAPSDVLAVELLQREC
7	41	109	10861	2.7e−3739	DF[LM]RQVS[TC]FGLSLV[KR]LDIRQES[DE]RHT[DE]V[LM]DAIT[TN][HY]LGIGSY
8	50	109	12887	1.6e−4467	WRAL[ML]DE[MI]AVV[AS]T[KE]EYRS[IV]VF[QK][EN]PRFVEYFRLATPELEYGR[ML]NIGS**R**PSK
9	41	108	10512	6.4e−3599	[TM]L[QR][EA]MYN[EQ]WPFFRVTIDL[VI]EMVFA**K**GDP[GR]IAALYDKLLVS[ED]
10	29	108	7470	7.5e−2482	EEHLCFRTLQR[FY]TAATLEHGMHPPI[SA]PKP

**Notes.**

Width the length of motif Sites the number of sequences including the motif LLR log likelihood rate

Amino acid residues of experimentally proven function in Wang et al. (2016) are indicated by red majuscules.

### Conserved motifs and structures of PEPC gene family

**Figure 1:**
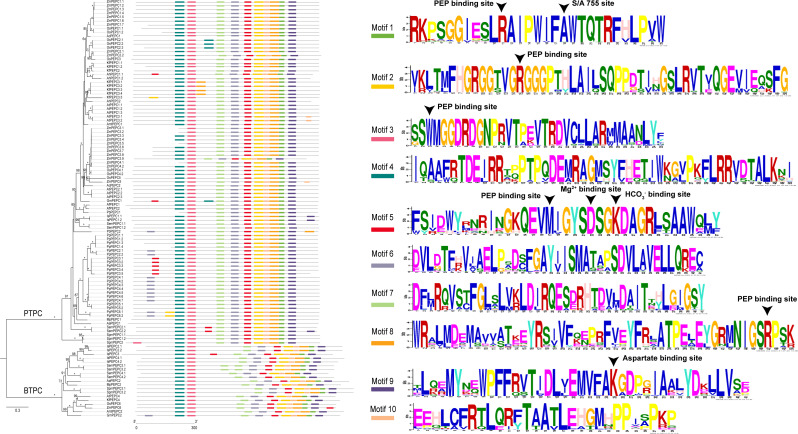
The phylogenetic tree and ten conserved motifs of PEPC gene family in plants. The asterisk in the maximum likelihood tree indicated 100 bootstrap support value, and the value lower than 60 was not showed in gene family tree. Arrowhead is indicated the amino acid residues of experimentally proven function in Wang et al. (2016). PTPC: plant-type PEPC; BTPC: bacterial-type PEPC. Aa, *Anthoceros angustus*; Ac, *Ananas comosus*; Af: *Azolla filiculoides*; Ah, *Amaranthus hypochondriacus*; Amt, *Amborella trichopoda*; At, *Arabidopsis thaliana*; Gm, *Gnetum montanum*; Is, *Isoetes sinensis*; Kf, *Kalanchoe fedtschenkoi*; Mp, *Marchantia polymorpha*; Os, *Oryza sativa*; Pa, *Picea abies*; Pb, *Platycerium bifurcatum*; Pp, *Physcomitrella patens*; Sm, *Selaginella moellendorffii*; Spm, *Spirogloea muscicola*; Zm, *Zea mays*.

**Figure 2:**
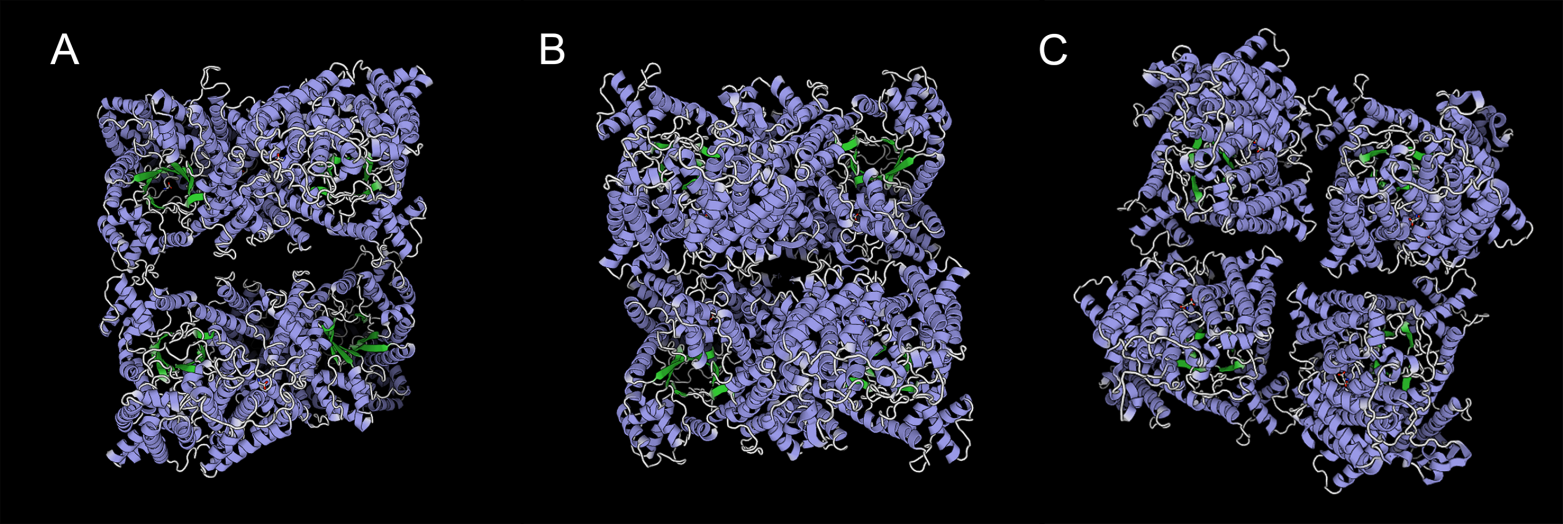
Three templates of three-dimensional structure in PTPC proteins,. (A) 5vyj.1.A; (B) 3zgb.1.A; (C) 5fdn.1.A. The three pictures are viewed from the same angles.

**Figure 3:**
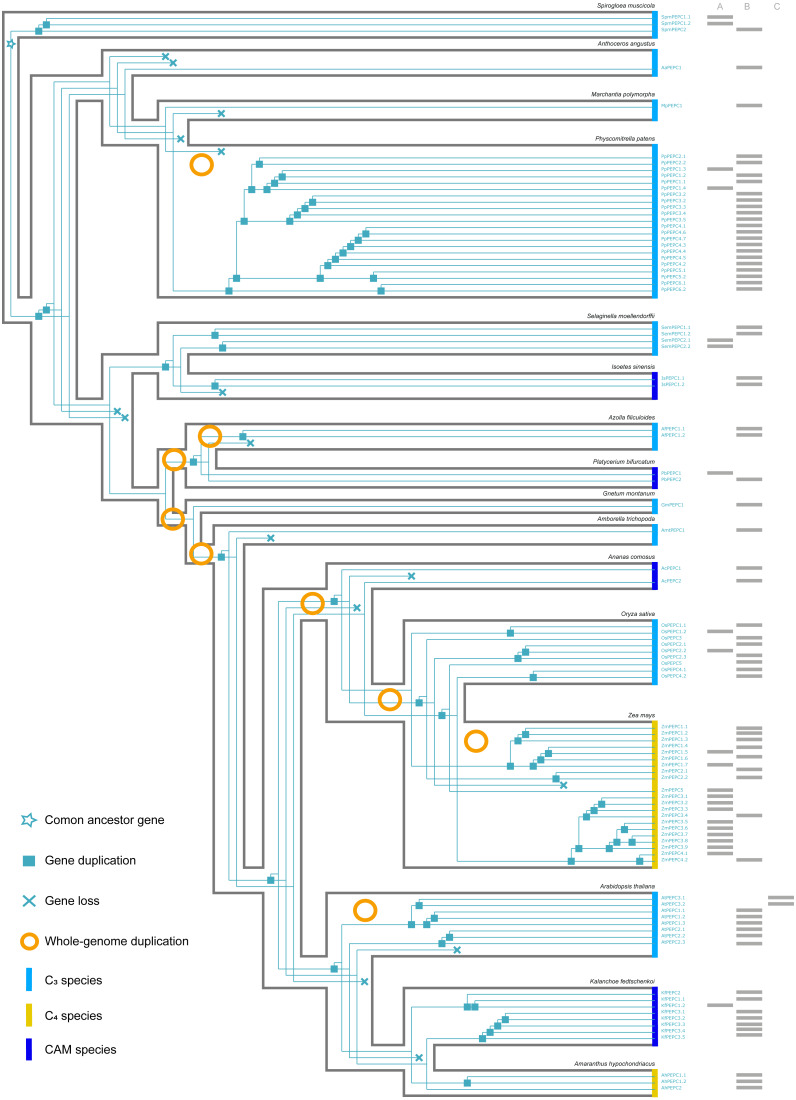
The evolutionary history of PTPC gene family in green plants. (A, B, C) respectively corresponded to three protein structures (A, B, C) in Fig. 2. The information of whole-genome duplication events was based on previous studies (Cheng et al., 2018; Li et al., 2018). Aa: *Anthoceros angustus*; Ac, *Ananas comosus*; Af, *Azolla filiculoides*; Ah, *Amaranthus hypochondriacus*; Amt, *Amborella trichopoda*; At, *Arabidopsis thaliana*; Gm, *Gnetum montanum*; Is, *Isoetes sinensis*; Kf, *Kalanchoe fedtschenkoi*; Mp, *Marchantia polymorpha*; Os, *Oryza sativa*; Pa, *Picea abies*; Pb, *Platycerium bifurcatum*; Pp, *Physcomitrella patens*; Sm, *Selaginella moellendorffii*; Spm, *Spirogloea muscicola*; Zm, *Zea mays*.

We predicted ten conserved motifs from 109 PEPC proteins. Each motif was longer than 29 amino acids and was found in more than 104 of the 109 PEPC proteins (Table 2). Most of the amino acids were conserved across all motifs, and the linear order of these motifs, especially in PTPC genes, was identical across all green plants. Some motifs were repeated in various genes (Fig. 1). In order to test if the protein structures evolved convergent in CAM or C_4_ plants, we modeled 3D structures of 90 PTPC proteins using the SWISS-MODEL server (Fig. S1) and obtained three templates of protein structures (Table S2): 5vyj.1.A (Fig. 2A), 3zgb.1.A (Fig. 2B) and 5fdn.1.A (Fig. 2C). The 3zgb.1.A template was widely distributed in all sampled species. The 5vyj.1.A template was distributed in seven species. The 5fdn.1.A template was only distributed in Arabidopsis (Fig. 3, Table S2).

### Gene duplication and loss

Here, we reconstructed the robust phylogenetic tree of the PEPC gene family with relatively adequate sampling to include C_3_, C_4_ and CAM plants and the bootstrap support values of all branches were mostly higher than 60 (Fig. 1). And the evolutionary history of PTPC genes was independently reconstructed with maximum likelihood and was reconciled with the species tree based on duplication-loss reconciliation (Fig. 3). The reconciliation tree showed that PTPC genes underwent at least 71 duplications and 16 losses in the evolutionary history of our sampled species.

### Convergent evolution of PEPC gene family

**Figure 4:**
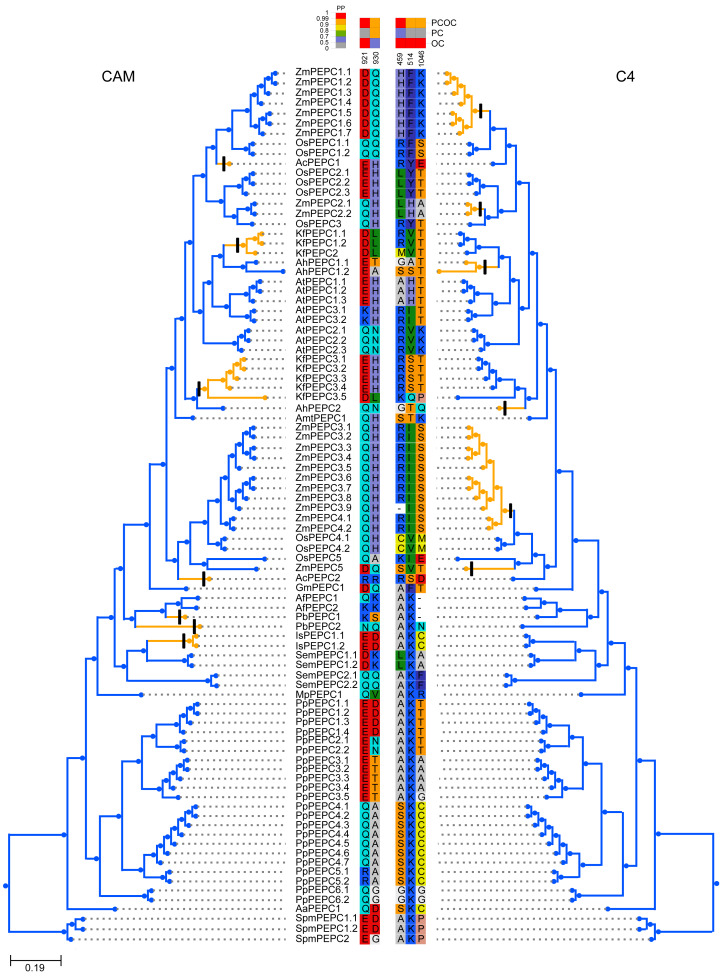
Molecular convergent sites of PEPC gene family in CAM and C_4_ species. The clades with orange color indicated the species with convergent phenotypes (CAM and C_4_ photosynthesis). PCOC: Profile Change with One Change model; PC: Profile Change model; OC: One Change model, all models were in detail explained by Rey et al. (2018). Posterior probabilities (pp) for the PCOC, PC, and OC models are summarized by top box colors, and the amino acid colors correspond to different amino acid equilibrium frequencies (*i.e.*, different profiles) of the Profile Change with One Change model (PCOC model). Aa, *Anthoceros angustus*; Ac, *Ananas comosus*; Af, *Azolla filiculoides*; Ah, *Amaranthus hypochondriacus*; Amt: *Amborella trichopoda*; At, *Arabidopsis thaliana*; Gm, *Gnetum montanum*; Is, *Isoetes sinensis*; Kf, *Kalanchoe fedtschenkoi*; Mp, *Marchantia polymorpha*; Os, *Oryza sativa*; Pa, *Picea abies*; Pb, *Platycerium bifurcatum*; Pp, *Physcomitrella patens*; Sm, *Selaginella moellendorffii*; Spm, *Spirogloea muscicola*; Zm, *Zea mays*.

**Figure 5:**
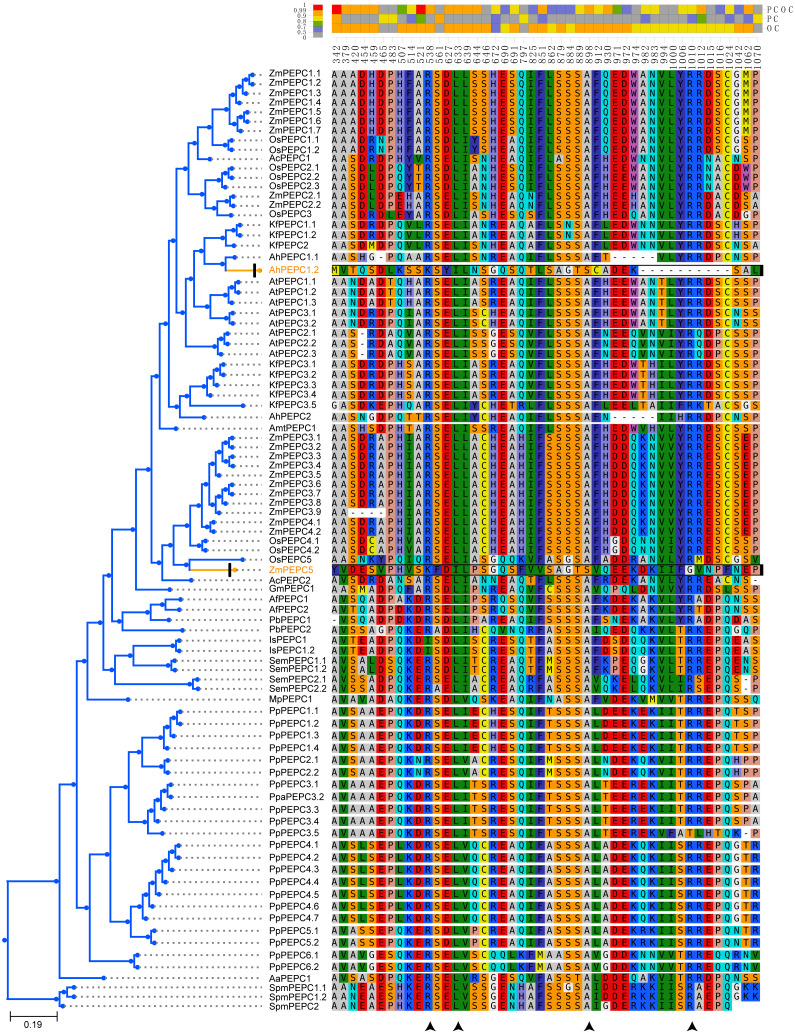
Four convergent substitution sites of C4-PEPC isoforms. The arrowheads at the bottom showed the positions of convergent substitutions. PCOC: Profile Change with One Change model; PC, Profile Change model; OC, One Change model, all models were in detail explained by Rey et al. (2018). Posterior probabilities (pp) for the PCOC, PC, and OC models are summarized by top box colors, and the amino acid colors correspond to different amino acid equilibrium frequencies (*i.e.*, different profiles) of the Profile Change with One Change model (PCOC model). Aa, *Anthoceros angustus*; Ac, *Ananas comosus*; Af, *Azolla filiculoides*; Ah, *Amaranthus hypochondriacus*; Amt, *Amborella trichopoda*; At, *Arabidopsis thaliana*; Gm, *Gnetum montanum*; Is, *Isoetes sinensis*; Kf, *Kalanchoe fedtschenkoi*; Mp, *Marchantia polymorpha*; Os: *Oryza sativa*; Pa, *Picea abies*; Pb, *Platycerium bifurcatum*; Pp, *Physcomitrella patens*; Sm, *Selaginella moellendorffii*; Spm, *Spirogloea muscicola*; Zm, *Zea mays*.

To test whether convergent molecular evolution at the amino acid level occurred in PTPC proteins, we detected convergent sites in all phenotypically convergent clades of C_4_ and CAM photosynthesis with the Profile Change with One Change (PCOC) pipeline. The results showed two convergent shifts in PTPC proteins that occurred in CAM species and three convergent shifts that occurred in C_4_ species, the posterior probabilities (pp) for the PCOC model were greater than 0.9 at all convergent shifts (Fig. 4). In addition, we also detected convergent evolution at sites in different gene groups, in which each convergent phenotypic species retained one clade or one gene copy (one-to-one) (Fig. S2). Four identical convergent amino acid sites in one gene group (AhPEPC1.2/ZmPEPC2) were discovered in C_4_ species (Fig. 5). However, no identical convergent sites were found in the one-to-one gene groups of CAM species (Fig. S2).

## Discussions

### PEPC gene family with different copies was conserved in plants

PEPC gene family was ubiquitous but with different copy number in the different lineages of green plants. In the present study, BTPC genes were distributed in 11 of the 17 sampled species and retained relatively fewer copies than PTPC genes (Table 1). Because of missing the conserved PEPcase domain, we did not detect any PTPC genes in Norway spruce (*Picea abies*). This might be resulted from pseudogenization and/or insertion of transposable elements in conifers (Nystedt et al., 2013), or incomplete genome assembly because the length of ten homologs proteins (<510 aa) in Norway Spruce was less than that of PTPCs (∼900 aa) in other species. Therefore, ten homologs of PEPC in Norway spruce, which contained the PEPcase superfamily domain, probably performed the PTPC-like physiological functions or was the fragments of PTPC genes. Due to numerous WGDs (Soltis & Soltis, 2016), the PEPC gene family had relatively more copies in angiosperms, especially in maize that contained 22 PTPC genes. Interestingly, moss (*Physcomitrella patens*) also retained 22 PTPC genes, but its sister clades, hornworts (*Anthoceros angustus*) and liverworts (*Marchantia polymorpha*), only had one PTPC gene (Table 1). This extreme difference in gene content corresponded to different adaptation strategies for plant terrestrialization. Mosses underwent WGDs that increase gene-family complexity for coordinating multicellular growth and responding to dehydration (Rensing et al., 2008). However, liverworts have ancient dimorphic sex chromosomes, which may have resulted in a lack of WGDs and reduced proliferation of regulatory genes (Bowman et al., 2017). The genome of *A. angustus* is interestingly simple and has obtained stress-response and metabolic pathway genes through horizontal gene transfer from bacteria or fungi, which probably assisted its survival in a terrestrial environment (Zhang et al., 2020).

PEPC genes displayed highly conserved amino acid sequences in all green plants. Here, all ten motifs were longer than 29 amino acids and was found in more than 104 of the 109 PEPC proteins (Table 2). It is suggested that the PEPC genes were detected reliably, because ultra-conserved motifs indicated similar and/or same function in common (Bejerano et al., 2004). Additionally, the linear order of these motifs, especially in PTPC genes, was identical across all green plants (Fig. 1), and all our predicted motifs with the several functional loci, such as PEP binding site, Mg^2+^ binding site, HCO_3_^−^ binding site, S/A 755 site and Asparate binding site, were also reported previously (Wang et al., 2016), but the motif 10 reported by Wang et al. (2016) was not detected in the present study, which might be only conserved in Angiosperms. These results clearly indicated that the PEPC gene family has been extremely conserved throughout its evolutionary history of more than 500 million years (My), since its origin from algae (Chollet, Vidal & O’Leary, 1996; Svensson, Bläsing & Westhoff, 2003; Darabi & Seddigh, 2018).

However, this conserved gene family has performed hyper-diverse housekeeping functions, including photosynthetic and non-photosynthetic functions, for survival in terrestrial environments (O’Leary, Park & Plaxton William, 2011). To understand the function and molecular mechanisms of the PEPC gene family, the three-dimensional (3D) structure of PEPC proteins was elucidated by X-ray crystallographic analysis (Matsumura et al., 2002; Paulus, Schlieper & Groth, 2013; Connell et al., 2018; González-Segura et al., 2018), which discovered many structure-function relationships of PEPC catalysis, allosteric control, and regulatory phosphorylation (Izui et al., 2004). In order to test if the protein structures evolved convergent in CAM or C_4_ plants, we modeled 3D structures of 90 PTPC proteins using the SWISS-MODEL server (Fig. S1) and predicted three templates of protein structures with higher sequence identity (Table S2): 5vyj.1.A (Fig. 2A) (González-Segura et al., 2018), 3zgb.1.A (Fig. 2B) (Paulus, Schlieper & Groth, 2013) and 5fdn.1.A (Fig. 2C) (Connell et al., 2018). All PTPC proteins were tetrameric enzymes with these three kinds of 3D structures, but the four subunits combined differently. In the widespread 3zgb.1.A template, C4-PEPC isoforms had two amino acid substitutions that increased PEP saturation kinetics and reduced inhibitor affinity, respectively, compared to C3-PEPC isoforms (Bläsing, Westhoff & Svensson, 2000; Paulus, Schlieper & Groth, 2013). Therefore, the efficiency of photosynthetic carbon fixation greatly improved in C_4_ plants. However, there are no evidence for convergent evolution of PEPC protein structures in CAM or C_4_ plants.

### PEPC was convergent in C_4_ photosynthesis but not in CAM photosynthesis

According to the robust phylogenetic tree, PEPC gene family consists of two major subfamilies, PTPC and BTPC, which is consistent with the predictions of the conserved domains (Fig. 1, Table S1). PTPC genes perform the critical roles for initial carbon fixation in C_4_ and CAM photosynthesis (Jiao & Chollet, 1991; Lepiniec et al., 1994; Nimmo, 2000; Deng et al., 2016). Therefore, PTPC gene tree was independently reconstructed and was reconciled with the species tree based on duplication-loss reconciliation (Fig. 3). The reconciliation tree showed that PTPC genes underwent at least 71 duplications and 16 losses in the evolutionary history of our sampled species, which indicated that PTPC genes arose multiple times through frequent duplication events (Fig. 3), potentially caused by WGD in the evolutionary process of green plants, especially in angiosperms (Van de Peer, Mizrachi & Marchal, 2017). After gene duplication, plants could respond to new environments through neo-functionalization of gene copies (Russell, 2003; Cheng et al., 2018). Previous research assumed that PEPC isoforms in C_4_ andCAM species were duplicated from a non-photosynthetic PEPC gene that existed in ancestral C3 species (Svensson, Bläsing & Westhoff, 2003; Christin et al., 2014). Although there are limitations of sampling, our results indicated that no strong association was observed between PEPC gene duplication and CAM/C_4_ evolution (Fig. 3), and the similar results were also found in the study of orchids PEPC genes, in which no correlations between the presence of CAM and gene duplication (Zhang et al., 2016). In other words, PEPC gene duplications may be important for the evolutionary origin of C_4_ and CAM photosynthesis but without clear correlation. In CAM pathway, post-translational regulation of PEPC possibly might play a key role (Jiao & Chollet, 1991; O’Leary, Park & Plaxton William, 2011; Chen et al., 2020b).

To test whether convergent molecular evolution at the amino acid level occurred in PTPC proteins, we performed comprehensive detection of convergent sites in C_4_ and CAM photosynthesis using the PTPC gene tree of green plants with the Profile Change with One Change (PCOC) pipeline, which can detect not only convergent substitutions of amino acids but also convergent shifts that correspond to convergent phenotypic changes (Rey et al., 2018). The results showed two convergent shifts in PTPC proteins that occurred in CAM species and three convergent shifts that occurred in C4 species, the posterior probabilities (pp) for the PCOC model were greater than 0.9 at all convergent shifts (Fig. 4). However, identical convergent substitutions were not detected in clades from both photosynthetic pathways, which indicated that identical convergent molecular evolution at the amino acid level might not occur in all copies of PTPC proteins.

Different isoforms of the PEPC gene family might perform different functions. In addition to photosynthetic functions, PEPC genes also perform hyper-diverse non-photosynthetic functions, such as response to abiotic stress, fruit maturation, seed formation and germination (Lepiniec et al., 1994; Chollet, Vidal & O’Leary, 1996; O’Leary, Park & Plaxton William, 2011; Shi et al., 2015; Wang et al., 2016; Waseem & Ahmad, 2019; Zhao et al., 2019). Maybe only a few of the PEPC isoforms corresponded to convergent evolution of C_4_ and CAM photosynthesis. Therefore, we also detected convergent evolution at sites in different gene groups, in which each convergent phenotypic species retained one clade or one gene copy (one-to-one) (Fig. S2). Interestingly, four identical convergent amino acid sites in one gene group (AhPEPC1.2/ZmPEPC2) were discovered in C_4_ species (Fig. 5), two of which were also reported in previous studies (Bläsing, Westhoff & Svensson, 2000; Christin et al., 2007; Besnard et al., 2009; Paulus, Schlieper & Groth, 2013). The convergent amino acid mutations in the active site Ala774 and the inhibitory site Arg884 were sufficient to switch the photosynthetic function from C_3_ to C_4_ activity (Paulus, Schlieper & Groth, 2013). Due to limited sampling, our results maybe overestimate the number of convergent sites in C_4_ plants, because increased sample size maybe decrease the number of inferred molecular convergence (Thomas, Hahn & Hahn, 2017). Therefore, the two new convergent sites of PEPC gene family in C_4_ species should be verified by further studies with adequate sampling. Several convergent sites reported in the previous studies (Christin et al., 2007; Besnard et al., 2009; Christin et al., 2014; Moreno-Villena et al., 2018) were not detected in the present study, probably because these sites are only convergent in grass.

Previously, Yang et al. (2017) reported a convergent evolution site of PEPC gene in several CAM lineages of angiosperms, except *Ananas comosus*. However, when we detected convergent sites in the one-to-one gene groups of CAM species, no identical convergent sites were found (Fig. S2), which indicated that PEPC genes might not have identical convergent sites that resulted in photosynthetic conversion from the C_3_ to CAM pathway (Wickell et al., 2021).

## Conclusions

PEPC gene family plays a crucial role in C_4_ and CAM photosynthesis and is considered to cause the convergent evolution of these CCMs. In the present study, we detected the convergent amino acid sites of PEPC gene family using relatively limited genomic datasets. In the evolutionary history of the PEPC gene family, gene duplication frequently occurred due to multiple WGD events, but no strong association was observed between PEPC gene duplication and CAM/C_4_ evolution. 3D protein structures of PEPC gene family are also not associated with C_4_ and CAM evolution. Additionally, four sites with convergent substitutions were detected in C4-PEPC isoforms, two of which were key functional positions to switch the photosynthetic pathway from C_3_ to C_4_ activity. However, no convergent sites were detected in CAM-PEPC genes. Our results indicated that convergent molecular substitutions of PEPC genes played key roles for the origin and convergent evolution of C_4_ photosynthesis, but convergent evolution of CAM photosynthesis maybe not caused by convergence at the amino acid level in PEPC proteins. However, our limited sampling maybe affect the inference of molecular convergence. In future, the clearly evolutionary trajectories of PEPC genes will be clarified by more genomic data, which included more species of CAM, C_4_ and C_3_ relatives.

## Supplemental Information

10.7717/peerj.12828/supp-1 Supplemental Information 1 The conserved domains of PEPC genes in this study

10.7717/peerj.12828/supp-2 Supplemental Information 2 Molecular convergent sites of 42 PEPC gene/clade combinations in CAM and C_4_ species 1–33: PEPC gene/clade combinations in CAM plants. 34–42: PEPC gene/clade combinations in C_4_ plants. PCOC: Profile Change with One Change model; PC: Profile Change model; OC: One Change model, all models were in detail explained by Rey et al. (2018). Posterior probabilities (pp) for the PCOC, PC, and OC models are summarized by top box colors, and the amino acid colors correspond to different amino acid equilibrium frequencies (*i.e.*, different profiles) of the Profile Change with One Change model (PCOC model). Aan, *Anthoceros angustus*; Aco, *Ananas comosus*; Afi, *Azolla filiculoides*; Ahy, *Amaranthus hypochondriacus*; Atr, *Amborella trichopoda*; Ath, *Arabidopsis thaliana*; Gmo, *Gnetum montanum*; Isi, *Isoetes sinensis*; Kfe, *Kalanchoe fedtschenkoi*; Mpo, *Marchantia polymorpha*; Osa, *Oryza sativa*; Pab, *Picea abies*; Pbi, *Platycerium bifurcatum*; Ppa, *Physcomitrella patens*; Smo, *Selaginella moellendorffii*; Smu, *Spirogloea muscicola*; Zma, *Zea mays*.

10.7717/peerj.12828/supp-3 Supplemental Information 3 The information of 3D structures in 90 PTPC genes

10.7717/peerj.12828/supp-4 Supplemental Information 4 The 3D structures of 90 PTPC proteins in this study

## Additional Information and Declarations

### DNA Deposition

The following information was supplied regarding the deposition of DNA sequences: The gene family datasets in the present study are available at FigShare: Shu, Jiang-Ping (2021): PEPC gene family data. figshare. Dataset. https://doi.org/10.6084/m9.figshare.16943797.v1.

### Data Availability

The following information was supplied regarding data availability: The PEPC gene family data is available at FigShare: Shu, Jiang-Ping (2021): PEPC gene family data. figshare. Dataset. https://doi.org/10.6084/m9.figshare.16943797.v1. The genomes are available as follows: - Spirogloea muscicola: Chenglab (2019): Genomes of subaerial Zygnematophyceae provide insights into land plant evolution. figshare. Dataset. https://doi.org/10.6084/m9.figshare.9911876.v1. - Marchantia polymorpha: EnsemblPlants, GCA_003032435.1
https://www.ebi.ac.uk/ena/browser/view/GCA_003032435?show=assembly-stats. - Physcomitrella patens: EnsemblPlants, GCA_000002425.2
https://www.ebi.ac.uk/ena/browser/view/GCA_000002425?show=assembly-stats. - Anthoceros angustus: Dryad, Zhang, Jian et al. (2019), The hornwort genome and early land plant evolution, Dryad, Dataset, https://doi.org/10.5061/dryad.msbcc2ftv. - Selaginella moellendorffii: EnsemblPlants, GCA_000143415.1
https://www.ebi.ac.uk/ena/browser/view/GCA_000143415.1. - Azolla filiculoides: https://www.fernbase.org. - Platycerium bifurcatum: NCBI, SRX1098245
https://www.ncbi.nlm.nih.gov/sra/SRX1098245. - Picea abies assembly results: PlantGDB, http://www.plantgdb.org/download/download.php?dir=/Sequence/ESTcontig/Picea_abies. - Gnetum montanum: Dryad, Wan, Tao et al. (2018), Data from: A genome for gnetophytes and early evolution of seed plants, Dryad, Dataset, https://doi.org/10.5061/dryad.0vm37. - Amborella trichopoda: EnsemblPlants, GCA_000471905.1, https://www.ebi.ac.uk/ena/browser/view/GCA_000471905.1. - Ananas comosus: EnsemblPlants, GCA_902162155.1, https://www.ebi.ac.uk/ena/browser/view/GCA_902162155?show=assembly-stats. - Oryza sativa: EnsemblPlants, GCA_001433935.1, https://www.ebi.ac.uk/ena/browser/view/GCA_001433935?show=assembly-stats. - Zea mays: EnsemblPlants, GCA_902167145.1, https://www.ebi.ac.uk/ena/browser/view/GCA_902167145.1. - Arabidopsis thaliana: EnsemblPlants, GCA_000001735.1, https://www.ebi.ac.uk/ena/browser/view/GCA_000001735.1. - Amaranthus hypochondriacus: Phytozome, 459, https://phytozome-next.jgi.doe.gov/info/Ahypochondriacus_v2_1. - Kalanchoe fedtschenkoi: Phytozome, 382, https://phytozome-next.jgi.doe.gov/info/Kfedtschenkoi_v1_1.
