# Supplementary figures and images for: Convergent molecular evolution of phosphoenolpyruvate carboxylase gene family in C4 and crassulacean acid metabolism plants
Source: PeerJ. 2022 Jan 20;10:e12828. doi: 10.7717/peerj.12828 (PMC8784020; doi:10.7717/peerj.12828)

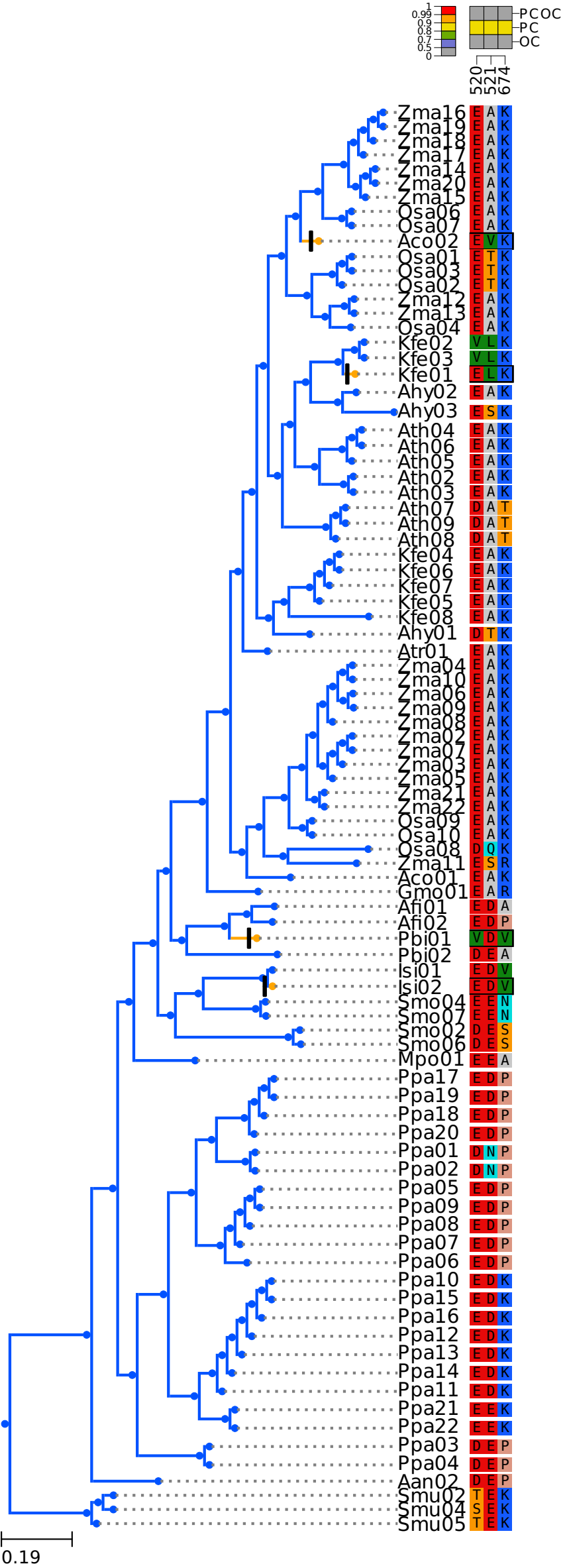

Supplement: Supplemental Information 2 — 1–33: PEPC gene/clade combinations in CAM plants. 34–42: PEPC gene/clade combinations in C4 plants. PCOC: Profile Change with One Change model; PC: Profile Change model; OC: One Change model, all models were in detail explained by Rey et al. (2018). Posterior probabilities (pp) for the PCOC, PC, and OC models are summarized by top box colors, and the amino acid colors correspond to different amino acid equilibrium frequencies (i.e., different profiles) of the Profile Change with One Change model (PCOC model). Aan, Anthoceros angustus; Aco, Ananas comosus; Afi, Azolla filiculoides; Ahy, Amaranthus hypochondriacus; Atr, Amborella trichopoda; Ath, Arabidopsis thaliana; Gmo, Gnetum montanum; Isi, Isoetes sinensis; Kfe, Kalanchoe fedtschenkoi; Mpo, Marchantia polymorpha; Osa, Oryza sativa; Pab, Picea abies; Pbi, Platycerium bifurcatum; Ppa, Physcomitrella patens; Smo, Selaginella moellendorffii; Smu, Spirogloea muscicola; Zma, Zea mays. [file peerj-10-12828-s002.zip › Figure S2/Figure S2-1.pdf]

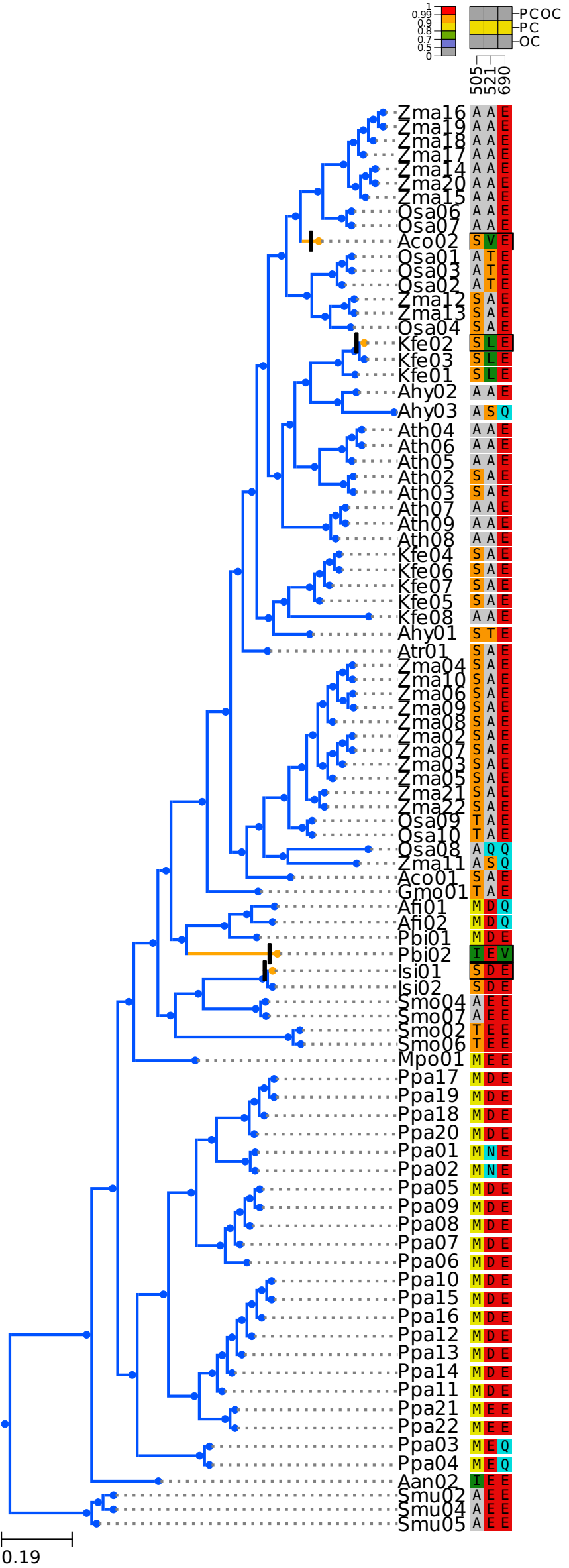

Supplement: Supplemental Information 2 — 1–33: PEPC gene/clade combinations in CAM plants. 34–42: PEPC gene/clade combinations in C4 plants. PCOC: Profile Change with One Change model; PC: Profile Change model; OC: One Change model, all models were in detail explained by Rey et al. (2018). Posterior probabilities (pp) for the PCOC, PC, and OC models are summarized by top box colors, and the amino acid colors correspond to different amino acid equilibrium frequencies (i.e., different profiles) of the Profile Change with One Change model (PCOC model). Aan, Anthoceros angustus; Aco, Ananas comosus; Afi, Azolla filiculoides; Ahy, Amaranthus hypochondriacus; Atr, Amborella trichopoda; Ath, Arabidopsis thaliana; Gmo, Gnetum montanum; Isi, Isoetes sinensis; Kfe, Kalanchoe fedtschenkoi; Mpo, Marchantia polymorpha; Osa, Oryza sativa; Pab, Picea abies; Pbi, Platycerium bifurcatum; Ppa, Physcomitrella patens; Smo, Selaginella moellendorffii; Smu, Spirogloea muscicola; Zma, Zea mays. [file peerj-10-12828-s002.zip › Figure S2/Figure S2-10.pdf]

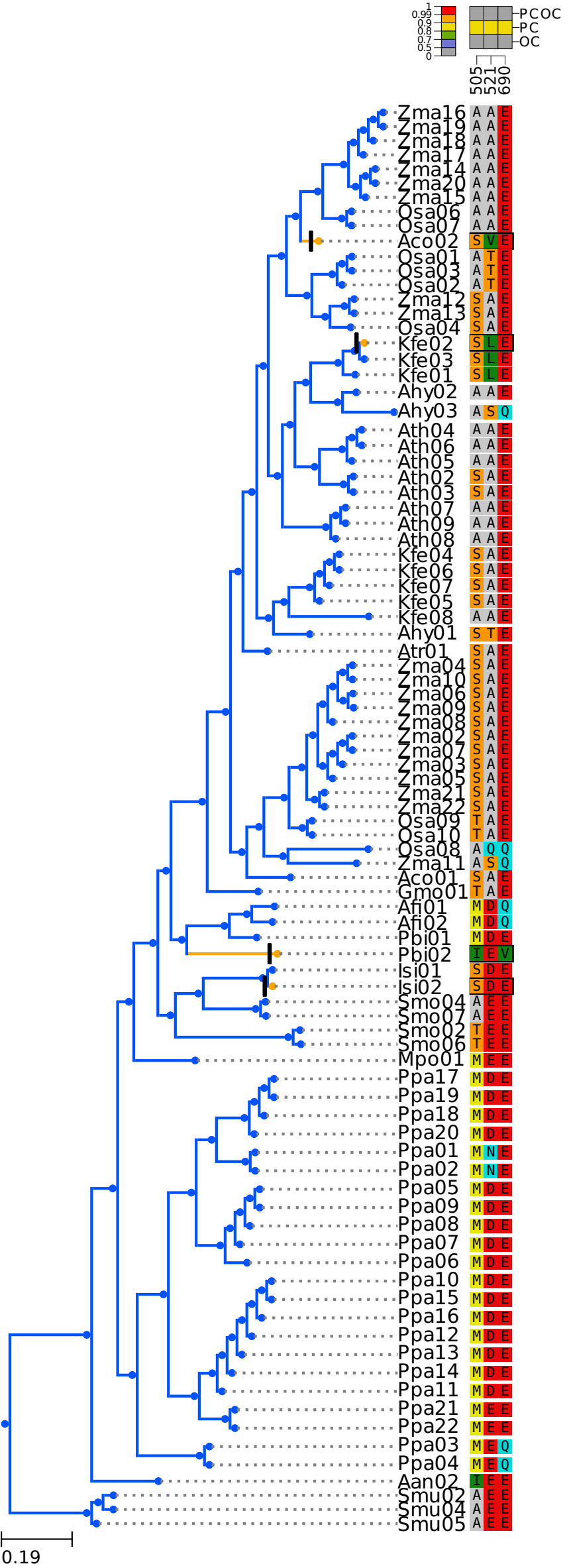

Supplement: Supplemental Information 2 — 1–33: PEPC gene/clade combinations in CAM plants. 34–42: PEPC gene/clade combinations in C4 plants. PCOC: Profile Change with One Change model; PC: Profile Change model; OC: One Change model, all models were in detail explained by Rey et al. (2018). Posterior probabilities (pp) for the PCOC, PC, and OC models are summarized by top box colors, and the amino acid colors correspond to different amino acid equilibrium frequencies (i.e., different profiles) of the Profile Change with One Change model (PCOC model). Aan, Anthoceros angustus; Aco, Ananas comosus; Afi, Azolla filiculoides; Ahy, Amaranthus hypochondriacus; Atr, Amborella trichopoda; Ath, Arabidopsis thaliana; Gmo, Gnetum montanum; Isi, Isoetes sinensis; Kfe, Kalanchoe fedtschenkoi; Mpo, Marchantia polymorpha; Osa, Oryza sativa; Pab, Picea abies; Pbi, Platycerium bifurcatum; Ppa, Physcomitrella patens; Smo, Selaginella moellendorffii; Smu, Spirogloea muscicola; Zma, Zea mays. [file peerj-10-12828-s002.zip › Figure S2/Figure S2-11.pdf]

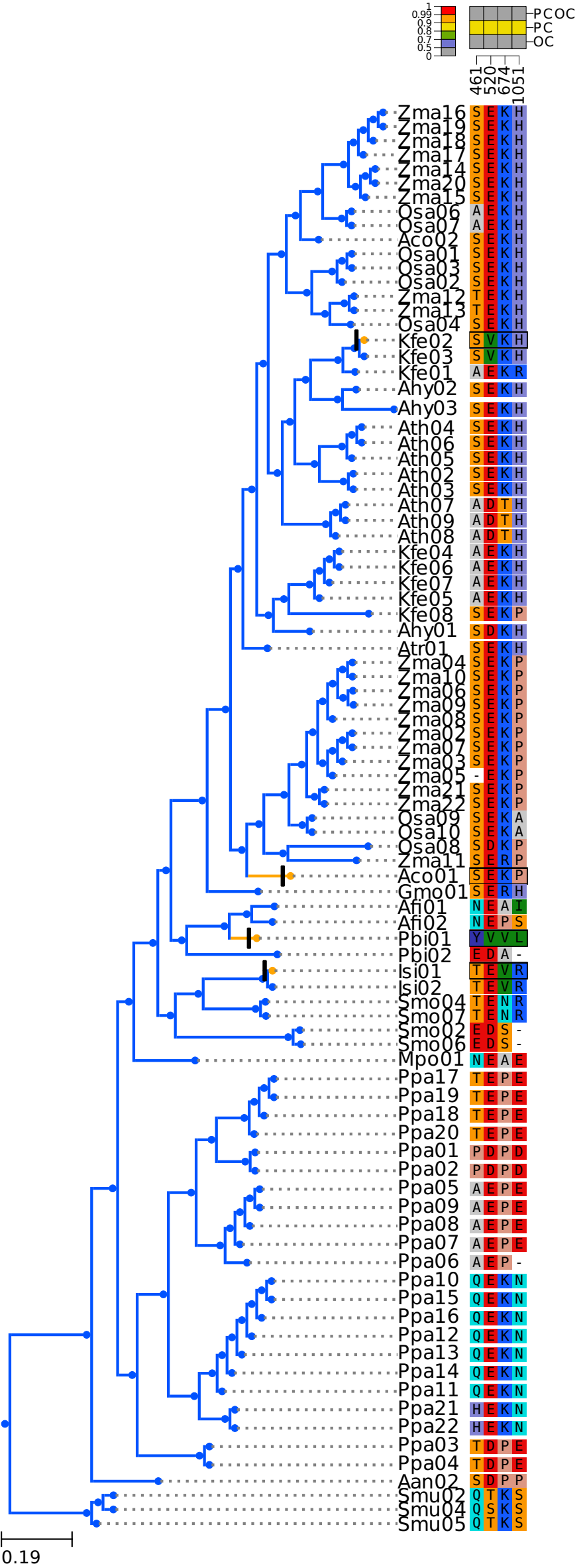

Supplement: Supplemental Information 2 — 1–33: PEPC gene/clade combinations in CAM plants. 34–42: PEPC gene/clade combinations in C4 plants. PCOC: Profile Change with One Change model; PC: Profile Change model; OC: One Change model, all models were in detail explained by Rey et al. (2018). Posterior probabilities (pp) for the PCOC, PC, and OC models are summarized by top box colors, and the amino acid colors correspond to different amino acid equilibrium frequencies (i.e., different profiles) of the Profile Change with One Change model (PCOC model). Aan, Anthoceros angustus; Aco, Ananas comosus; Afi, Azolla filiculoides; Ahy, Amaranthus hypochondriacus; Atr, Amborella trichopoda; Ath, Arabidopsis thaliana; Gmo, Gnetum montanum; Isi, Isoetes sinensis; Kfe, Kalanchoe fedtschenkoi; Mpo, Marchantia polymorpha; Osa, Oryza sativa; Pab, Picea abies; Pbi, Platycerium bifurcatum; Ppa, Physcomitrella patens; Smo, Selaginella moellendorffii; Smu, Spirogloea muscicola; Zma, Zea mays. [file peerj-10-12828-s002.zip › Figure S2/Figure S2-12.pdf]

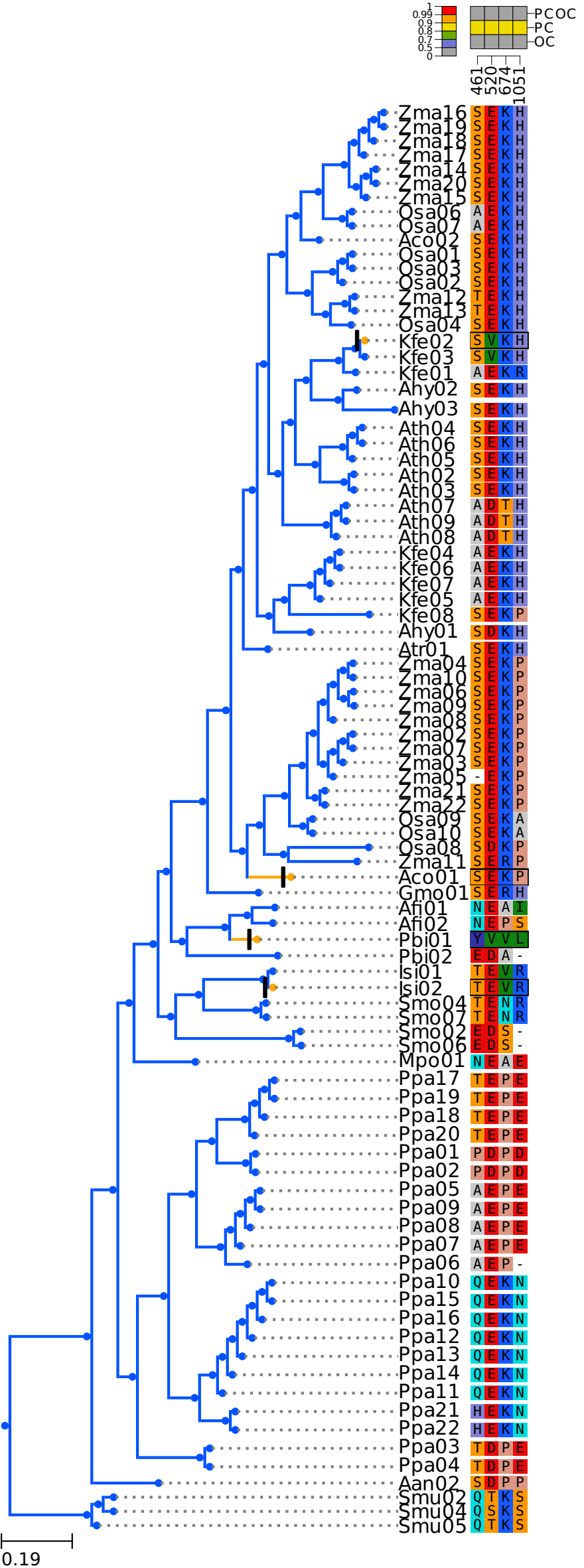

Supplement: Supplemental Information 2 — 1–33: PEPC gene/clade combinations in CAM plants. 34–42: PEPC gene/clade combinations in C4 plants. PCOC: Profile Change with One Change model; PC: Profile Change model; OC: One Change model, all models were in detail explained by Rey et al. (2018). Posterior probabilities (pp) for the PCOC, PC, and OC models are summarized by top box colors, and the amino acid colors correspond to different amino acid equilibrium frequencies (i.e., different profiles) of the Profile Change with One Change model (PCOC model). Aan, Anthoceros angustus; Aco, Ananas comosus; Afi, Azolla filiculoides; Ahy, Amaranthus hypochondriacus; Atr, Amborella trichopoda; Ath, Arabidopsis thaliana; Gmo, Gnetum montanum; Isi, Isoetes sinensis; Kfe, Kalanchoe fedtschenkoi; Mpo, Marchantia polymorpha; Osa, Oryza sativa; Pab, Picea abies; Pbi, Platycerium bifurcatum; Ppa, Physcomitrella patens; Smo, Selaginella moellendorffii; Smu, Spirogloea muscicola; Zma, Zea mays. [file peerj-10-12828-s002.zip › Figure S2/Figure S2-13.pdf]

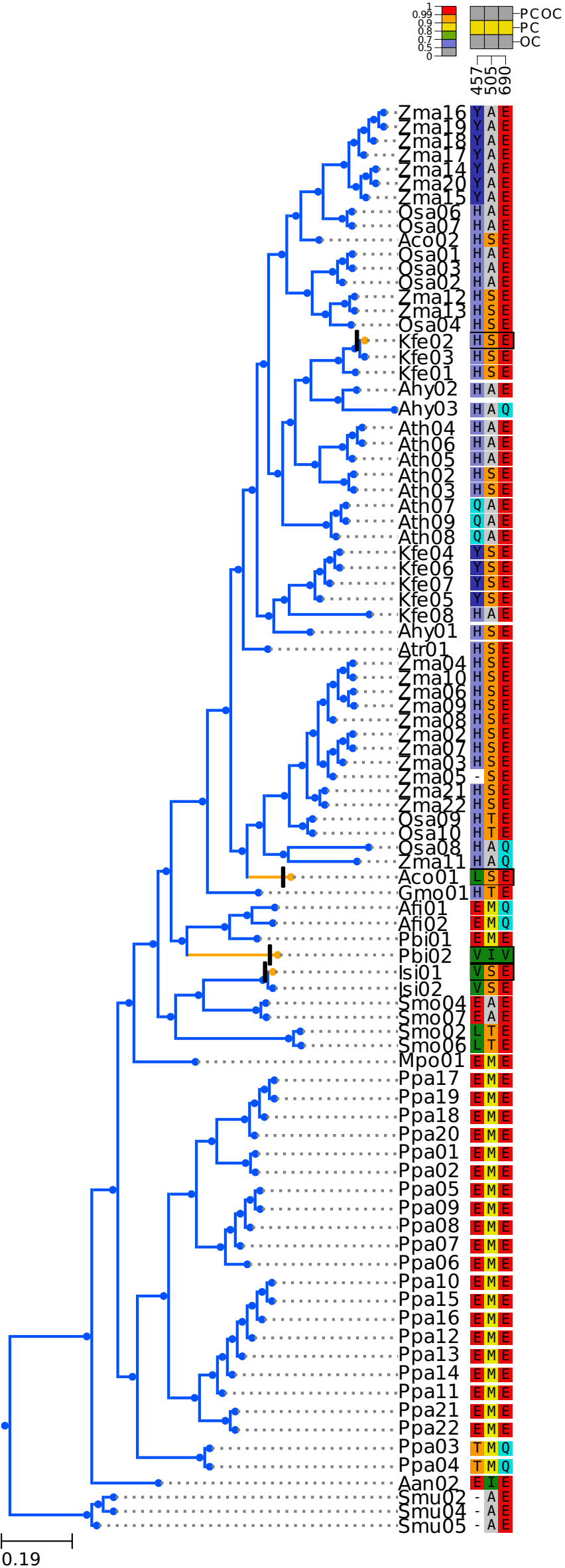

Supplement: Supplemental Information 2 — 1–33: PEPC gene/clade combinations in CAM plants. 34–42: PEPC gene/clade combinations in C4 plants. PCOC: Profile Change with One Change model; PC: Profile Change model; OC: One Change model, all models were in detail explained by Rey et al. (2018). Posterior probabilities (pp) for the PCOC, PC, and OC models are summarized by top box colors, and the amino acid colors correspond to different amino acid equilibrium frequencies (i.e., different profiles) of the Profile Change with One Change model (PCOC model). Aan, Anthoceros angustus; Aco, Ananas comosus; Afi, Azolla filiculoides; Ahy, Amaranthus hypochondriacus; Atr, Amborella trichopoda; Ath, Arabidopsis thaliana; Gmo, Gnetum montanum; Isi, Isoetes sinensis; Kfe, Kalanchoe fedtschenkoi; Mpo, Marchantia polymorpha; Osa, Oryza sativa; Pab, Picea abies; Pbi, Platycerium bifurcatum; Ppa, Physcomitrella patens; Smo, Selaginella moellendorffii; Smu, Spirogloea muscicola; Zma, Zea mays. [file peerj-10-12828-s002.zip › Figure S2/Figure S2-14.pdf]

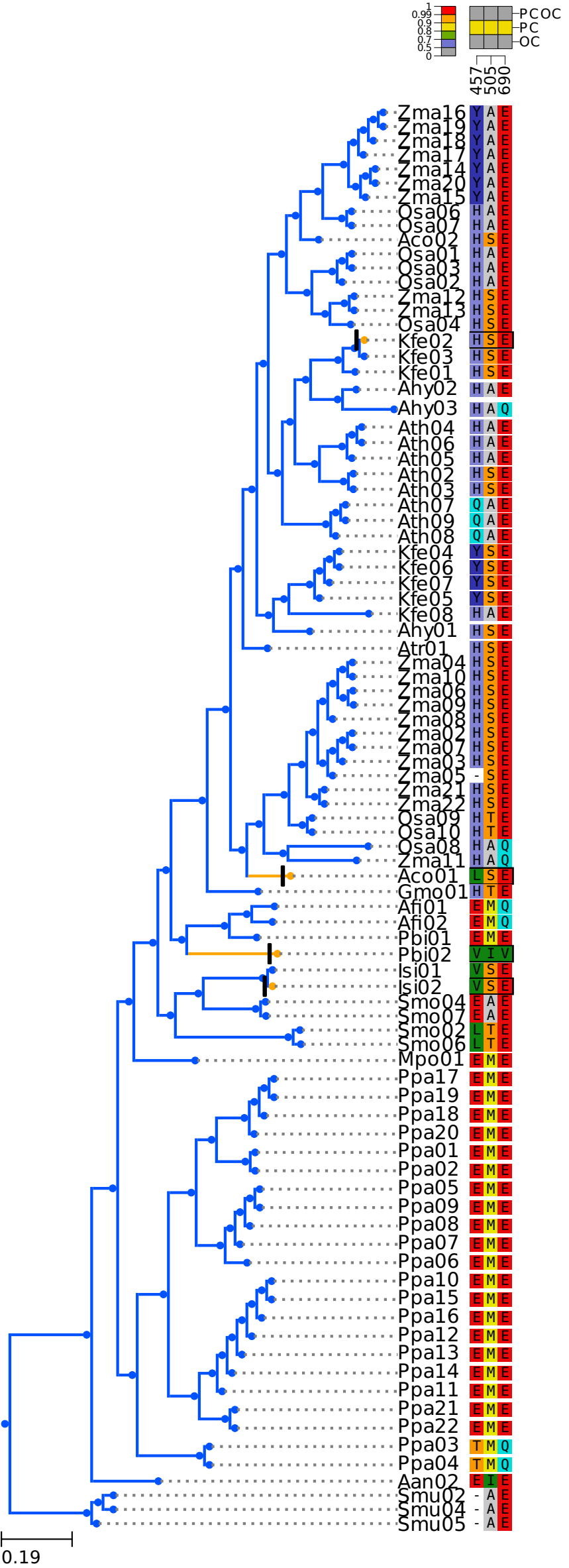

Supplement: Supplemental Information 2 — 1–33: PEPC gene/clade combinations in CAM plants. 34–42: PEPC gene/clade combinations in C4 plants. PCOC: Profile Change with One Change model; PC: Profile Change model; OC: One Change model, all models were in detail explained by Rey et al. (2018). Posterior probabilities (pp) for the PCOC, PC, and OC models are summarized by top box colors, and the amino acid colors correspond to different amino acid equilibrium frequencies (i.e., different profiles) of the Profile Change with One Change model (PCOC model). Aan, Anthoceros angustus; Aco, Ananas comosus; Afi, Azolla filiculoides; Ahy, Amaranthus hypochondriacus; Atr, Amborella trichopoda; Ath, Arabidopsis thaliana; Gmo, Gnetum montanum; Isi, Isoetes sinensis; Kfe, Kalanchoe fedtschenkoi; Mpo, Marchantia polymorpha; Osa, Oryza sativa; Pab, Picea abies; Pbi, Platycerium bifurcatum; Ppa, Physcomitrella patens; Smo, Selaginella moellendorffii; Smu, Spirogloea muscicola; Zma, Zea mays. [file peerj-10-12828-s002.zip › Figure S2/Figure S2-15.pdf]

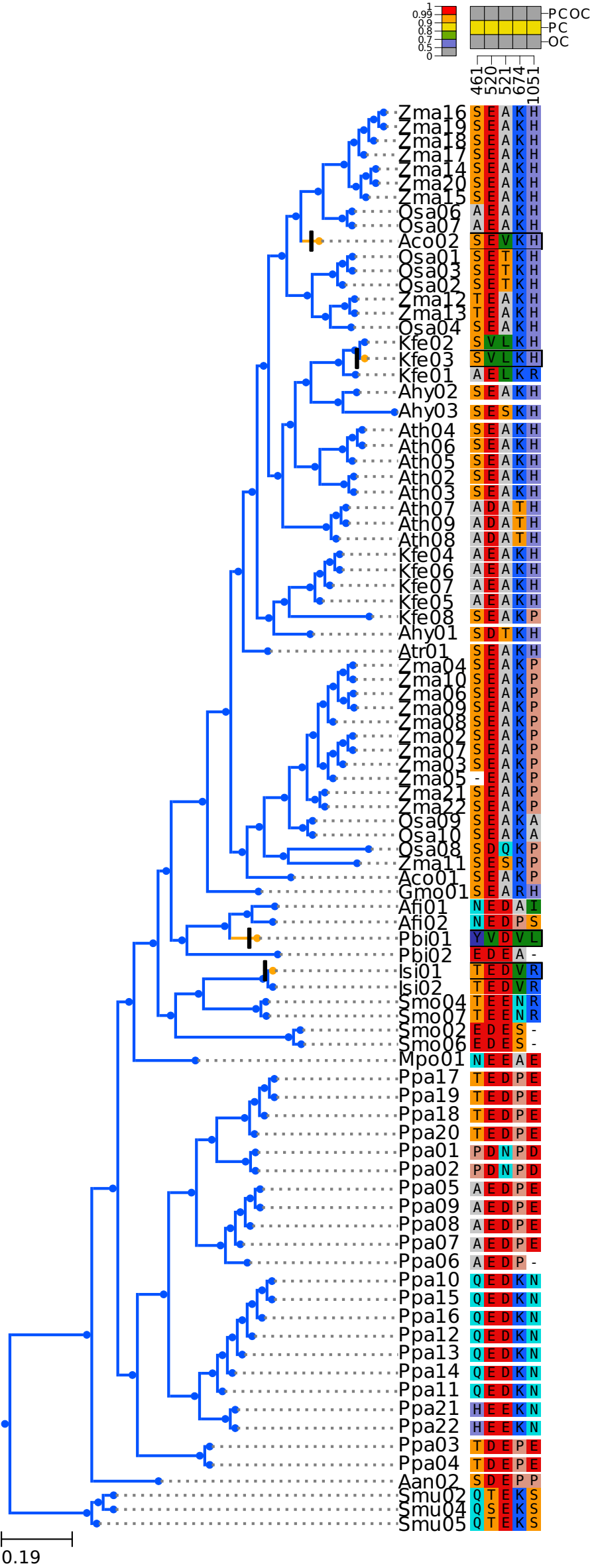

Supplement: Supplemental Information 2 — 1–33: PEPC gene/clade combinations in CAM plants. 34–42: PEPC gene/clade combinations in C4 plants. PCOC: Profile Change with One Change model; PC: Profile Change model; OC: One Change model, all models were in detail explained by Rey et al. (2018). Posterior probabilities (pp) for the PCOC, PC, and OC models are summarized by top box colors, and the amino acid colors correspond to different amino acid equilibrium frequencies (i.e., different profiles) of the Profile Change with One Change model (PCOC model). Aan, Anthoceros angustus; Aco, Ananas comosus; Afi, Azolla filiculoides; Ahy, Amaranthus hypochondriacus; Atr, Amborella trichopoda; Ath, Arabidopsis thaliana; Gmo, Gnetum montanum; Isi, Isoetes sinensis; Kfe, Kalanchoe fedtschenkoi; Mpo, Marchantia polymorpha; Osa, Oryza sativa; Pab, Picea abies; Pbi, Platycerium bifurcatum; Ppa, Physcomitrella patens; Smo, Selaginella moellendorffii; Smu, Spirogloea muscicola; Zma, Zea mays. [file peerj-10-12828-s002.zip › Figure S2/Figure S2-16.pdf]

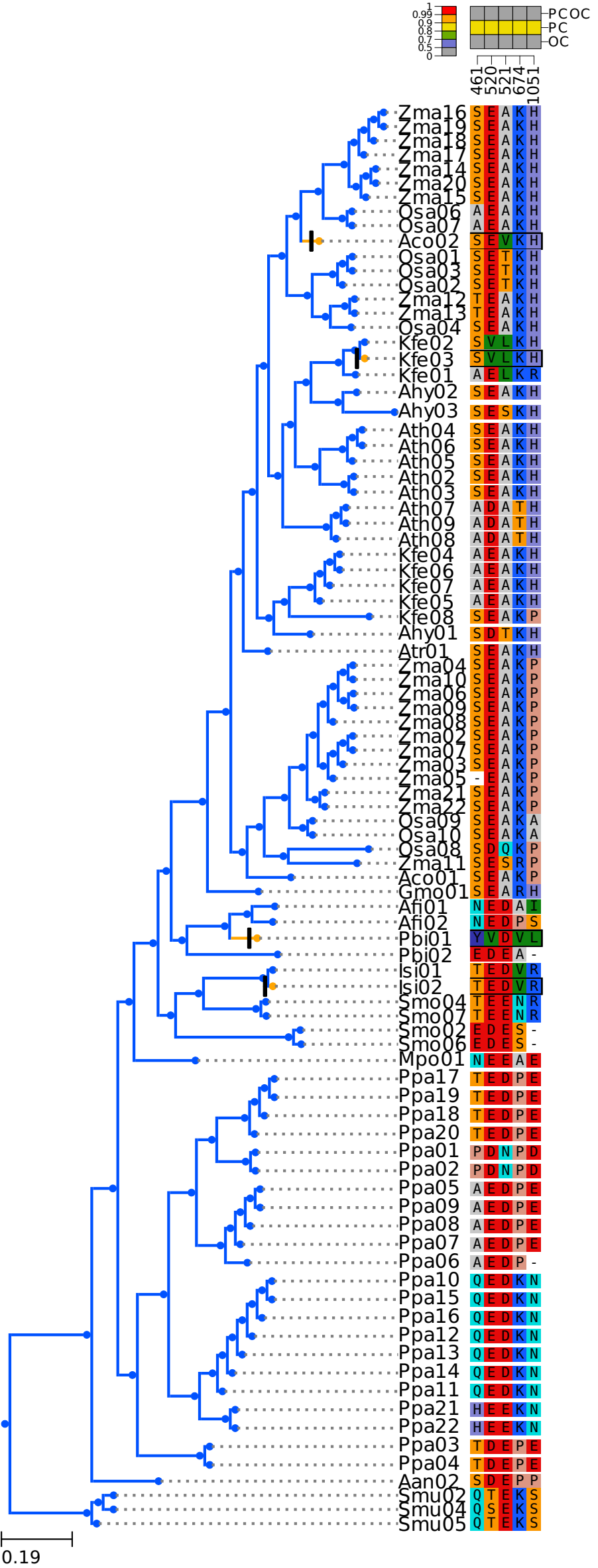

Supplement: Supplemental Information 2 — 1–33: PEPC gene/clade combinations in CAM plants. 34–42: PEPC gene/clade combinations in C4 plants. PCOC: Profile Change with One Change model; PC: Profile Change model; OC: One Change model, all models were in detail explained by Rey et al. (2018). Posterior probabilities (pp) for the PCOC, PC, and OC models are summarized by top box colors, and the amino acid colors correspond to different amino acid equilibrium frequencies (i.e., different profiles) of the Profile Change with One Change model (PCOC model). Aan, Anthoceros angustus; Aco, Ananas comosus; Afi, Azolla filiculoides; Ahy, Amaranthus hypochondriacus; Atr, Amborella trichopoda; Ath, Arabidopsis thaliana; Gmo, Gnetum montanum; Isi, Isoetes sinensis; Kfe, Kalanchoe fedtschenkoi; Mpo, Marchantia polymorpha; Osa, Oryza sativa; Pab, Picea abies; Pbi, Platycerium bifurcatum; Ppa, Physcomitrella patens; Smo, Selaginella moellendorffii; Smu, Spirogloea muscicola; Zma, Zea mays. [file peerj-10-12828-s002.zip › Figure S2/Figure S2-17.pdf]

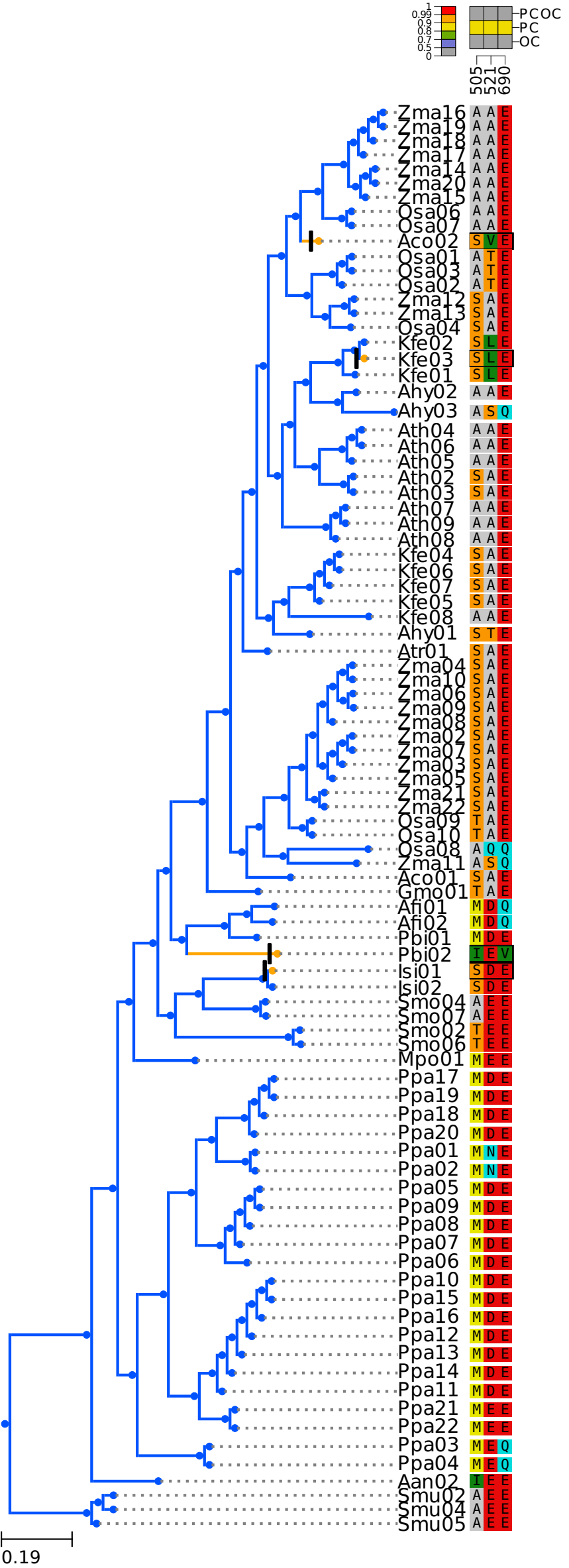

Supplement: Supplemental Information 2 — 1–33: PEPC gene/clade combinations in CAM plants. 34–42: PEPC gene/clade combinations in C4 plants. PCOC: Profile Change with One Change model; PC: Profile Change model; OC: One Change model, all models were in detail explained by Rey et al. (2018). Posterior probabilities (pp) for the PCOC, PC, and OC models are summarized by top box colors, and the amino acid colors correspond to different amino acid equilibrium frequencies (i.e., different profiles) of the Profile Change with One Change model (PCOC model). Aan, Anthoceros angustus; Aco, Ananas comosus; Afi, Azolla filiculoides; Ahy, Amaranthus hypochondriacus; Atr, Amborella trichopoda; Ath, Arabidopsis thaliana; Gmo, Gnetum montanum; Isi, Isoetes sinensis; Kfe, Kalanchoe fedtschenkoi; Mpo, Marchantia polymorpha; Osa, Oryza sativa; Pab, Picea abies; Pbi, Platycerium bifurcatum; Ppa, Physcomitrella patens; Smo, Selaginella moellendorffii; Smu, Spirogloea muscicola; Zma, Zea mays. [file peerj-10-12828-s002.zip › Figure S2/Figure S2-18.pdf]

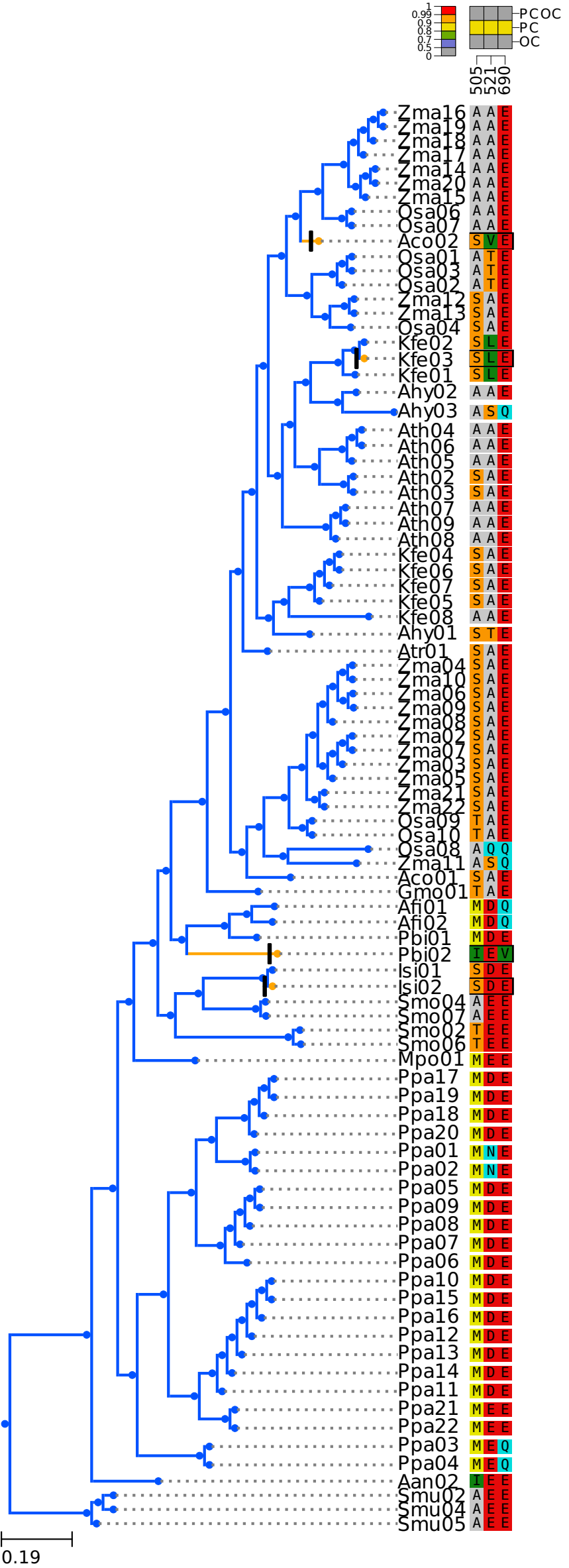

Supplement: Supplemental Information 2 — 1–33: PEPC gene/clade combinations in CAM plants. 34–42: PEPC gene/clade combinations in C4 plants. PCOC: Profile Change with One Change model; PC: Profile Change model; OC: One Change model, all models were in detail explained by Rey et al. (2018). Posterior probabilities (pp) for the PCOC, PC, and OC models are summarized by top box colors, and the amino acid colors correspond to different amino acid equilibrium frequencies (i.e., different profiles) of the Profile Change with One Change model (PCOC model). Aan, Anthoceros angustus; Aco, Ananas comosus; Afi, Azolla filiculoides; Ahy, Amaranthus hypochondriacus; Atr, Amborella trichopoda; Ath, Arabidopsis thaliana; Gmo, Gnetum montanum; Isi, Isoetes sinensis; Kfe, Kalanchoe fedtschenkoi; Mpo, Marchantia polymorpha; Osa, Oryza sativa; Pab, Picea abies; Pbi, Platycerium bifurcatum; Ppa, Physcomitrella patens; Smo, Selaginella moellendorffii; Smu, Spirogloea muscicola; Zma, Zea mays. [file peerj-10-12828-s002.zip › Figure S2/Figure S2-19.pdf]

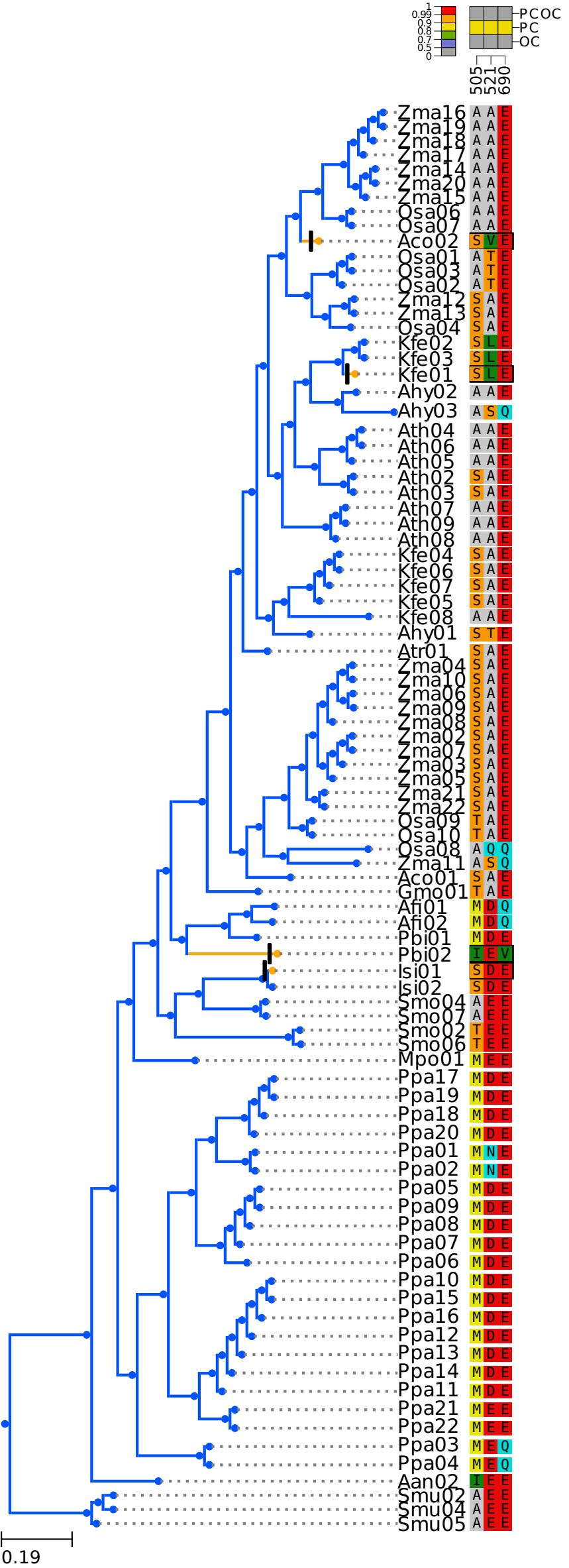

Supplement: Supplemental Information 2 — 1–33: PEPC gene/clade combinations in CAM plants. 34–42: PEPC gene/clade combinations in C4 plants. PCOC: Profile Change with One Change model; PC: Profile Change model; OC: One Change model, all models were in detail explained by Rey et al. (2018). Posterior probabilities (pp) for the PCOC, PC, and OC models are summarized by top box colors, and the amino acid colors correspond to different amino acid equilibrium frequencies (i.e., different profiles) of the Profile Change with One Change model (PCOC model). Aan, Anthoceros angustus; Aco, Ananas comosus; Afi, Azolla filiculoides; Ahy, Amaranthus hypochondriacus; Atr, Amborella trichopoda; Ath, Arabidopsis thaliana; Gmo, Gnetum montanum; Isi, Isoetes sinensis; Kfe, Kalanchoe fedtschenkoi; Mpo, Marchantia polymorpha; Osa, Oryza sativa; Pab, Picea abies; Pbi, Platycerium bifurcatum; Ppa, Physcomitrella patens; Smo, Selaginella moellendorffii; Smu, Spirogloea muscicola; Zma, Zea mays. [file peerj-10-12828-s002.zip › Figure S2/Figure S2-2.pdf]

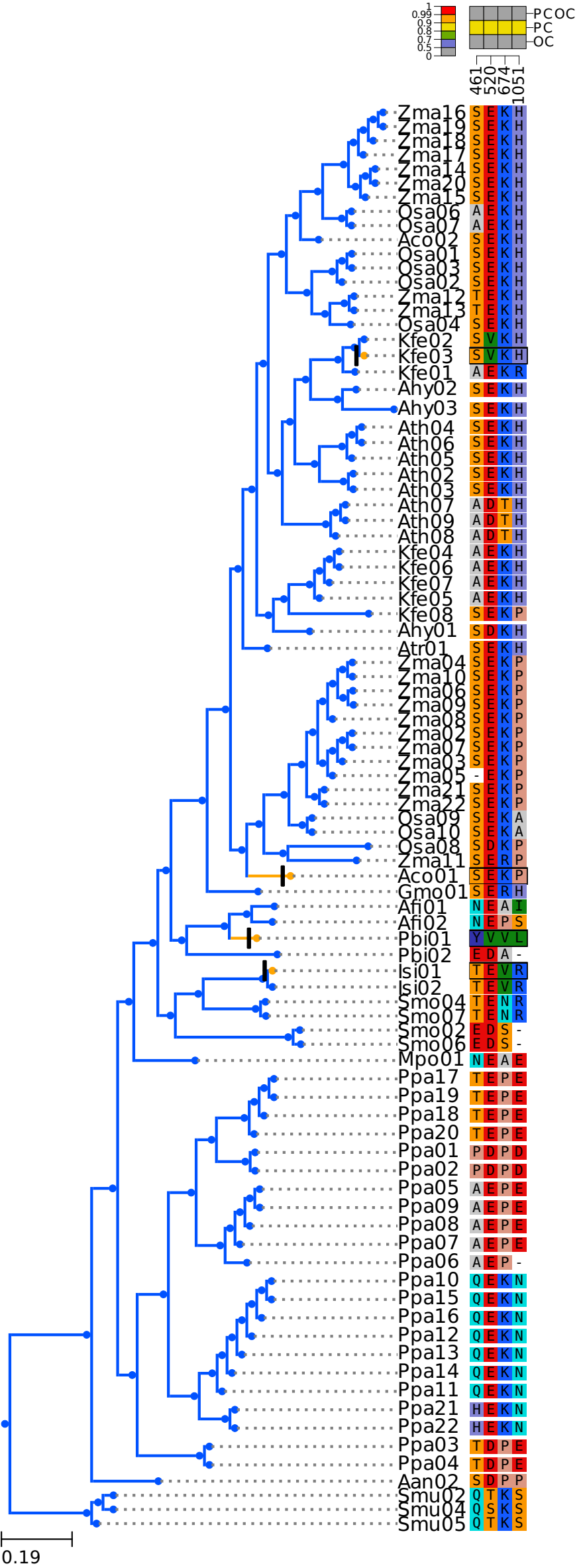

Supplement: Supplemental Information 2 — 1–33: PEPC gene/clade combinations in CAM plants. 34–42: PEPC gene/clade combinations in C4 plants. PCOC: Profile Change with One Change model; PC: Profile Change model; OC: One Change model, all models were in detail explained by Rey et al. (2018). Posterior probabilities (pp) for the PCOC, PC, and OC models are summarized by top box colors, and the amino acid colors correspond to different amino acid equilibrium frequencies (i.e., different profiles) of the Profile Change with One Change model (PCOC model). Aan, Anthoceros angustus; Aco, Ananas comosus; Afi, Azolla filiculoides; Ahy, Amaranthus hypochondriacus; Atr, Amborella trichopoda; Ath, Arabidopsis thaliana; Gmo, Gnetum montanum; Isi, Isoetes sinensis; Kfe, Kalanchoe fedtschenkoi; Mpo, Marchantia polymorpha; Osa, Oryza sativa; Pab, Picea abies; Pbi, Platycerium bifurcatum; Ppa, Physcomitrella patens; Smo, Selaginella moellendorffii; Smu, Spirogloea muscicola; Zma, Zea mays. [file peerj-10-12828-s002.zip › Figure S2/Figure S2-20.pdf]

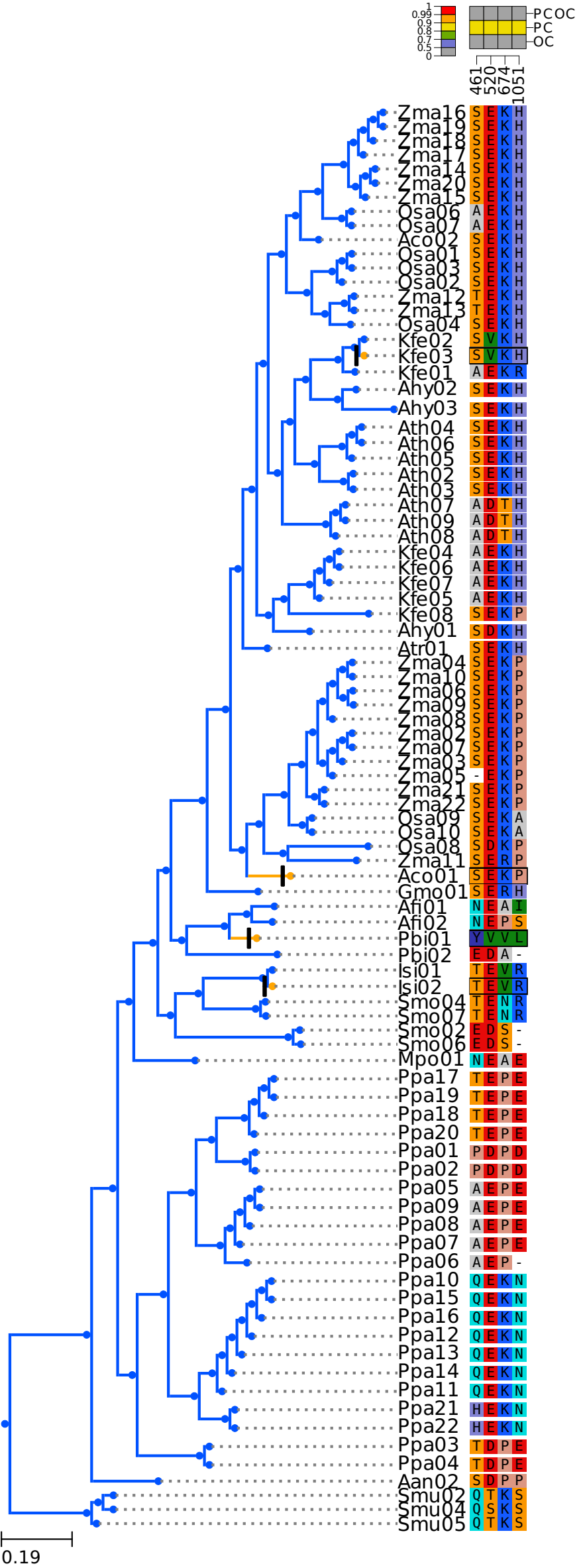

Supplement: Supplemental Information 2 — 1–33: PEPC gene/clade combinations in CAM plants. 34–42: PEPC gene/clade combinations in C4 plants. PCOC: Profile Change with One Change model; PC: Profile Change model; OC: One Change model, all models were in detail explained by Rey et al. (2018). Posterior probabilities (pp) for the PCOC, PC, and OC models are summarized by top box colors, and the amino acid colors correspond to different amino acid equilibrium frequencies (i.e., different profiles) of the Profile Change with One Change model (PCOC model). Aan, Anthoceros angustus; Aco, Ananas comosus; Afi, Azolla filiculoides; Ahy, Amaranthus hypochondriacus; Atr, Amborella trichopoda; Ath, Arabidopsis thaliana; Gmo, Gnetum montanum; Isi, Isoetes sinensis; Kfe, Kalanchoe fedtschenkoi; Mpo, Marchantia polymorpha; Osa, Oryza sativa; Pab, Picea abies; Pbi, Platycerium bifurcatum; Ppa, Physcomitrella patens; Smo, Selaginella moellendorffii; Smu, Spirogloea muscicola; Zma, Zea mays. [file peerj-10-12828-s002.zip › Figure S2/Figure S2-21.pdf]

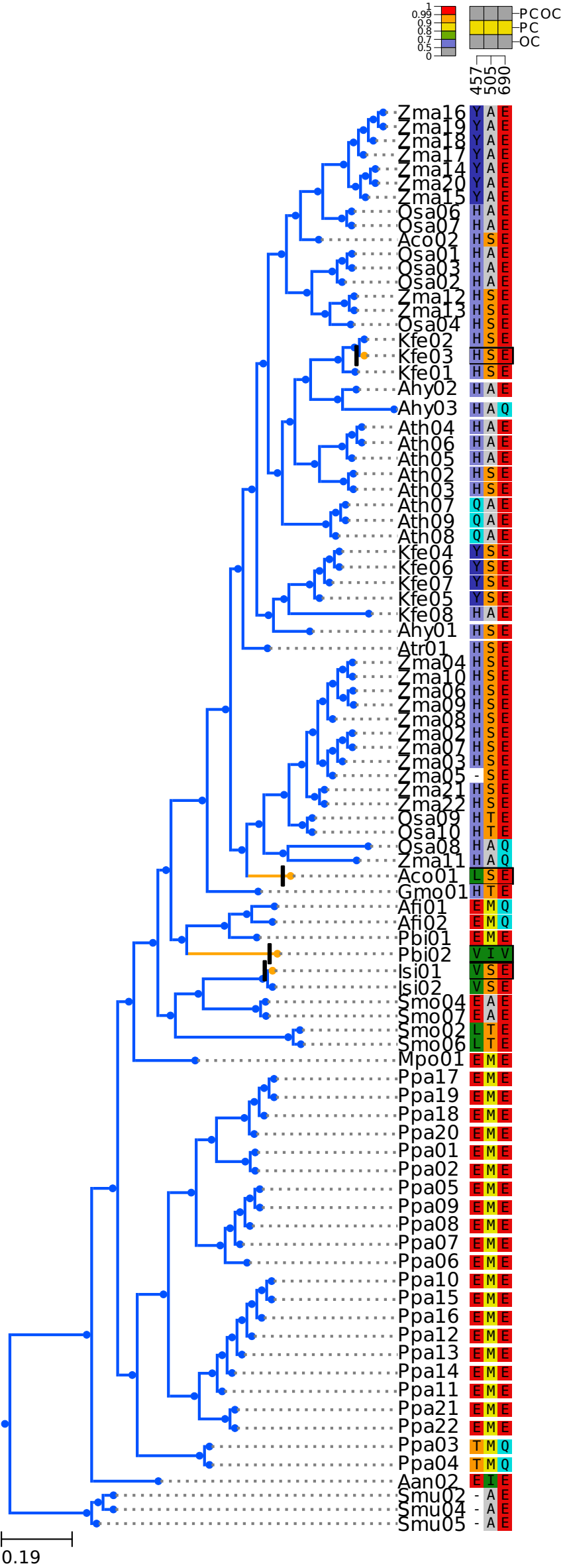

Supplement: Supplemental Information 2 — 1–33: PEPC gene/clade combinations in CAM plants. 34–42: PEPC gene/clade combinations in C4 plants. PCOC: Profile Change with One Change model; PC: Profile Change model; OC: One Change model, all models were in detail explained by Rey et al. (2018). Posterior probabilities (pp) for the PCOC, PC, and OC models are summarized by top box colors, and the amino acid colors correspond to different amino acid equilibrium frequencies (i.e., different profiles) of the Profile Change with One Change model (PCOC model). Aan, Anthoceros angustus; Aco, Ananas comosus; Afi, Azolla filiculoides; Ahy, Amaranthus hypochondriacus; Atr, Amborella trichopoda; Ath, Arabidopsis thaliana; Gmo, Gnetum montanum; Isi, Isoetes sinensis; Kfe, Kalanchoe fedtschenkoi; Mpo, Marchantia polymorpha; Osa, Oryza sativa; Pab, Picea abies; Pbi, Platycerium bifurcatum; Ppa, Physcomitrella patens; Smo, Selaginella moellendorffii; Smu, Spirogloea muscicola; Zma, Zea mays. [file peerj-10-12828-s002.zip › Figure S2/Figure S2-22.pdf]

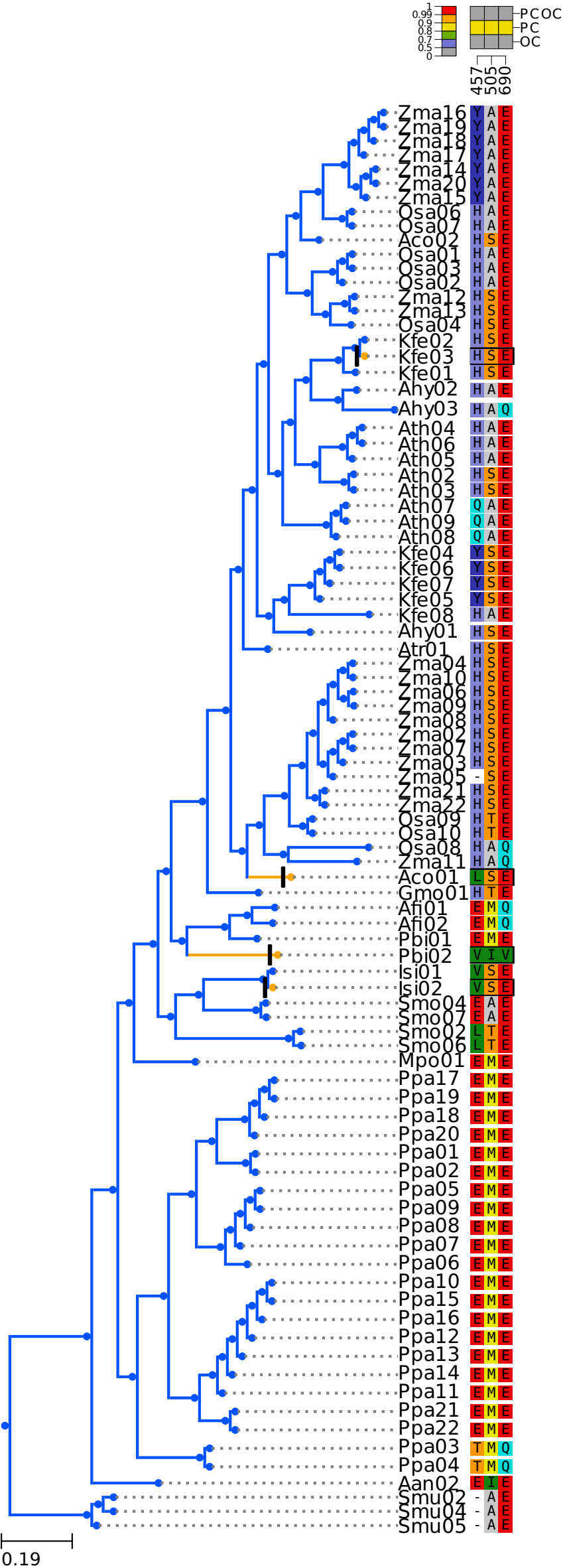

Supplement: Supplemental Information 2 — 1–33: PEPC gene/clade combinations in CAM plants. 34–42: PEPC gene/clade combinations in C4 plants. PCOC: Profile Change with One Change model; PC: Profile Change model; OC: One Change model, all models were in detail explained by Rey et al. (2018). Posterior probabilities (pp) for the PCOC, PC, and OC models are summarized by top box colors, and the amino acid colors correspond to different amino acid equilibrium frequencies (i.e., different profiles) of the Profile Change with One Change model (PCOC model). Aan, Anthoceros angustus; Aco, Ananas comosus; Afi, Azolla filiculoides; Ahy, Amaranthus hypochondriacus; Atr, Amborella trichopoda; Ath, Arabidopsis thaliana; Gmo, Gnetum montanum; Isi, Isoetes sinensis; Kfe, Kalanchoe fedtschenkoi; Mpo, Marchantia polymorpha; Osa, Oryza sativa; Pab, Picea abies; Pbi, Platycerium bifurcatum; Ppa, Physcomitrella patens; Smo, Selaginella moellendorffii; Smu, Spirogloea muscicola; Zma, Zea mays. [file peerj-10-12828-s002.zip › Figure S2/Figure S2-23.pdf]

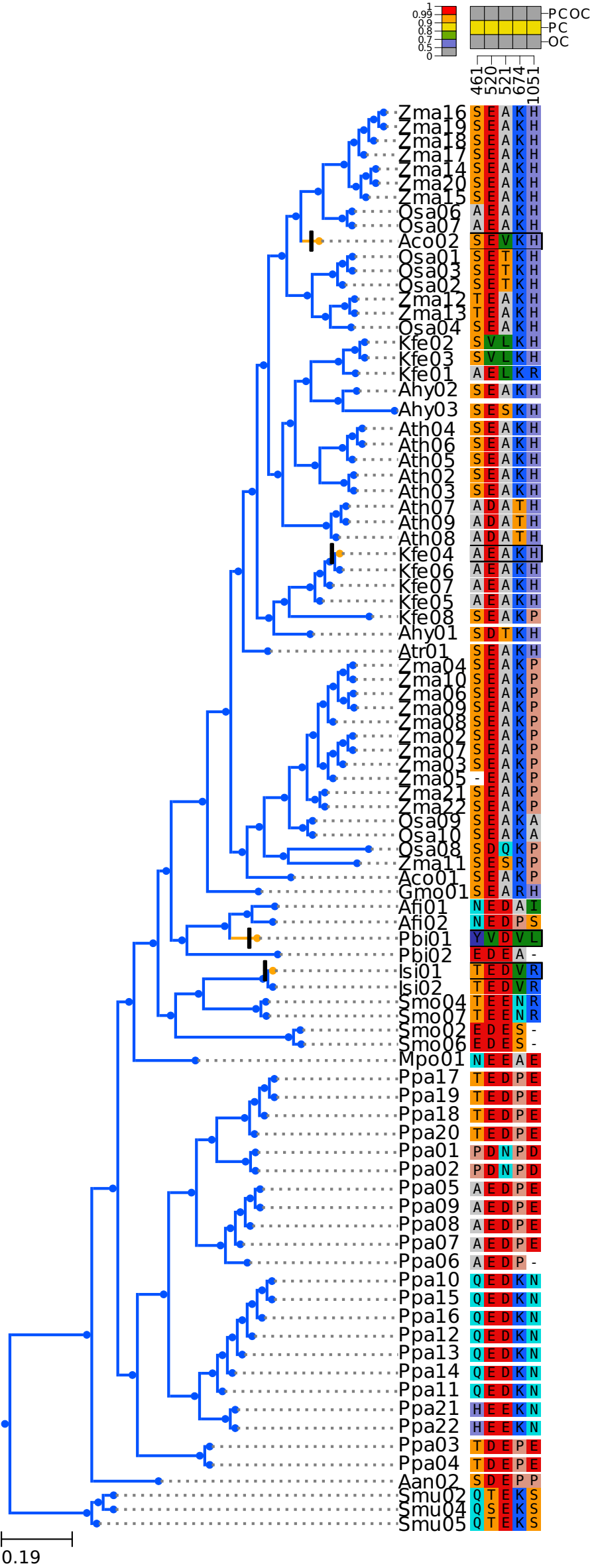

Supplement: Supplemental Information 2 — 1–33: PEPC gene/clade combinations in CAM plants. 34–42: PEPC gene/clade combinations in C4 plants. PCOC: Profile Change with One Change model; PC: Profile Change model; OC: One Change model, all models were in detail explained by Rey et al. (2018). Posterior probabilities (pp) for the PCOC, PC, and OC models are summarized by top box colors, and the amino acid colors correspond to different amino acid equilibrium frequencies (i.e., different profiles) of the Profile Change with One Change model (PCOC model). Aan, Anthoceros angustus; Aco, Ananas comosus; Afi, Azolla filiculoides; Ahy, Amaranthus hypochondriacus; Atr, Amborella trichopoda; Ath, Arabidopsis thaliana; Gmo, Gnetum montanum; Isi, Isoetes sinensis; Kfe, Kalanchoe fedtschenkoi; Mpo, Marchantia polymorpha; Osa, Oryza sativa; Pab, Picea abies; Pbi, Platycerium bifurcatum; Ppa, Physcomitrella patens; Smo, Selaginella moellendorffii; Smu, Spirogloea muscicola; Zma, Zea mays. [file peerj-10-12828-s002.zip › Figure S2/Figure S2-24.pdf]

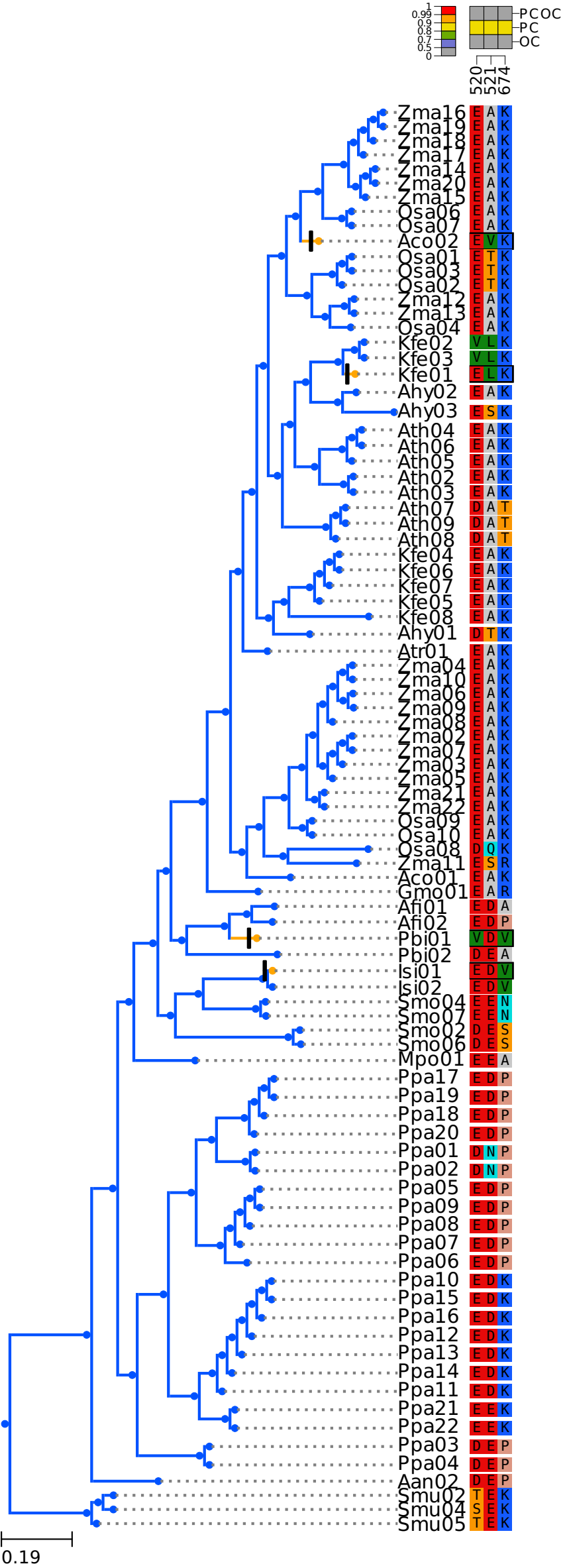

Supplement: Supplemental Information 2 — 1–33: PEPC gene/clade combinations in CAM plants. 34–42: PEPC gene/clade combinations in C4 plants. PCOC: Profile Change with One Change model; PC: Profile Change model; OC: One Change model, all models were in detail explained by Rey et al. (2018). Posterior probabilities (pp) for the PCOC, PC, and OC models are summarized by top box colors, and the amino acid colors correspond to different amino acid equilibrium frequencies (i.e., different profiles) of the Profile Change with One Change model (PCOC model). Aan, Anthoceros angustus; Aco, Ananas comosus; Afi, Azolla filiculoides; Ahy, Amaranthus hypochondriacus; Atr, Amborella trichopoda; Ath, Arabidopsis thaliana; Gmo, Gnetum montanum; Isi, Isoetes sinensis; Kfe, Kalanchoe fedtschenkoi; Mpo, Marchantia polymorpha; Osa, Oryza sativa; Pab, Picea abies; Pbi, Platycerium bifurcatum; Ppa, Physcomitrella patens; Smo, Selaginella moellendorffii; Smu, Spirogloea muscicola; Zma, Zea mays. [file peerj-10-12828-s002.zip › Figure S2/Figure S2-25.pdf]

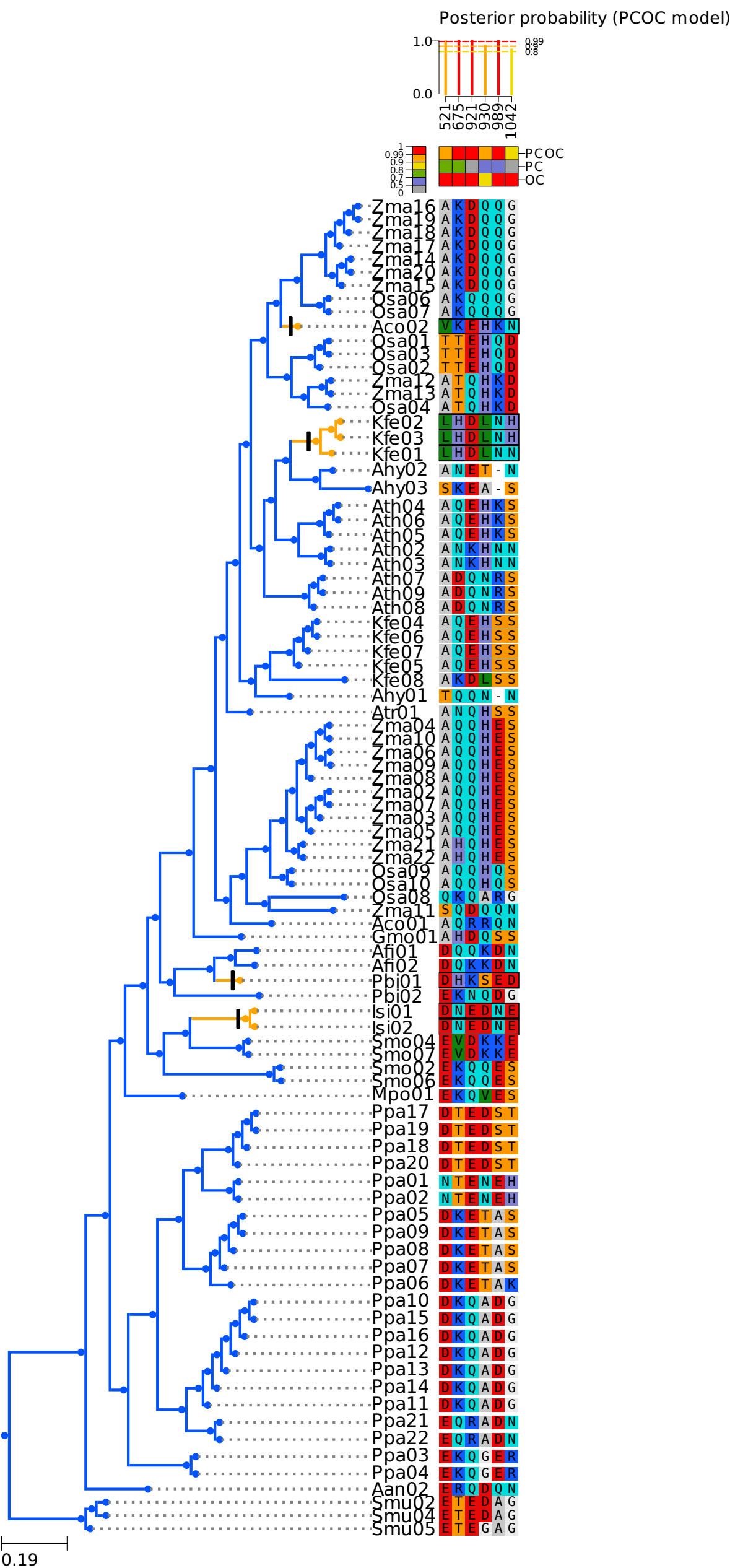

Supplement: Supplemental Information 2 — 1–33: PEPC gene/clade combinations in CAM plants. 34–42: PEPC gene/clade combinations in C4 plants. PCOC: Profile Change with One Change model; PC: Profile Change model; OC: One Change model, all models were in detail explained by Rey et al. (2018). Posterior probabilities (pp) for the PCOC, PC, and OC models are summarized by top box colors, and the amino acid colors correspond to different amino acid equilibrium frequencies (i.e., different profiles) of the Profile Change with One Change model (PCOC model). Aan, Anthoceros angustus; Aco, Ananas comosus; Afi, Azolla filiculoides; Ahy, Amaranthus hypochondriacus; Atr, Amborella trichopoda; Ath, Arabidopsis thaliana; Gmo, Gnetum montanum; Isi, Isoetes sinensis; Kfe, Kalanchoe fedtschenkoi; Mpo, Marchantia polymorpha; Osa, Oryza sativa; Pab, Picea abies; Pbi, Platycerium bifurcatum; Ppa, Physcomitrella patens; Smo, Selaginella moellendorffii; Smu, Spirogloea muscicola; Zma, Zea mays. [file peerj-10-12828-s002.zip › Figure S2/Figure S2-26.pdf]

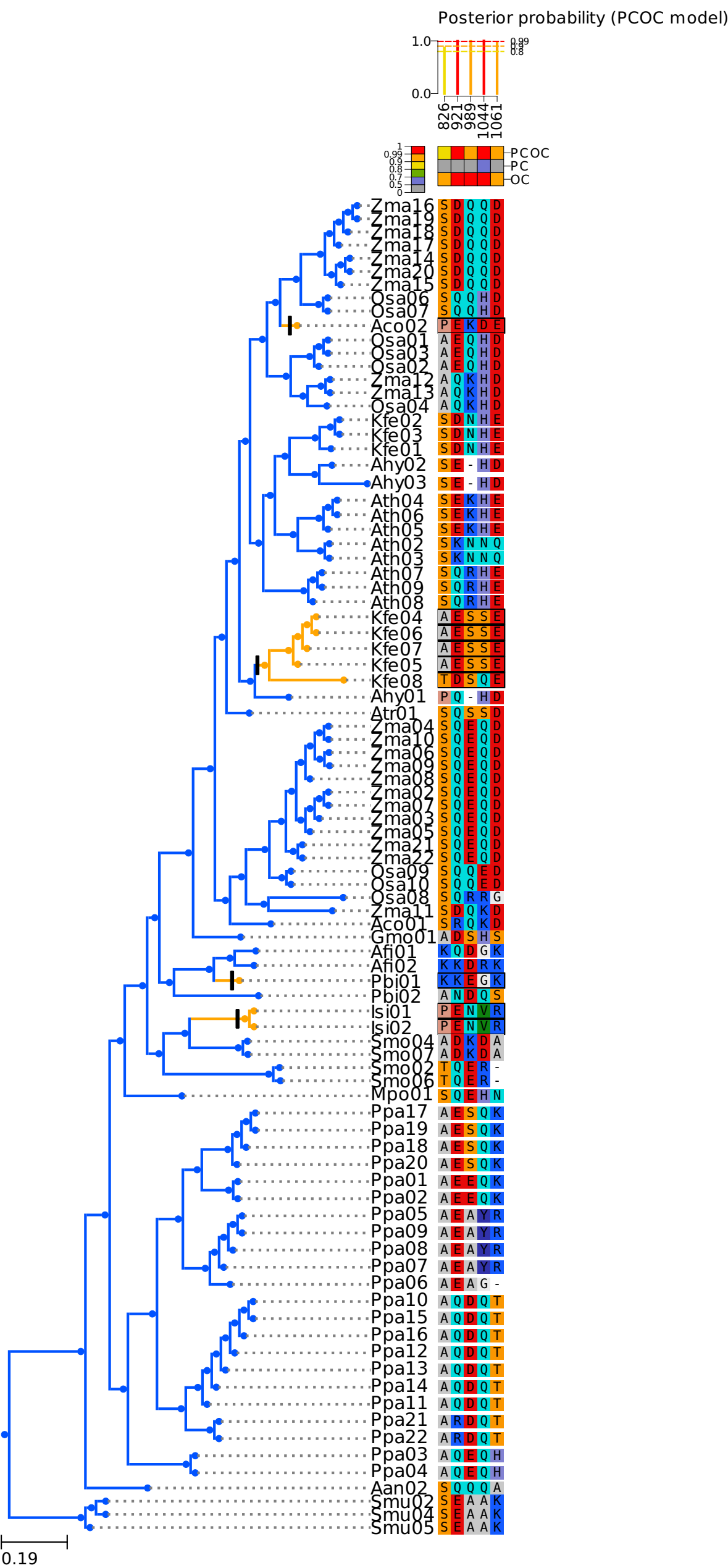

Supplement: Supplemental Information 2 — 1–33: PEPC gene/clade combinations in CAM plants. 34–42: PEPC gene/clade combinations in C4 plants. PCOC: Profile Change with One Change model; PC: Profile Change model; OC: One Change model, all models were in detail explained by Rey et al. (2018). Posterior probabilities (pp) for the PCOC, PC, and OC models are summarized by top box colors, and the amino acid colors correspond to different amino acid equilibrium frequencies (i.e., different profiles) of the Profile Change with One Change model (PCOC model). Aan, Anthoceros angustus; Aco, Ananas comosus; Afi, Azolla filiculoides; Ahy, Amaranthus hypochondriacus; Atr, Amborella trichopoda; Ath, Arabidopsis thaliana; Gmo, Gnetum montanum; Isi, Isoetes sinensis; Kfe, Kalanchoe fedtschenkoi; Mpo, Marchantia polymorpha; Osa, Oryza sativa; Pab, Picea abies; Pbi, Platycerium bifurcatum; Ppa, Physcomitrella patens; Smo, Selaginella moellendorffii; Smu, Spirogloea muscicola; Zma, Zea mays. [file peerj-10-12828-s002.zip › Figure S2/Figure S2-27.pdf]

Posterior probability (PCOC model)

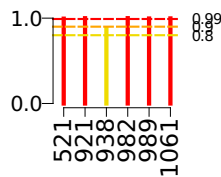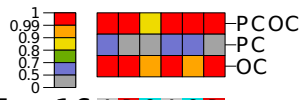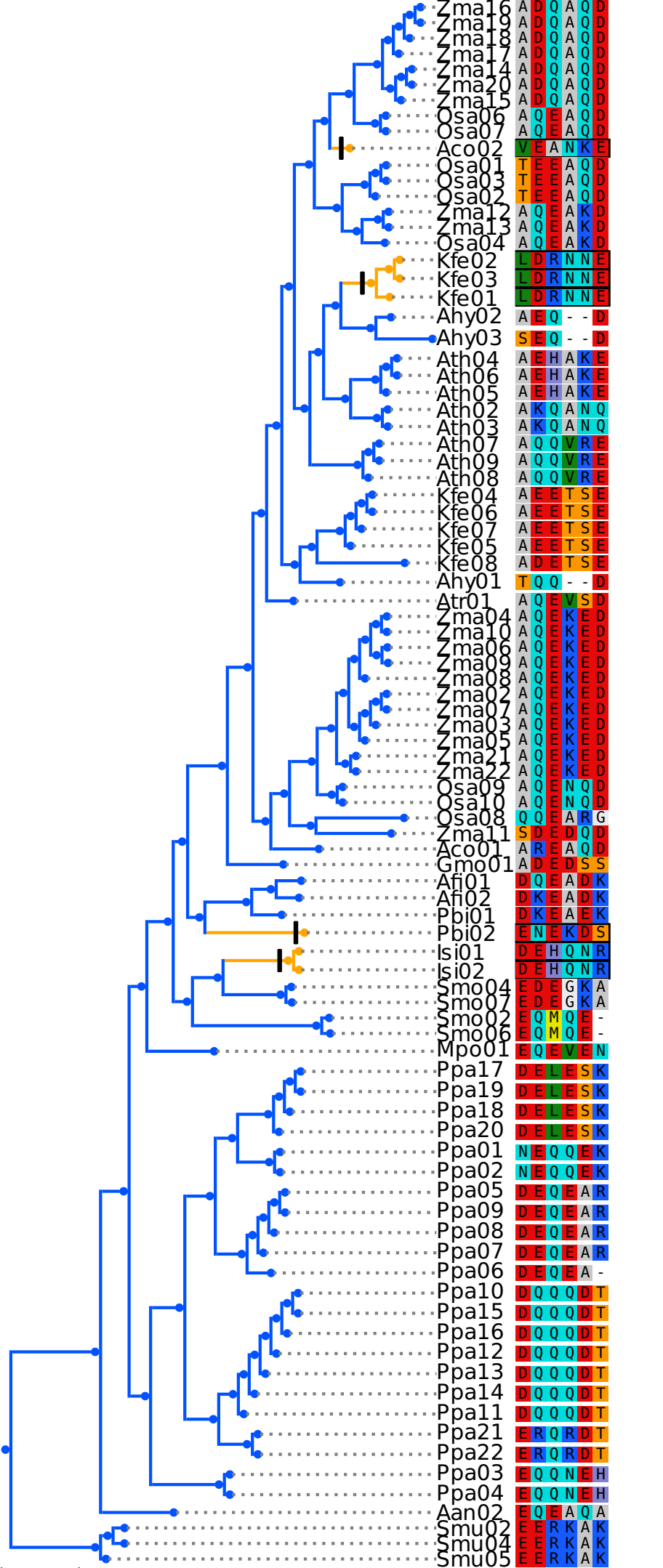

0.19

Supplement: Supplemental Information 2 — 1–33: PEPC gene/clade combinations in CAM plants. 34–42: PEPC gene/clade combinations in C4 plants. PCOC: Profile Change with One Change model; PC: Profile Change model; OC: One Change model, all models were in detail explained by Rey et al. (2018). Posterior probabilities (pp) for the PCOC, PC, and OC models are summarized by top box colors, and the amino acid colors correspond to different amino acid equilibrium frequencies (i.e., different profiles) of the Profile Change with One Change model (PCOC model). Aan, Anthoceros angustus; Aco, Ananas comosus; Afi, Azolla filiculoides; Ahy, Amaranthus hypochondriacus; Atr, Amborella trichopoda; Ath, Arabidopsis thaliana; Gmo, Gnetum montanum; Isi, Isoetes sinensis; Kfe, Kalanchoe fedtschenkoi; Mpo, Marchantia polymorpha; Osa, Oryza sativa; Pab, Picea abies; Pbi, Platycerium bifurcatum; Ppa, Physcomitrella patens; Smo, Selaginella moellendorffii; Smu, Spirogloea muscicola; Zma, Zea mays. [file peerj-10-12828-s002.zip › Figure S2/Figure S2-28.pdf]

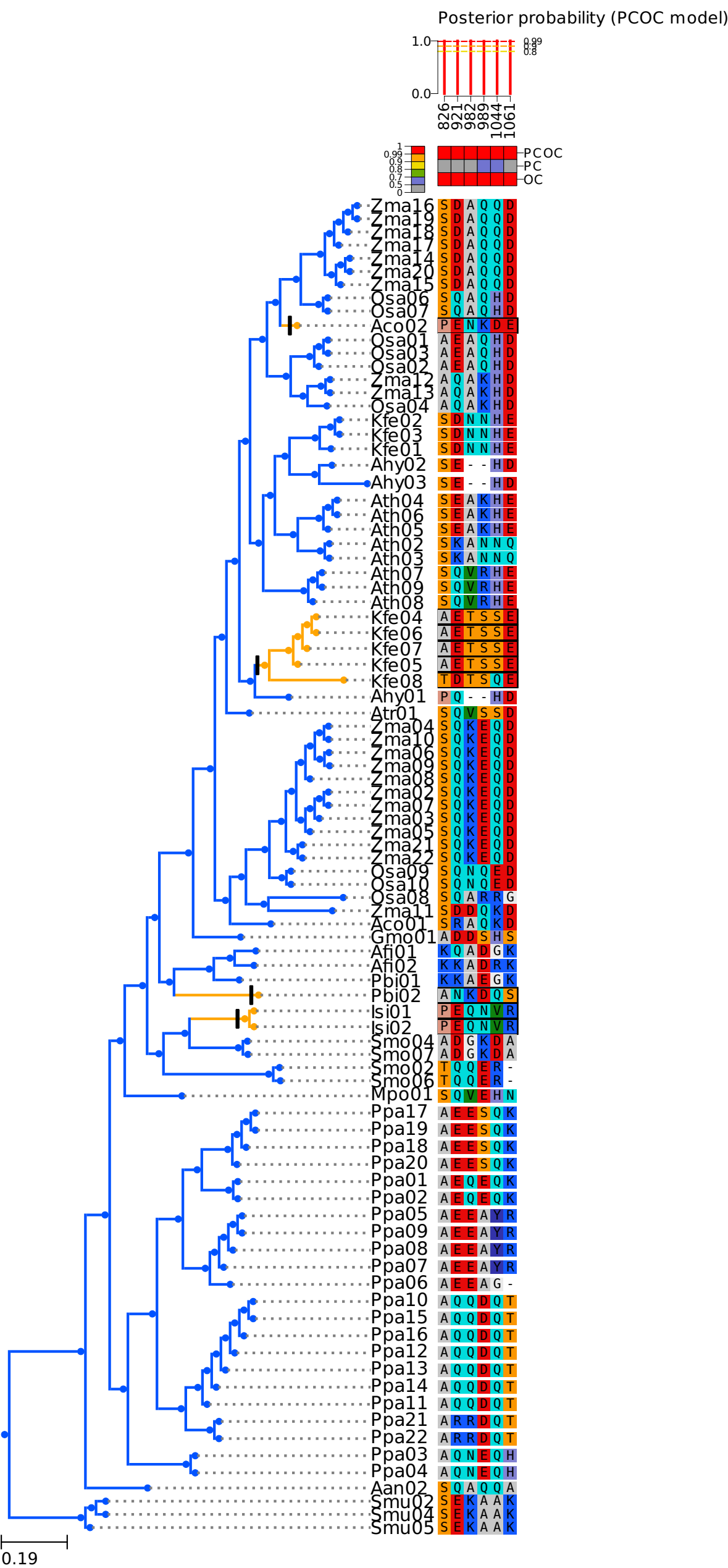

Supplement: Supplemental Information 2 — 1–33: PEPC gene/clade combinations in CAM plants. 34–42: PEPC gene/clade combinations in C4 plants. PCOC: Profile Change with One Change model; PC: Profile Change model; OC: One Change model, all models were in detail explained by Rey et al. (2018). Posterior probabilities (pp) for the PCOC, PC, and OC models are summarized by top box colors, and the amino acid colors correspond to different amino acid equilibrium frequencies (i.e., different profiles) of the Profile Change with One Change model (PCOC model). Aan, Anthoceros angustus; Aco, Ananas comosus; Afi, Azolla filiculoides; Ahy, Amaranthus hypochondriacus; Atr, Amborella trichopoda; Ath, Arabidopsis thaliana; Gmo, Gnetum montanum; Isi, Isoetes sinensis; Kfe, Kalanchoe fedtschenkoi; Mpo, Marchantia polymorpha; Osa, Oryza sativa; Pab, Picea abies; Pbi, Platycerium bifurcatum; Ppa, Physcomitrella patens; Smo, Selaginella moellendorffii; Smu, Spirogloea muscicola; Zma, Zea mays. [file peerj-10-12828-s002.zip › Figure S2/Figure S2-29.pdf]

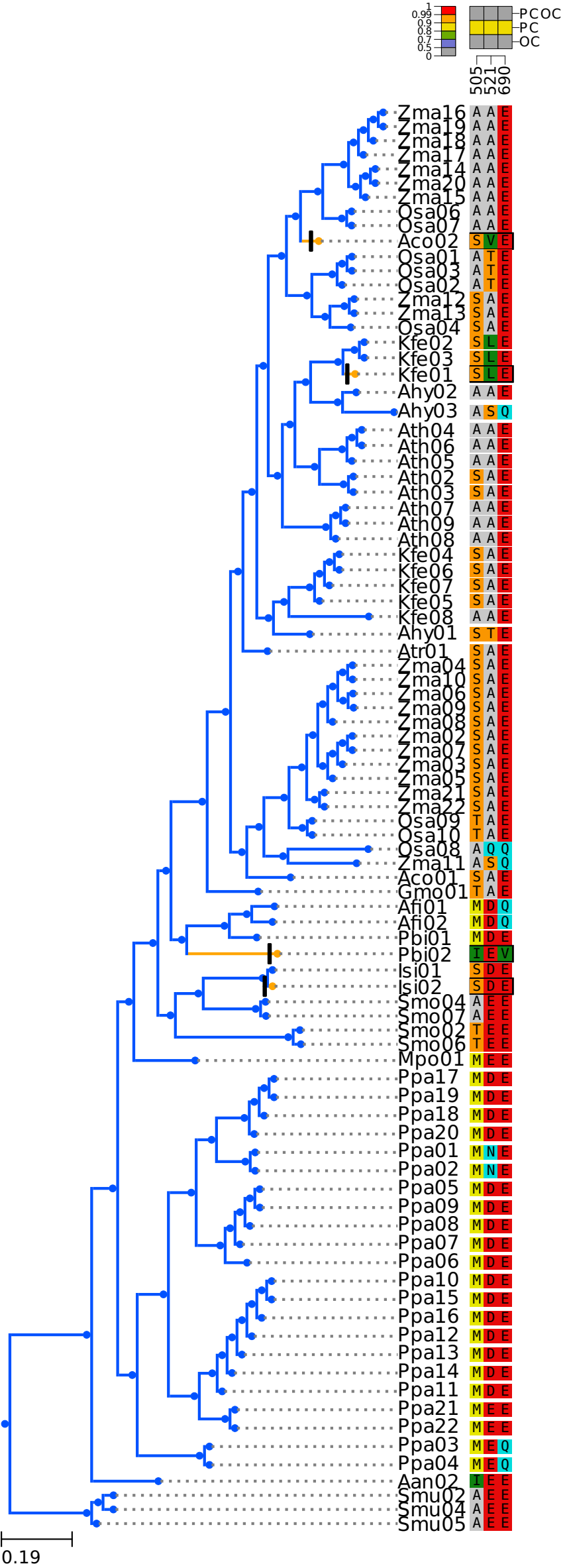

Supplement: Supplemental Information 2 — 1–33: PEPC gene/clade combinations in CAM plants. 34–42: PEPC gene/clade combinations in C4 plants. PCOC: Profile Change with One Change model; PC: Profile Change model; OC: One Change model, all models were in detail explained by Rey et al. (2018). Posterior probabilities (pp) for the PCOC, PC, and OC models are summarized by top box colors, and the amino acid colors correspond to different amino acid equilibrium frequencies (i.e., different profiles) of the Profile Change with One Change model (PCOC model). Aan, Anthoceros angustus; Aco, Ananas comosus; Afi, Azolla filiculoides; Ahy, Amaranthus hypochondriacus; Atr, Amborella trichopoda; Ath, Arabidopsis thaliana; Gmo, Gnetum montanum; Isi, Isoetes sinensis; Kfe, Kalanchoe fedtschenkoi; Mpo, Marchantia polymorpha; Osa, Oryza sativa; Pab, Picea abies; Pbi, Platycerium bifurcatum; Ppa, Physcomitrella patens; Smo, Selaginella moellendorffii; Smu, Spirogloea muscicola; Zma, Zea mays. [file peerj-10-12828-s002.zip › Figure S2/Figure S2-3.pdf]

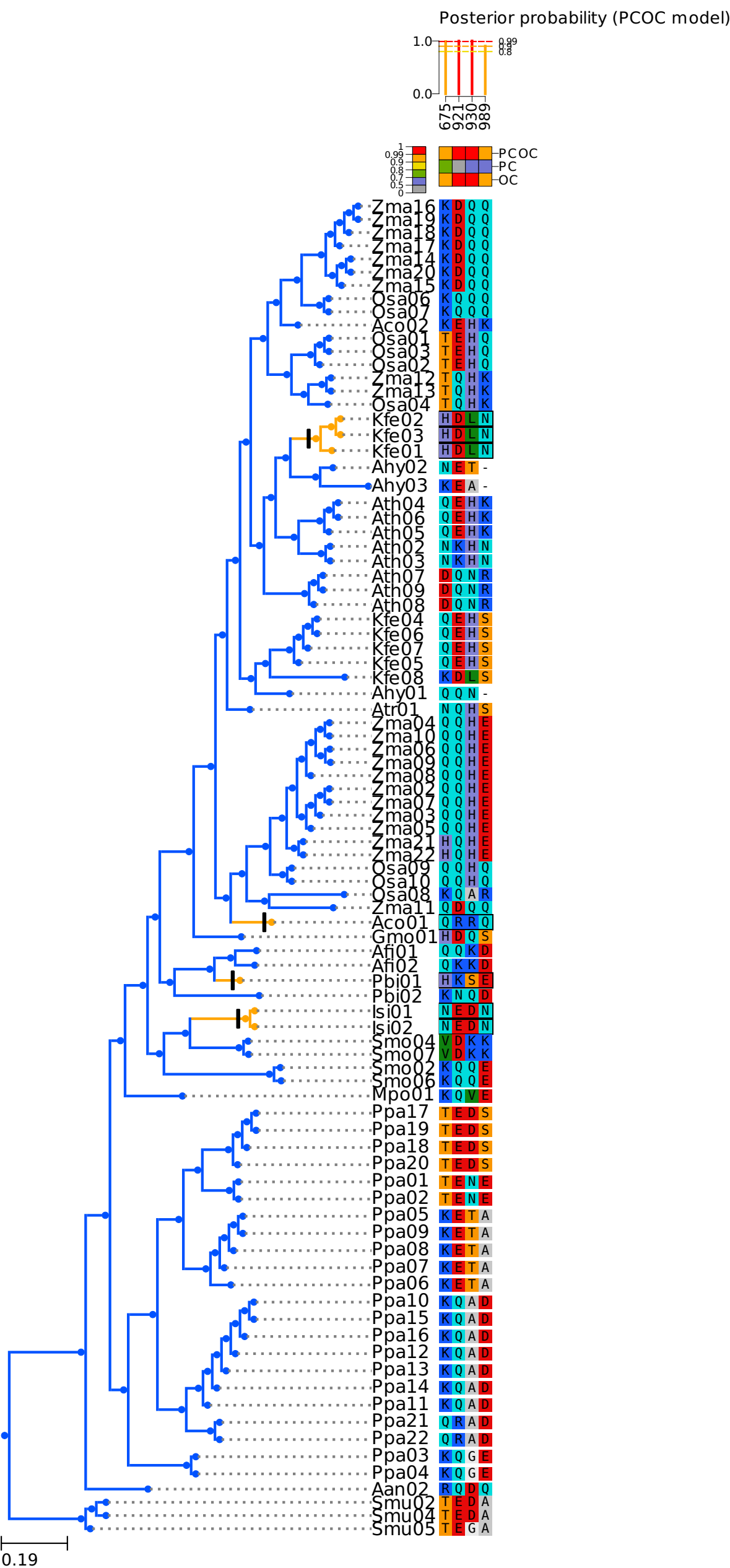

Supplement: Supplemental Information 2 — 1–33: PEPC gene/clade combinations in CAM plants. 34–42: PEPC gene/clade combinations in C4 plants. PCOC: Profile Change with One Change model; PC: Profile Change model; OC: One Change model, all models were in detail explained by Rey et al. (2018). Posterior probabilities (pp) for the PCOC, PC, and OC models are summarized by top box colors, and the amino acid colors correspond to different amino acid equilibrium frequencies (i.e., different profiles) of the Profile Change with One Change model (PCOC model). Aan, Anthoceros angustus; Aco, Ananas comosus; Afi, Azolla filiculoides; Ahy, Amaranthus hypochondriacus; Atr, Amborella trichopoda; Ath, Arabidopsis thaliana; Gmo, Gnetum montanum; Isi, Isoetes sinensis; Kfe, Kalanchoe fedtschenkoi; Mpo, Marchantia polymorpha; Osa, Oryza sativa; Pab, Picea abies; Pbi, Platycerium bifurcatum; Ppa, Physcomitrella patens; Smo, Selaginella moellendorffii; Smu, Spirogloea muscicola; Zma, Zea mays. [file peerj-10-12828-s002.zip › Figure S2/Figure S2-30.pdf]

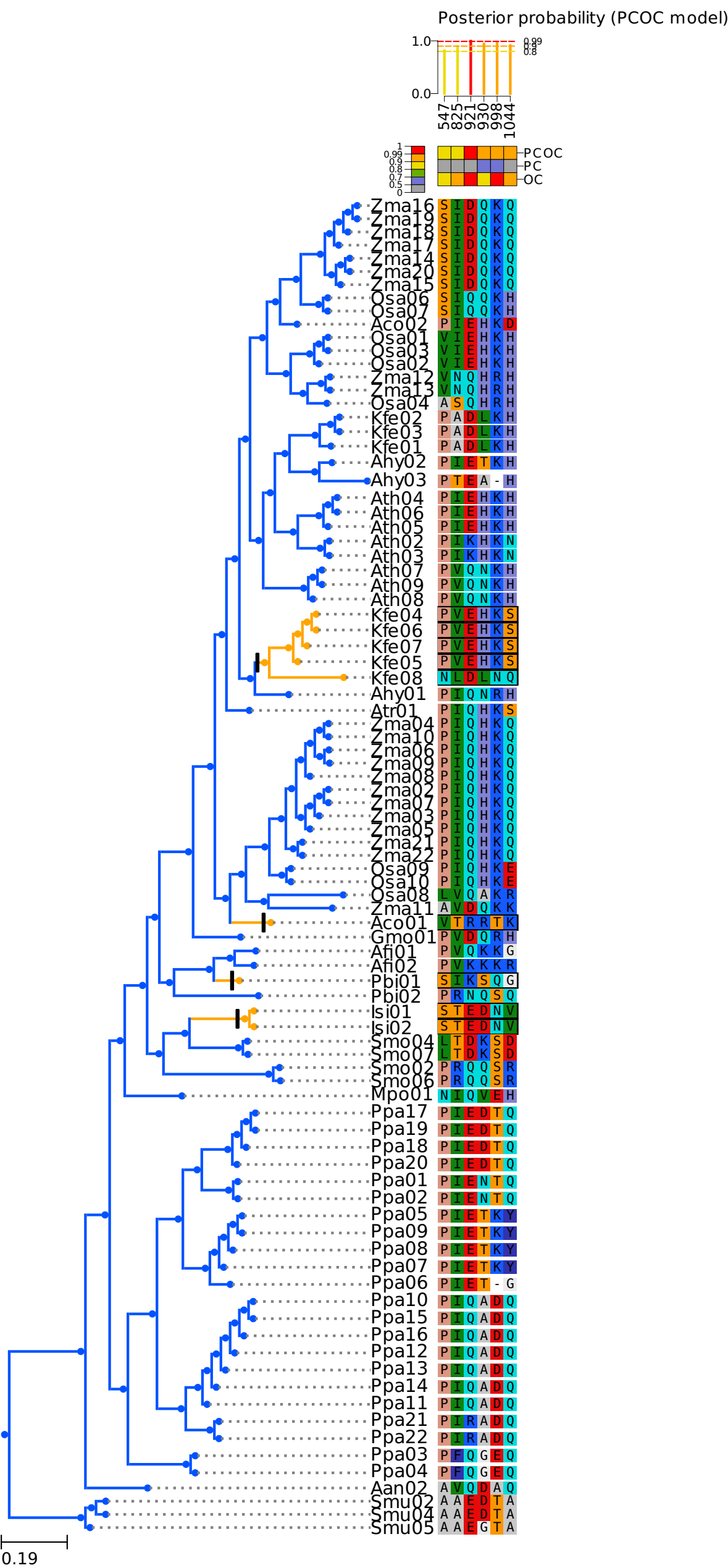

Supplement: Supplemental Information 2 — 1–33: PEPC gene/clade combinations in CAM plants. 34–42: PEPC gene/clade combinations in C4 plants. PCOC: Profile Change with One Change model; PC: Profile Change model; OC: One Change model, all models were in detail explained by Rey et al. (2018). Posterior probabilities (pp) for the PCOC, PC, and OC models are summarized by top box colors, and the amino acid colors correspond to different amino acid equilibrium frequencies (i.e., different profiles) of the Profile Change with One Change model (PCOC model). Aan, Anthoceros angustus; Aco, Ananas comosus; Afi, Azolla filiculoides; Ahy, Amaranthus hypochondriacus; Atr, Amborella trichopoda; Ath, Arabidopsis thaliana; Gmo, Gnetum montanum; Isi, Isoetes sinensis; Kfe, Kalanchoe fedtschenkoi; Mpo, Marchantia polymorpha; Osa, Oryza sativa; Pab, Picea abies; Pbi, Platycerium bifurcatum; Ppa, Physcomitrella patens; Smo, Selaginella moellendorffii; Smu, Spirogloea muscicola; Zma, Zea mays. [file peerj-10-12828-s002.zip › Figure S2/Figure S2-31.pdf]

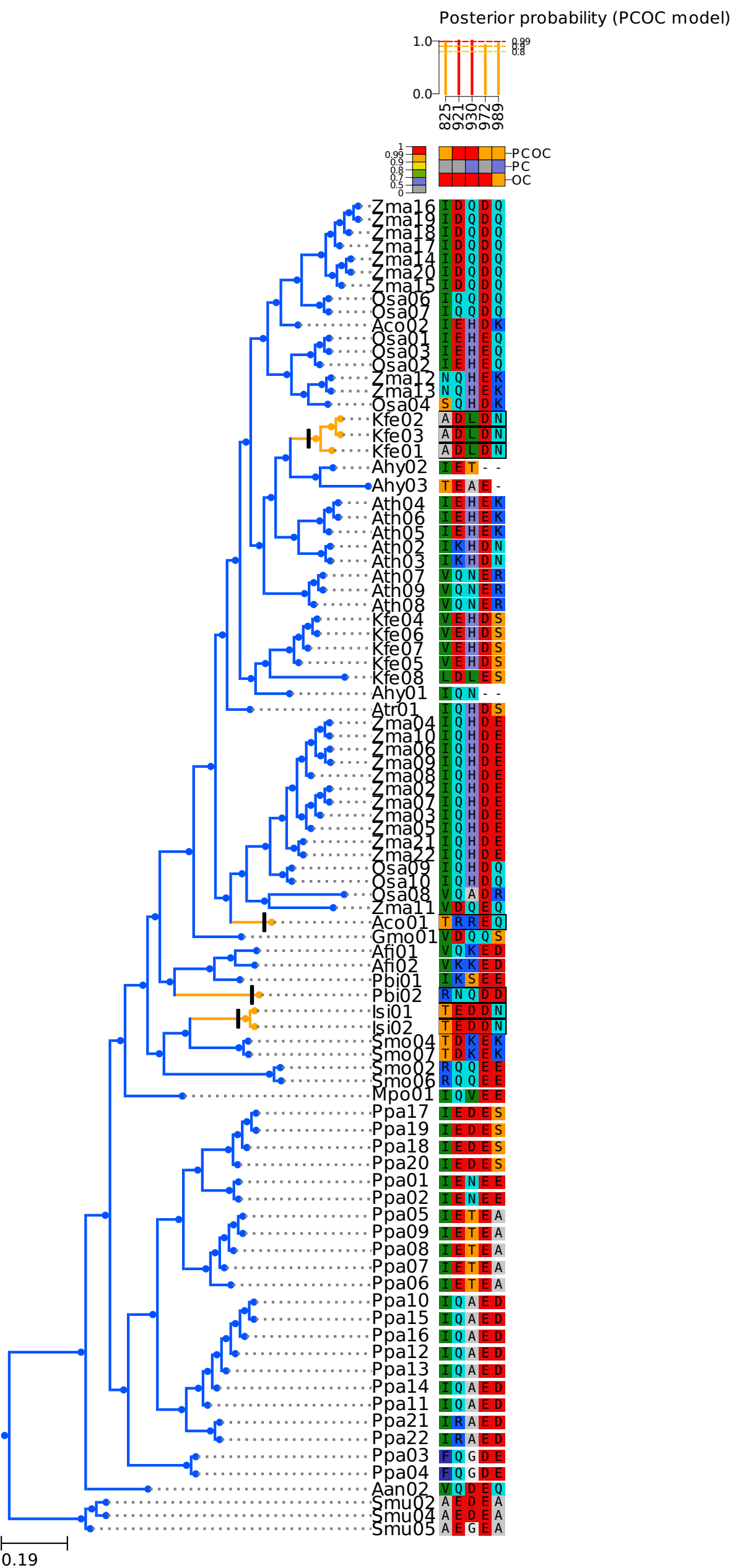

Supplement: Supplemental Information 2 — 1–33: PEPC gene/clade combinations in CAM plants. 34–42: PEPC gene/clade combinations in C4 plants. PCOC: Profile Change with One Change model; PC: Profile Change model; OC: One Change model, all models were in detail explained by Rey et al. (2018). Posterior probabilities (pp) for the PCOC, PC, and OC models are summarized by top box colors, and the amino acid colors correspond to different amino acid equilibrium frequencies (i.e., different profiles) of the Profile Change with One Change model (PCOC model). Aan, Anthoceros angustus; Aco, Ananas comosus; Afi, Azolla filiculoides; Ahy, Amaranthus hypochondriacus; Atr, Amborella trichopoda; Ath, Arabidopsis thaliana; Gmo, Gnetum montanum; Isi, Isoetes sinensis; Kfe, Kalanchoe fedtschenkoi; Mpo, Marchantia polymorpha; Osa, Oryza sativa; Pab, Picea abies; Pbi, Platycerium bifurcatum; Ppa, Physcomitrella patens; Smo, Selaginella moellendorffii; Smu, Spirogloea muscicola; Zma, Zea mays. [file peerj-10-12828-s002.zip › Figure S2/Figure S2-32.pdf]

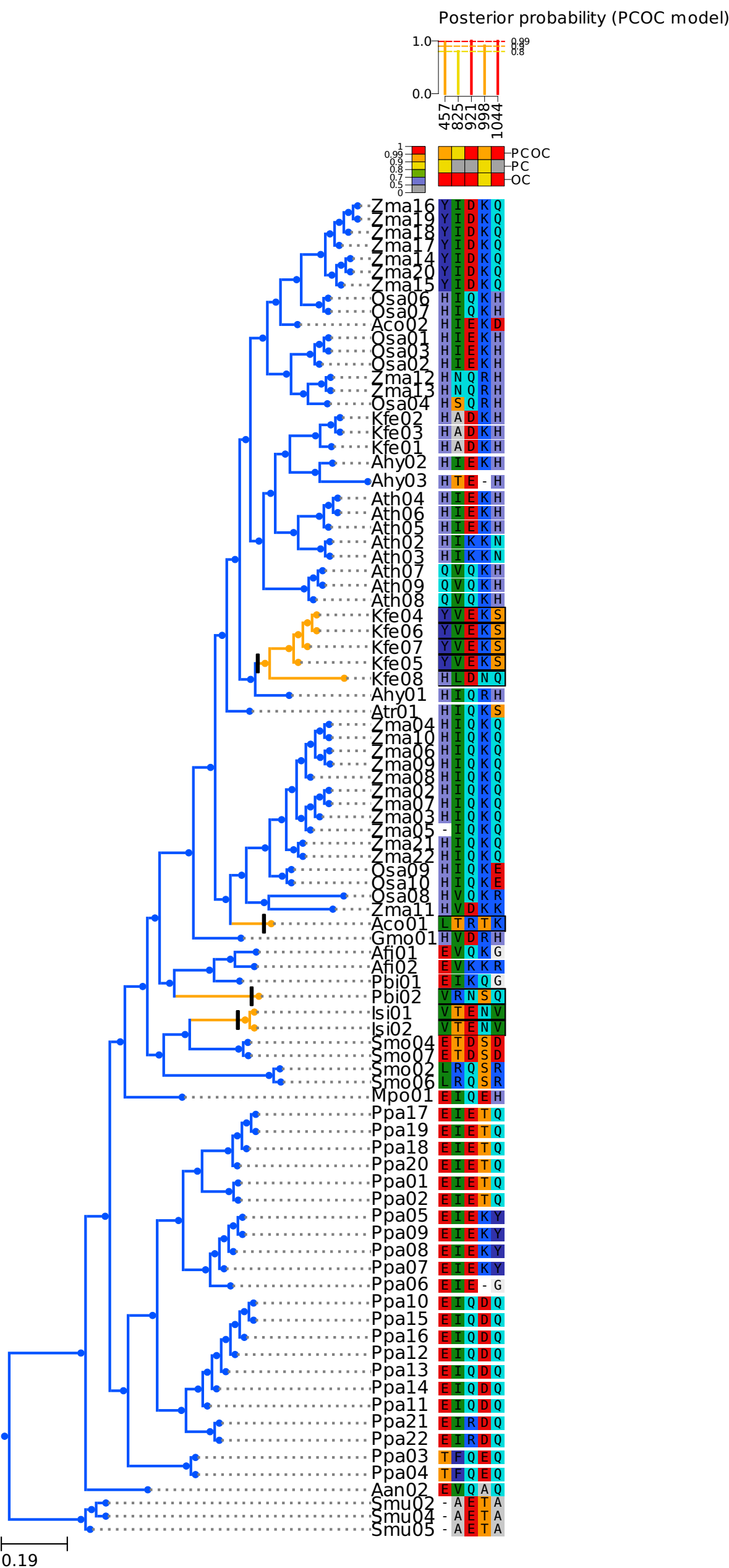

Supplement: Supplemental Information 2 — 1–33: PEPC gene/clade combinations in CAM plants. 34–42: PEPC gene/clade combinations in C4 plants. PCOC: Profile Change with One Change model; PC: Profile Change model; OC: One Change model, all models were in detail explained by Rey et al. (2018). Posterior probabilities (pp) for the PCOC, PC, and OC models are summarized by top box colors, and the amino acid colors correspond to different amino acid equilibrium frequencies (i.e., different profiles) of the Profile Change with One Change model (PCOC model). Aan, Anthoceros angustus; Aco, Ananas comosus; Afi, Azolla filiculoides; Ahy, Amaranthus hypochondriacus; Atr, Amborella trichopoda; Ath, Arabidopsis thaliana; Gmo, Gnetum montanum; Isi, Isoetes sinensis; Kfe, Kalanchoe fedtschenkoi; Mpo, Marchantia polymorpha; Osa, Oryza sativa; Pab, Picea abies; Pbi, Platycerium bifurcatum; Ppa, Physcomitrella patens; Smo, Selaginella moellendorffii; Smu, Spirogloea muscicola; Zma, Zea mays. [file peerj-10-12828-s002.zip › Figure S2/Figure S2-33.pdf]

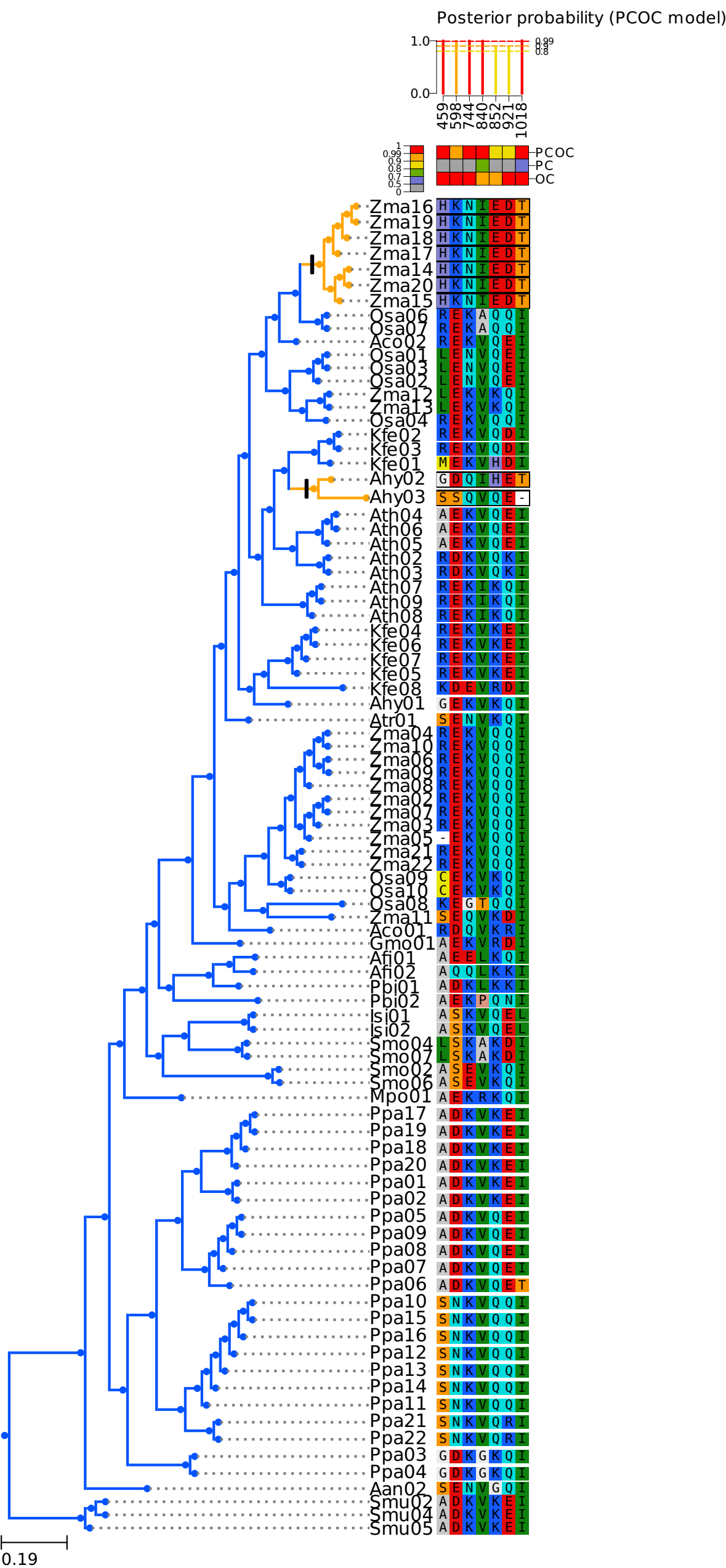

Supplement: Supplemental Information 2 — 1–33: PEPC gene/clade combinations in CAM plants. 34–42: PEPC gene/clade combinations in C4 plants. PCOC: Profile Change with One Change model; PC: Profile Change model; OC: One Change model, all models were in detail explained by Rey et al. (2018). Posterior probabilities (pp) for the PCOC, PC, and OC models are summarized by top box colors, and the amino acid colors correspond to different amino acid equilibrium frequencies (i.e., different profiles) of the Profile Change with One Change model (PCOC model). Aan, Anthoceros angustus; Aco, Ananas comosus; Afi, Azolla filiculoides; Ahy, Amaranthus hypochondriacus; Atr, Amborella trichopoda; Ath, Arabidopsis thaliana; Gmo, Gnetum montanum; Isi, Isoetes sinensis; Kfe, Kalanchoe fedtschenkoi; Mpo, Marchantia polymorpha; Osa, Oryza sativa; Pab, Picea abies; Pbi, Platycerium bifurcatum; Ppa, Physcomitrella patens; Smo, Selaginella moellendorffii; Smu, Spirogloea muscicola; Zma, Zea mays. [file peerj-10-12828-s002.zip › Figure S2/Figure S2-35.pdf]

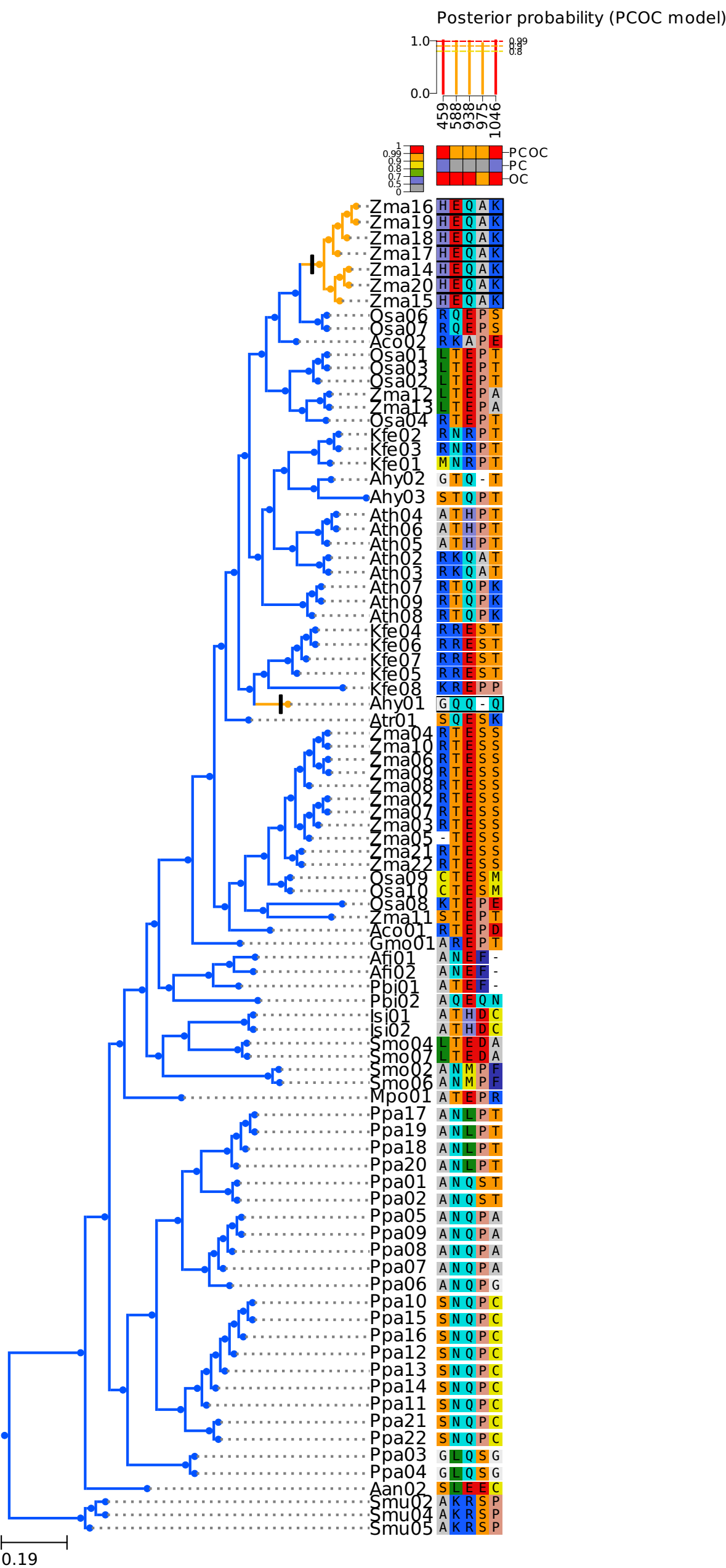

Supplement: Supplemental Information 2 — 1–33: PEPC gene/clade combinations in CAM plants. 34–42: PEPC gene/clade combinations in C4 plants. PCOC: Profile Change with One Change model; PC: Profile Change model; OC: One Change model, all models were in detail explained by Rey et al. (2018). Posterior probabilities (pp) for the PCOC, PC, and OC models are summarized by top box colors, and the amino acid colors correspond to different amino acid equilibrium frequencies (i.e., different profiles) of the Profile Change with One Change model (PCOC model). Aan, Anthoceros angustus; Aco, Ananas comosus; Afi, Azolla filiculoides; Ahy, Amaranthus hypochondriacus; Atr, Amborella trichopoda; Ath, Arabidopsis thaliana; Gmo, Gnetum montanum; Isi, Isoetes sinensis; Kfe, Kalanchoe fedtschenkoi; Mpo, Marchantia polymorpha; Osa, Oryza sativa; Pab, Picea abies; Pbi, Platycerium bifurcatum; Ppa, Physcomitrella patens; Smo, Selaginella moellendorffii; Smu, Spirogloea muscicola; Zma, Zea mays. [file peerj-10-12828-s002.zip › Figure S2/Figure S2-36.pdf]

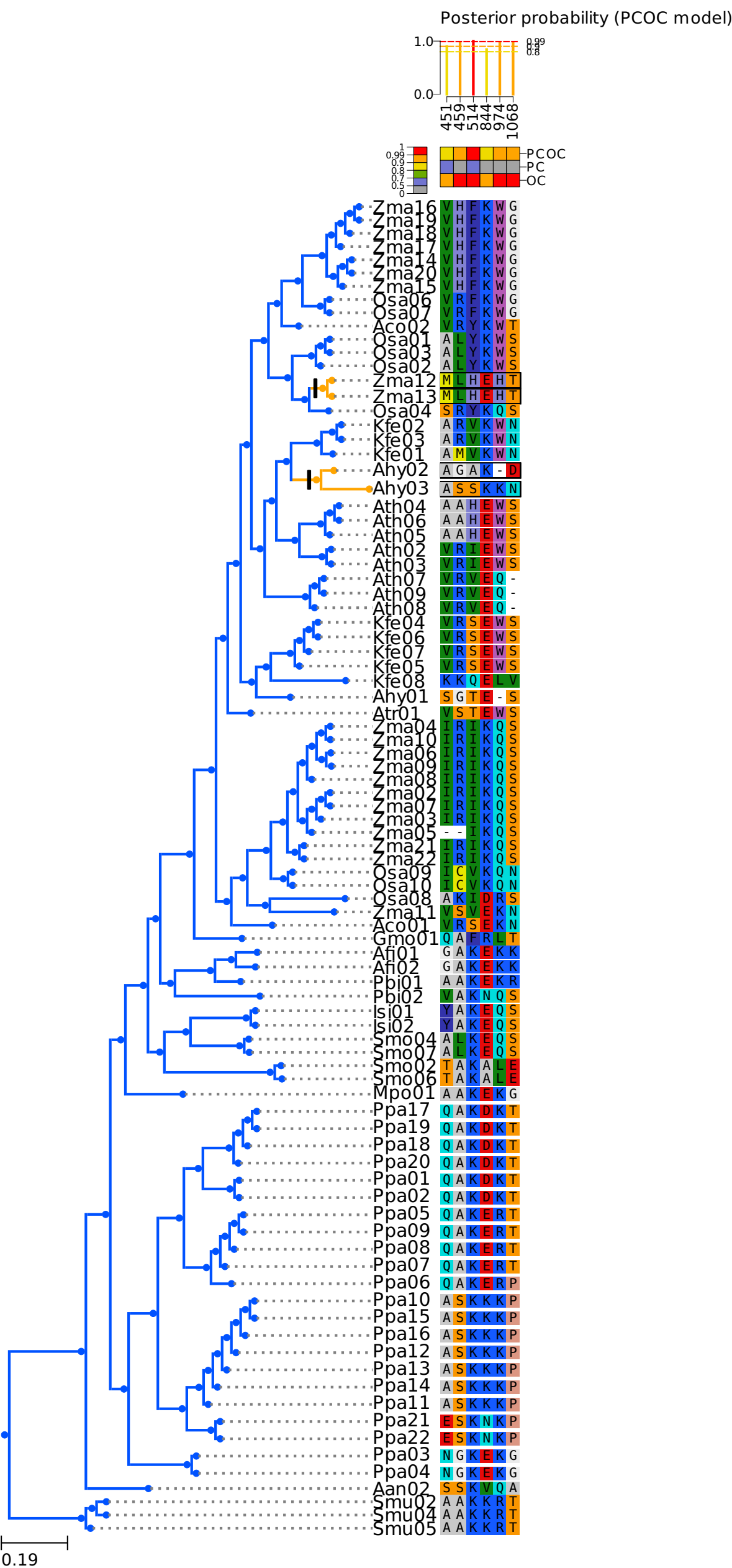

Supplement: Supplemental Information 2 — 1–33: PEPC gene/clade combinations in CAM plants. 34–42: PEPC gene/clade combinations in C4 plants. PCOC: Profile Change with One Change model; PC: Profile Change model; OC: One Change model, all models were in detail explained by Rey et al. (2018). Posterior probabilities (pp) for the PCOC, PC, and OC models are summarized by top box colors, and the amino acid colors correspond to different amino acid equilibrium frequencies (i.e., different profiles) of the Profile Change with One Change model (PCOC model). Aan, Anthoceros angustus; Aco, Ananas comosus; Afi, Azolla filiculoides; Ahy, Amaranthus hypochondriacus; Atr, Amborella trichopoda; Ath, Arabidopsis thaliana; Gmo, Gnetum montanum; Isi, Isoetes sinensis; Kfe, Kalanchoe fedtschenkoi; Mpo, Marchantia polymorpha; Osa, Oryza sativa; Pab, Picea abies; Pbi, Platycerium bifurcatum; Ppa, Physcomitrella patens; Smo, Selaginella moellendorffii; Smu, Spirogloea muscicola; Zma, Zea mays. [file peerj-10-12828-s002.zip › Figure S2/Figure S2-37.pdf]

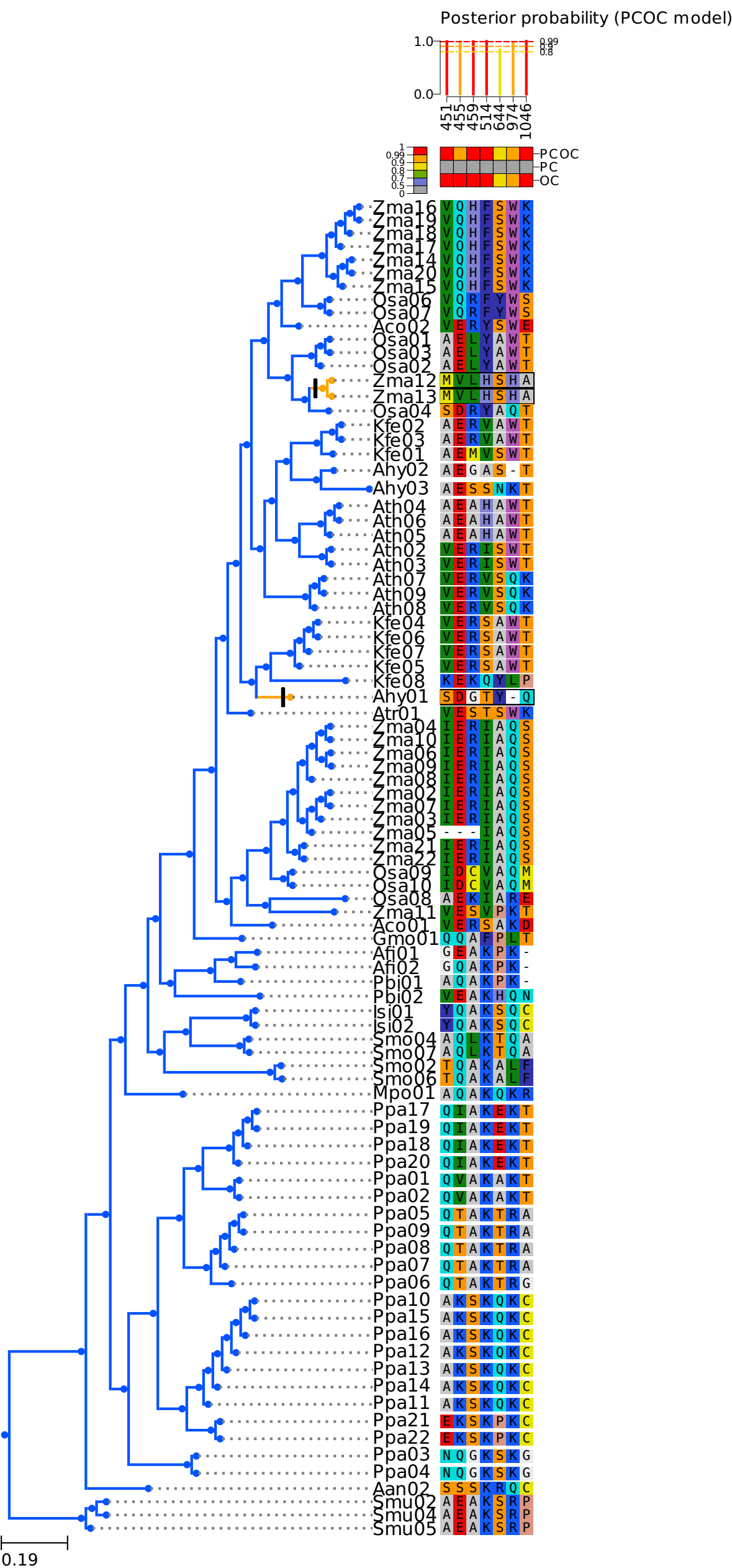

Supplement: Supplemental Information 2 — 1–33: PEPC gene/clade combinations in CAM plants. 34–42: PEPC gene/clade combinations in C4 plants. PCOC: Profile Change with One Change model; PC: Profile Change model; OC: One Change model, all models were in detail explained by Rey et al. (2018). Posterior probabilities (pp) for the PCOC, PC, and OC models are summarized by top box colors, and the amino acid colors correspond to different amino acid equilibrium frequencies (i.e., different profiles) of the Profile Change with One Change model (PCOC model). Aan, Anthoceros angustus; Aco, Ananas comosus; Afi, Azolla filiculoides; Ahy, Amaranthus hypochondriacus; Atr, Amborella trichopoda; Ath, Arabidopsis thaliana; Gmo, Gnetum montanum; Isi, Isoetes sinensis; Kfe, Kalanchoe fedtschenkoi; Mpo, Marchantia polymorpha; Osa, Oryza sativa; Pab, Picea abies; Pbi, Platycerium bifurcatum; Ppa, Physcomitrella patens; Smo, Selaginella moellendorffii; Smu, Spirogloea muscicola; Zma, Zea mays. [file peerj-10-12828-s002.zip › Figure S2/Figure S2-38.pdf]

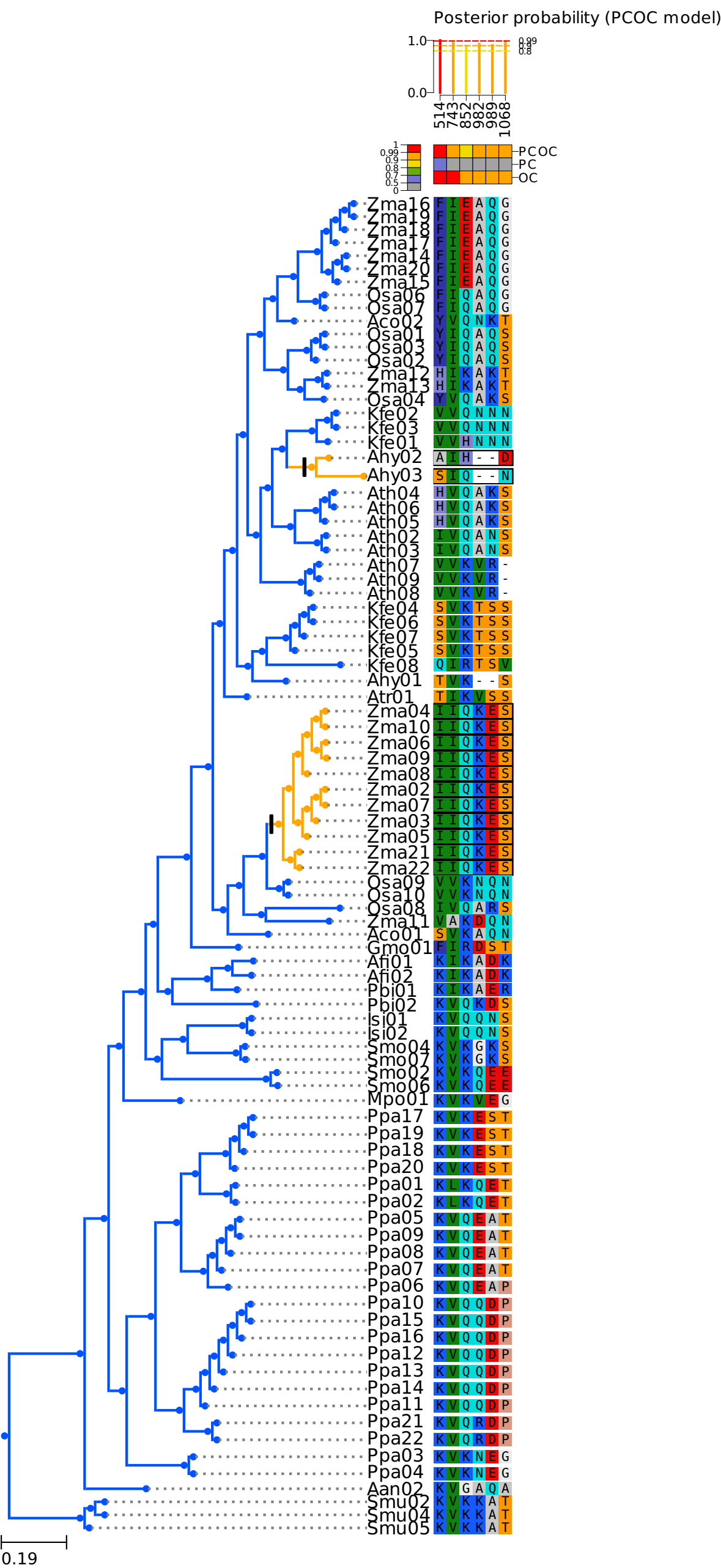

Supplement: Supplemental Information 2 — 1–33: PEPC gene/clade combinations in CAM plants. 34–42: PEPC gene/clade combinations in C4 plants. PCOC: Profile Change with One Change model; PC: Profile Change model; OC: One Change model, all models were in detail explained by Rey et al. (2018). Posterior probabilities (pp) for the PCOC, PC, and OC models are summarized by top box colors, and the amino acid colors correspond to different amino acid equilibrium frequencies (i.e., different profiles) of the Profile Change with One Change model (PCOC model). Aan, Anthoceros angustus; Aco, Ananas comosus; Afi, Azolla filiculoides; Ahy, Amaranthus hypochondriacus; Atr, Amborella trichopoda; Ath, Arabidopsis thaliana; Gmo, Gnetum montanum; Isi, Isoetes sinensis; Kfe, Kalanchoe fedtschenkoi; Mpo, Marchantia polymorpha; Osa, Oryza sativa; Pab, Picea abies; Pbi, Platycerium bifurcatum; Ppa, Physcomitrella patens; Smo, Selaginella moellendorffii; Smu, Spirogloea muscicola; Zma, Zea mays. [file peerj-10-12828-s002.zip › Figure S2/Figure S2-39.pdf]

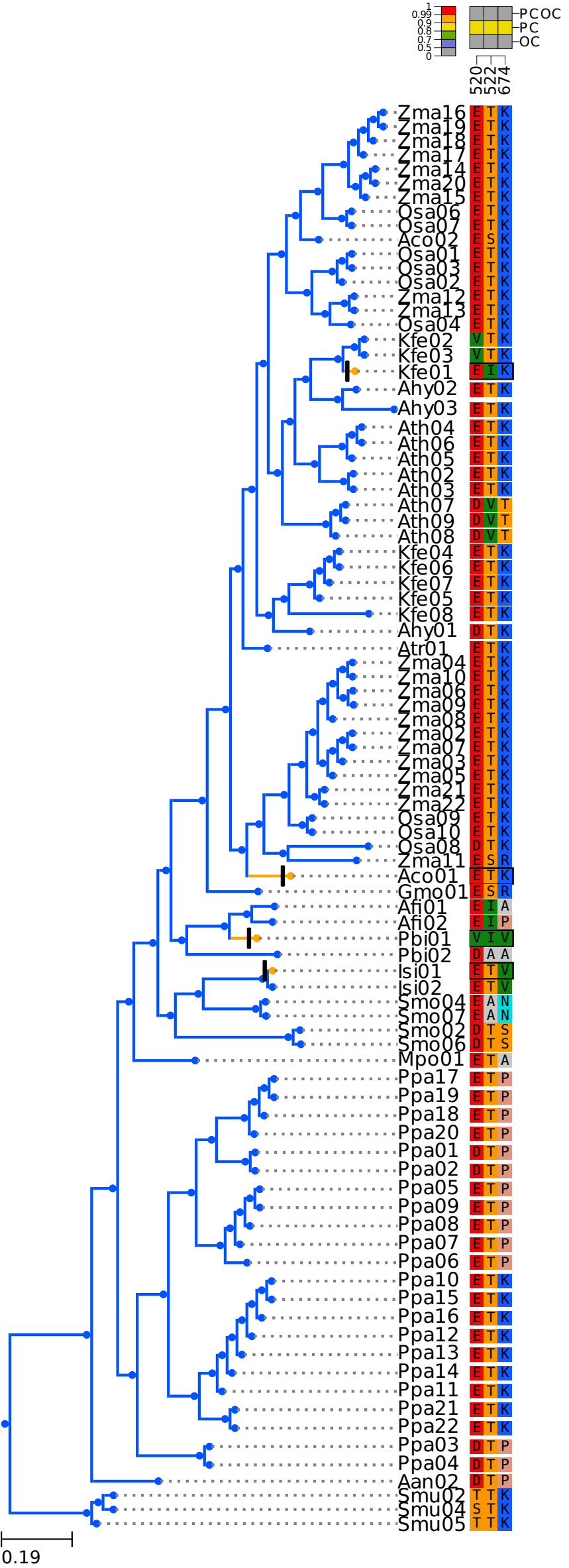

Supplement: Supplemental Information 2 — 1–33: PEPC gene/clade combinations in CAM plants. 34–42: PEPC gene/clade combinations in C4 plants. PCOC: Profile Change with One Change model; PC: Profile Change model; OC: One Change model, all models were in detail explained by Rey et al. (2018). Posterior probabilities (pp) for the PCOC, PC, and OC models are summarized by top box colors, and the amino acid colors correspond to different amino acid equilibrium frequencies (i.e., different profiles) of the Profile Change with One Change model (PCOC model). Aan, Anthoceros angustus; Aco, Ananas comosus; Afi, Azolla filiculoides; Ahy, Amaranthus hypochondriacus; Atr, Amborella trichopoda; Ath, Arabidopsis thaliana; Gmo, Gnetum montanum; Isi, Isoetes sinensis; Kfe, Kalanchoe fedtschenkoi; Mpo, Marchantia polymorpha; Osa, Oryza sativa; Pab, Picea abies; Pbi, Platycerium bifurcatum; Ppa, Physcomitrella patens; Smo, Selaginella moellendorffii; Smu, Spirogloea muscicola; Zma, Zea mays. [file peerj-10-12828-s002.zip › Figure S2/Figure S2-4.pdf]

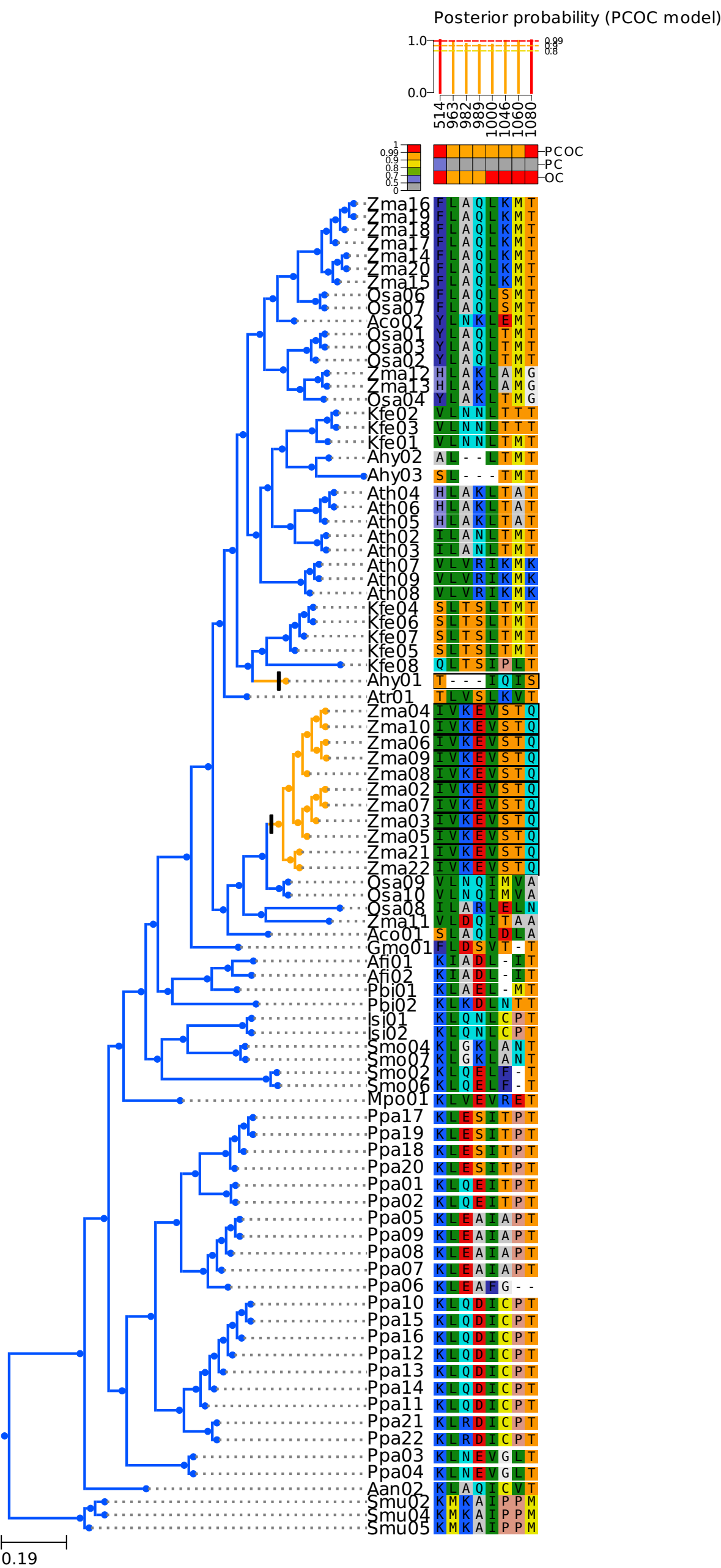

Supplement: Supplemental Information 2 — 1–33: PEPC gene/clade combinations in CAM plants. 34–42: PEPC gene/clade combinations in C4 plants. PCOC: Profile Change with One Change model; PC: Profile Change model; OC: One Change model, all models were in detail explained by Rey et al. (2018). Posterior probabilities (pp) for the PCOC, PC, and OC models are summarized by top box colors, and the amino acid colors correspond to different amino acid equilibrium frequencies (i.e., different profiles) of the Profile Change with One Change model (PCOC model). Aan, Anthoceros angustus; Aco, Ananas comosus; Afi, Azolla filiculoides; Ahy, Amaranthus hypochondriacus; Atr, Amborella trichopoda; Ath, Arabidopsis thaliana; Gmo, Gnetum montanum; Isi, Isoetes sinensis; Kfe, Kalanchoe fedtschenkoi; Mpo, Marchantia polymorpha; Osa, Oryza sativa; Pab, Picea abies; Pbi, Platycerium bifurcatum; Ppa, Physcomitrella patens; Smo, Selaginella moellendorffii; Smu, Spirogloea muscicola; Zma, Zea mays. [file peerj-10-12828-s002.zip › Figure S2/Figure S2-40.pdf]

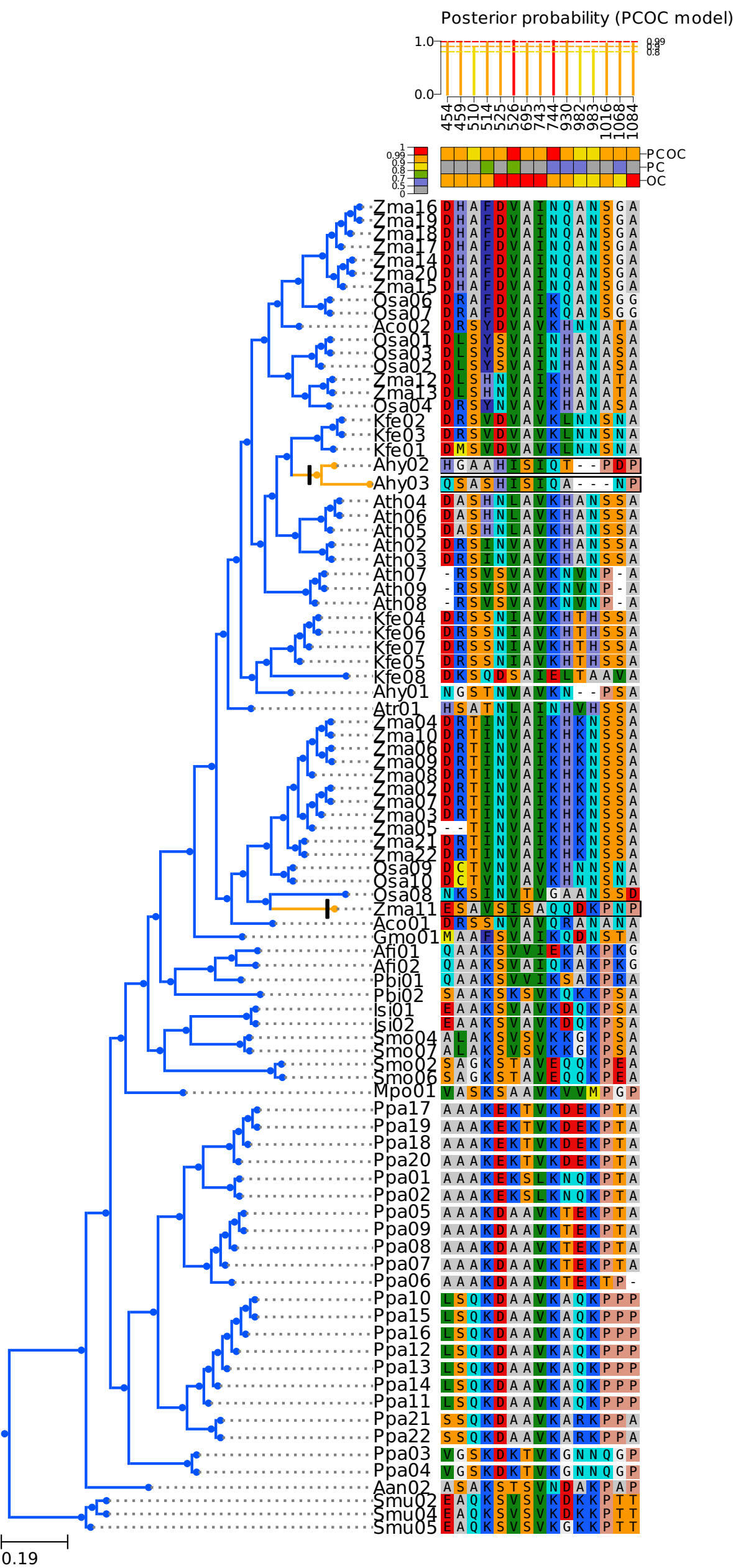

Supplement: Supplemental Information 2 — 1–33: PEPC gene/clade combinations in CAM plants. 34–42: PEPC gene/clade combinations in C4 plants. PCOC: Profile Change with One Change model; PC: Profile Change model; OC: One Change model, all models were in detail explained by Rey et al. (2018). Posterior probabilities (pp) for the PCOC, PC, and OC models are summarized by top box colors, and the amino acid colors correspond to different amino acid equilibrium frequencies (i.e., different profiles) of the Profile Change with One Change model (PCOC model). Aan, Anthoceros angustus; Aco, Ananas comosus; Afi, Azolla filiculoides; Ahy, Amaranthus hypochondriacus; Atr, Amborella trichopoda; Ath, Arabidopsis thaliana; Gmo, Gnetum montanum; Isi, Isoetes sinensis; Kfe, Kalanchoe fedtschenkoi; Mpo, Marchantia polymorpha; Osa, Oryza sativa; Pab, Picea abies; Pbi, Platycerium bifurcatum; Ppa, Physcomitrella patens; Smo, Selaginella moellendorffii; Smu, Spirogloea muscicola; Zma, Zea mays. [file peerj-10-12828-s002.zip › Figure S2/Figure S2-41.pdf]

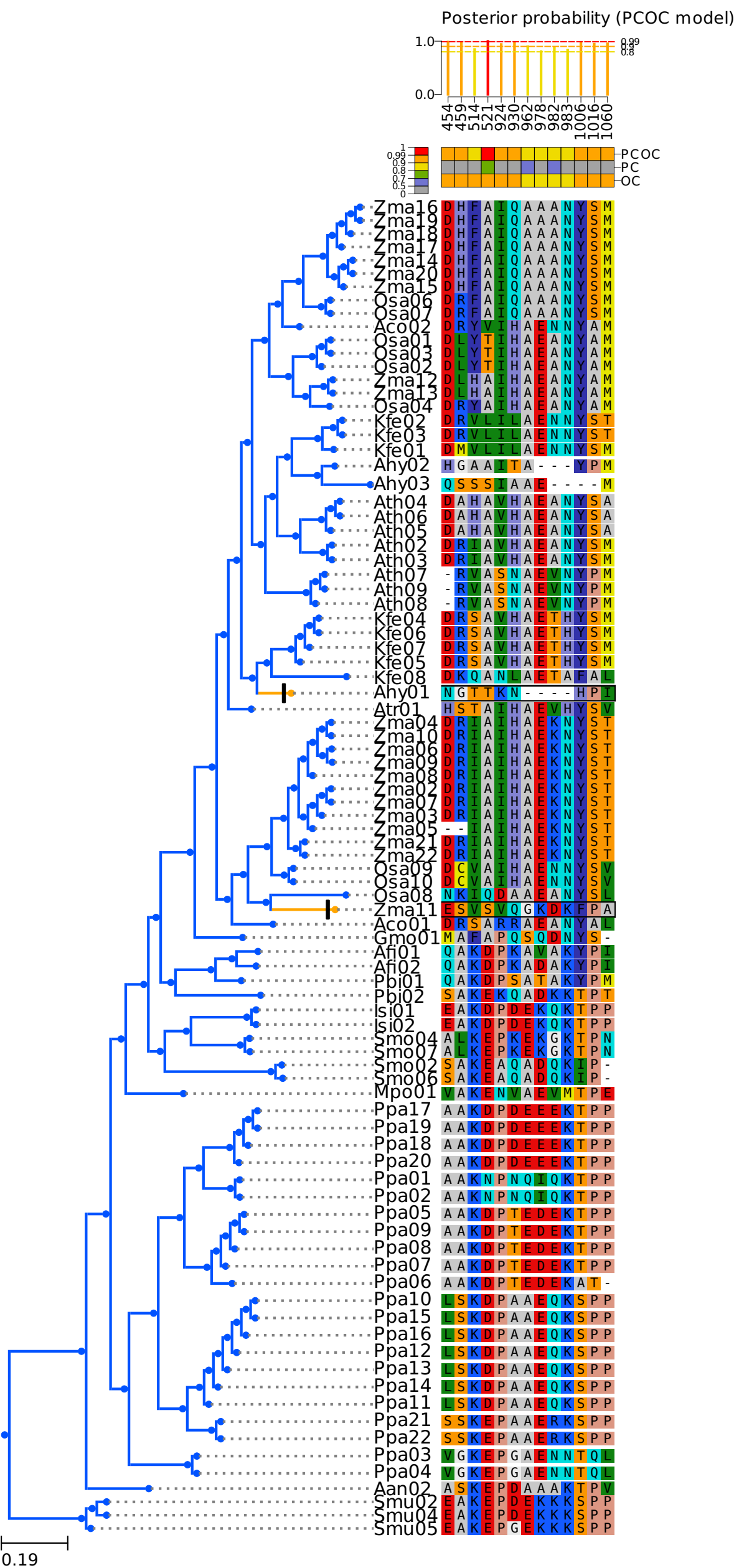

Supplement: Supplemental Information 2 — 1–33: PEPC gene/clade combinations in CAM plants. 34–42: PEPC gene/clade combinations in C4 plants. PCOC: Profile Change with One Change model; PC: Profile Change model; OC: One Change model, all models were in detail explained by Rey et al. (2018). Posterior probabilities (pp) for the PCOC, PC, and OC models are summarized by top box colors, and the amino acid colors correspond to different amino acid equilibrium frequencies (i.e., different profiles) of the Profile Change with One Change model (PCOC model). Aan, Anthoceros angustus; Aco, Ananas comosus; Afi, Azolla filiculoides; Ahy, Amaranthus hypochondriacus; Atr, Amborella trichopoda; Ath, Arabidopsis thaliana; Gmo, Gnetum montanum; Isi, Isoetes sinensis; Kfe, Kalanchoe fedtschenkoi; Mpo, Marchantia polymorpha; Osa, Oryza sativa; Pab, Picea abies; Pbi, Platycerium bifurcatum; Ppa, Physcomitrella patens; Smo, Selaginella moellendorffii; Smu, Spirogloea muscicola; Zma, Zea mays. [file peerj-10-12828-s002.zip › Figure S2/Figure S2-42.pdf]

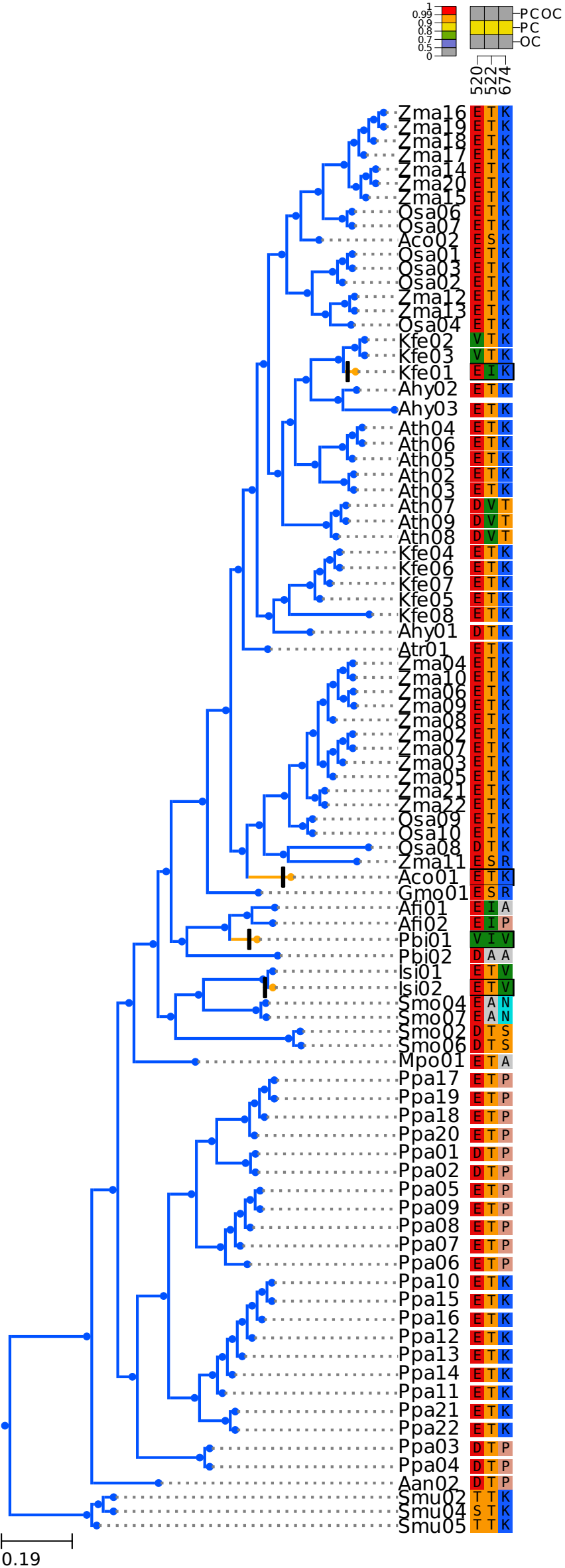

Supplement: Supplemental Information 2 — 1–33: PEPC gene/clade combinations in CAM plants. 34–42: PEPC gene/clade combinations in C4 plants. PCOC: Profile Change with One Change model; PC: Profile Change model; OC: One Change model, all models were in detail explained by Rey et al. (2018). Posterior probabilities (pp) for the PCOC, PC, and OC models are summarized by top box colors, and the amino acid colors correspond to different amino acid equilibrium frequencies (i.e., different profiles) of the Profile Change with One Change model (PCOC model). Aan, Anthoceros angustus; Aco, Ananas comosus; Afi, Azolla filiculoides; Ahy, Amaranthus hypochondriacus; Atr, Amborella trichopoda; Ath, Arabidopsis thaliana; Gmo, Gnetum montanum; Isi, Isoetes sinensis; Kfe, Kalanchoe fedtschenkoi; Mpo, Marchantia polymorpha; Osa, Oryza sativa; Pab, Picea abies; Pbi, Platycerium bifurcatum; Ppa, Physcomitrella patens; Smo, Selaginella moellendorffii; Smu, Spirogloea muscicola; Zma, Zea mays. [file peerj-10-12828-s002.zip › Figure S2/Figure S2-5.pdf]

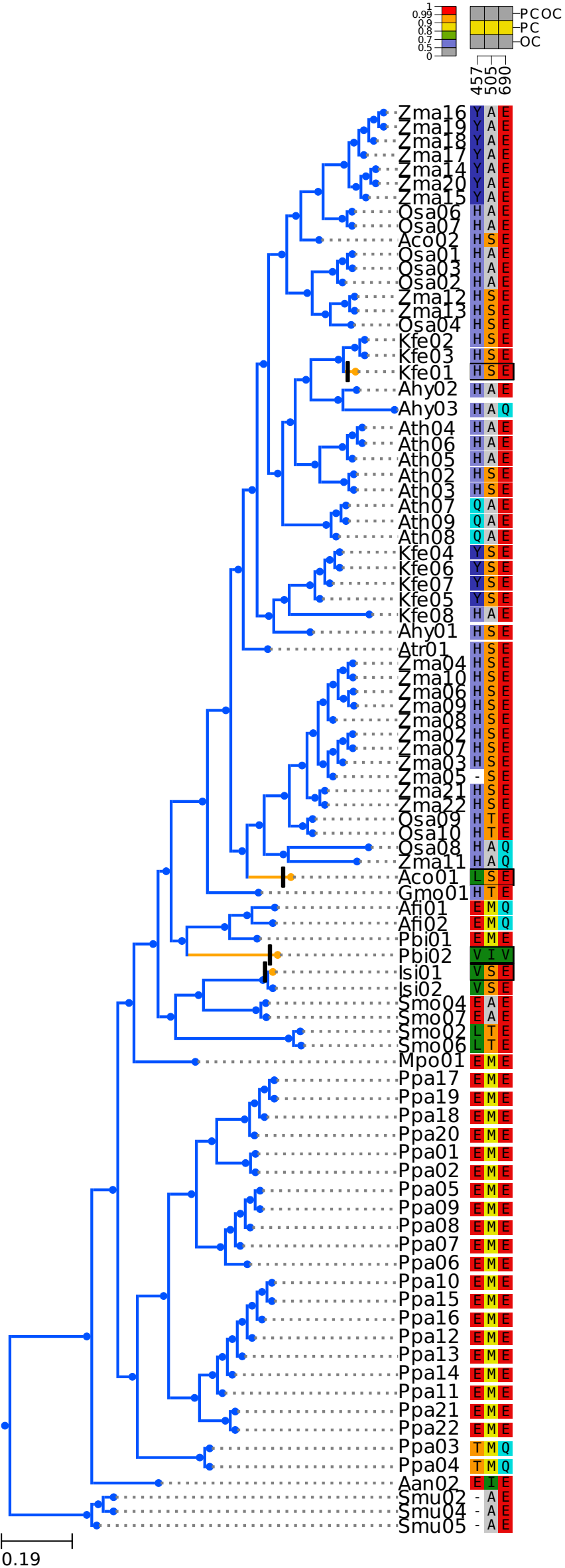

Supplement: Supplemental Information 2 — 1–33: PEPC gene/clade combinations in CAM plants. 34–42: PEPC gene/clade combinations in C4 plants. PCOC: Profile Change with One Change model; PC: Profile Change model; OC: One Change model, all models were in detail explained by Rey et al. (2018). Posterior probabilities (pp) for the PCOC, PC, and OC models are summarized by top box colors, and the amino acid colors correspond to different amino acid equilibrium frequencies (i.e., different profiles) of the Profile Change with One Change model (PCOC model). Aan, Anthoceros angustus; Aco, Ananas comosus; Afi, Azolla filiculoides; Ahy, Amaranthus hypochondriacus; Atr, Amborella trichopoda; Ath, Arabidopsis thaliana; Gmo, Gnetum montanum; Isi, Isoetes sinensis; Kfe, Kalanchoe fedtschenkoi; Mpo, Marchantia polymorpha; Osa, Oryza sativa; Pab, Picea abies; Pbi, Platycerium bifurcatum; Ppa, Physcomitrella patens; Smo, Selaginella moellendorffii; Smu, Spirogloea muscicola; Zma, Zea mays. [file peerj-10-12828-s002.zip › Figure S2/Figure S2-6.pdf]

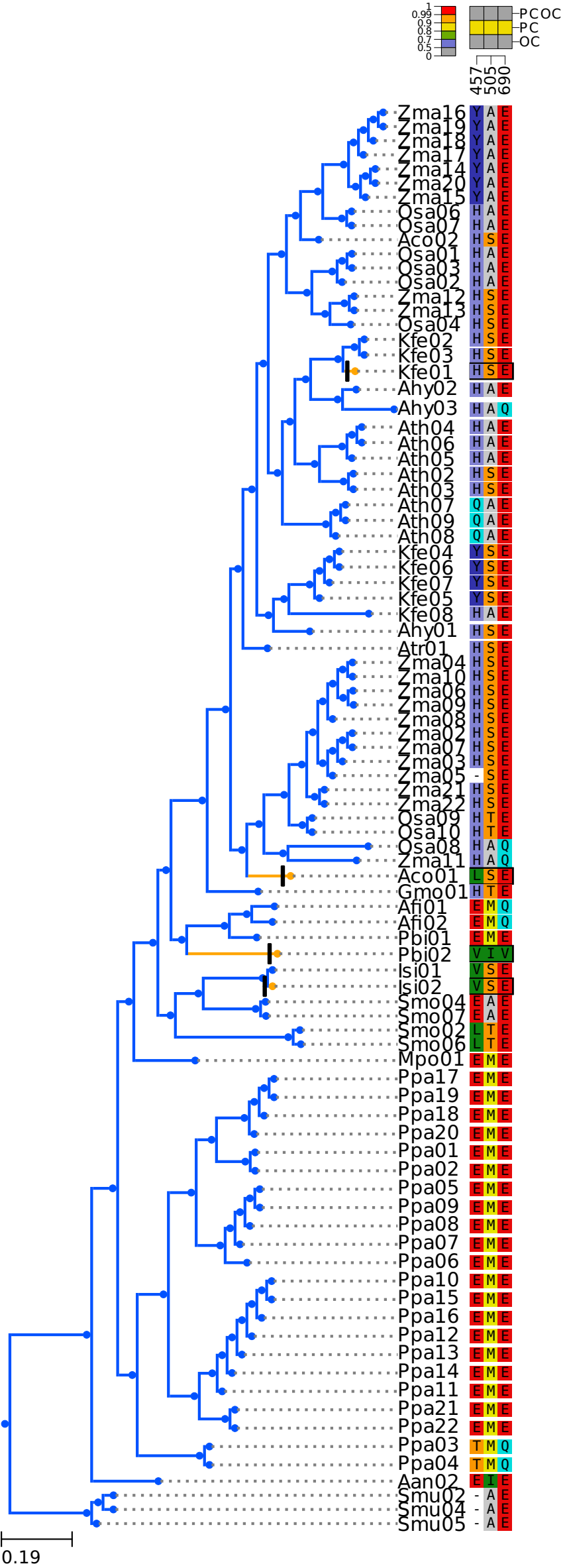

Supplement: Supplemental Information 2 — 1–33: PEPC gene/clade combinations in CAM plants. 34–42: PEPC gene/clade combinations in C4 plants. PCOC: Profile Change with One Change model; PC: Profile Change model; OC: One Change model, all models were in detail explained by Rey et al. (2018). Posterior probabilities (pp) for the PCOC, PC, and OC models are summarized by top box colors, and the amino acid colors correspond to different amino acid equilibrium frequencies (i.e., different profiles) of the Profile Change with One Change model (PCOC model). Aan, Anthoceros angustus; Aco, Ananas comosus; Afi, Azolla filiculoides; Ahy, Amaranthus hypochondriacus; Atr, Amborella trichopoda; Ath, Arabidopsis thaliana; Gmo, Gnetum montanum; Isi, Isoetes sinensis; Kfe, Kalanchoe fedtschenkoi; Mpo, Marchantia polymorpha; Osa, Oryza sativa; Pab, Picea abies; Pbi, Platycerium bifurcatum; Ppa, Physcomitrella patens; Smo, Selaginella moellendorffii; Smu, Spirogloea muscicola; Zma, Zea mays. [file peerj-10-12828-s002.zip › Figure S2/Figure S2-7.pdf]

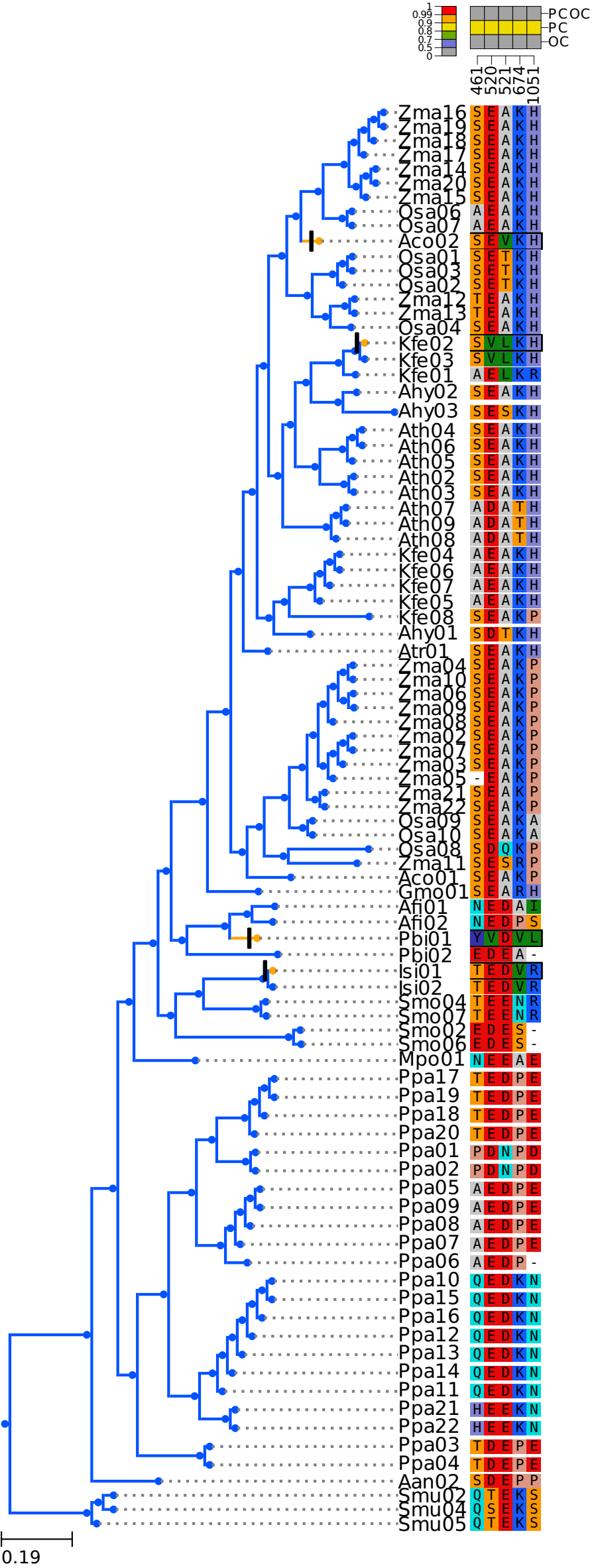

Supplement: Supplemental Information 2 — 1–33: PEPC gene/clade combinations in CAM plants. 34–42: PEPC gene/clade combinations in C4 plants. PCOC: Profile Change with One Change model; PC: Profile Change model; OC: One Change model, all models were in detail explained by Rey et al. (2018). Posterior probabilities (pp) for the PCOC, PC, and OC models are summarized by top box colors, and the amino acid colors correspond to different amino acid equilibrium frequencies (i.e., different profiles) of the Profile Change with One Change model (PCOC model). Aan, Anthoceros angustus; Aco, Ananas comosus; Afi, Azolla filiculoides; Ahy, Amaranthus hypochondriacus; Atr, Amborella trichopoda; Ath, Arabidopsis thaliana; Gmo, Gnetum montanum; Isi, Isoetes sinensis; Kfe, Kalanchoe fedtschenkoi; Mpo, Marchantia polymorpha; Osa, Oryza sativa; Pab, Picea abies; Pbi, Platycerium bifurcatum; Ppa, Physcomitrella patens; Smo, Selaginella moellendorffii; Smu, Spirogloea muscicola; Zma, Zea mays. [file peerj-10-12828-s002.zip › Figure S2/Figure S2-8.pdf]

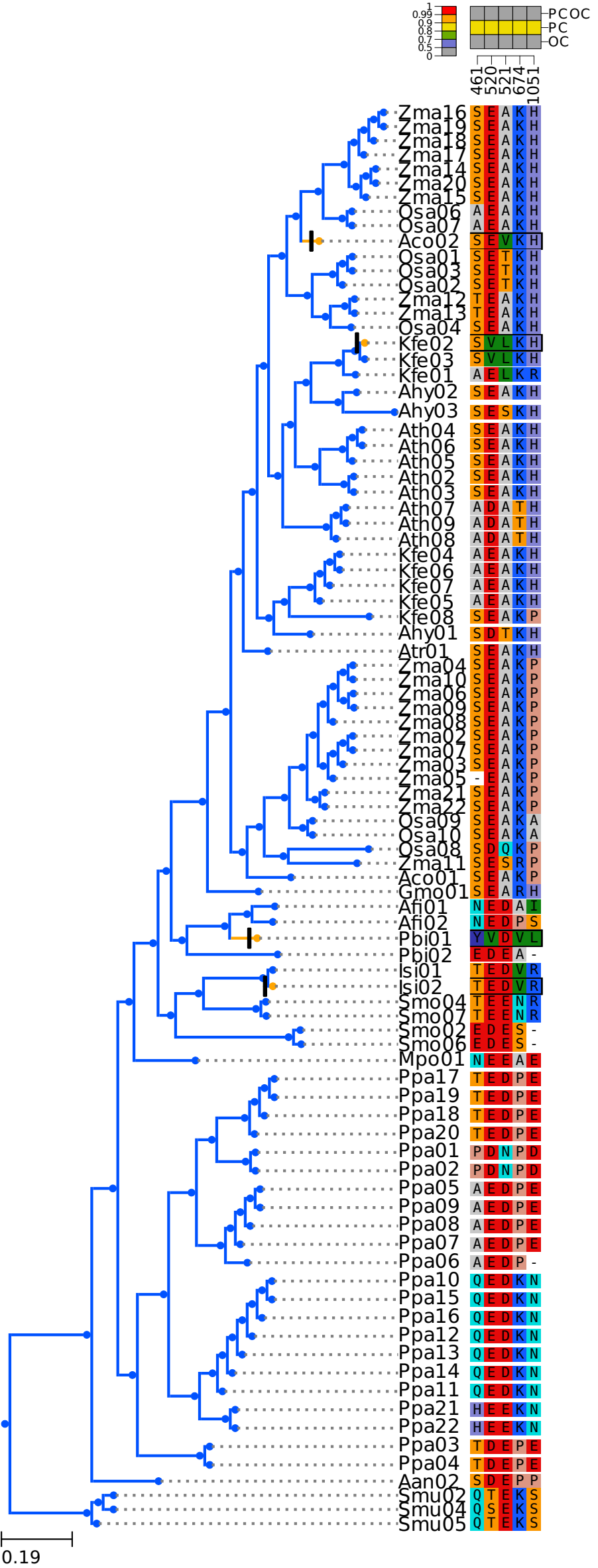

Supplement: Supplemental Information 2 — 1–33: PEPC gene/clade combinations in CAM plants. 34–42: PEPC gene/clade combinations in C4 plants. PCOC: Profile Change with One Change model; PC: Profile Change model; OC: One Change model, all models were in detail explained by Rey et al. (2018). Posterior probabilities (pp) for the PCOC, PC, and OC models are summarized by top box colors, and the amino acid colors correspond to different amino acid equilibrium frequencies (i.e., different profiles) of the Profile Change with One Change model (PCOC model). Aan, Anthoceros angustus; Aco, Ananas comosus; Afi, Azolla filiculoides; Ahy, Amaranthus hypochondriacus; Atr, Amborella trichopoda; Ath, Arabidopsis thaliana; Gmo, Gnetum montanum; Isi, Isoetes sinensis; Kfe, Kalanchoe fedtschenkoi; Mpo, Marchantia polymorpha; Osa, Oryza sativa; Pab, Picea abies; Pbi, Platycerium bifurcatum; Ppa, Physcomitrella patens; Smo, Selaginella moellendorffii; Smu, Spirogloea muscicola; Zma, Zea mays. [file peerj-10-12828-s002.zip › Figure S2/Figure S2-9.pdf]

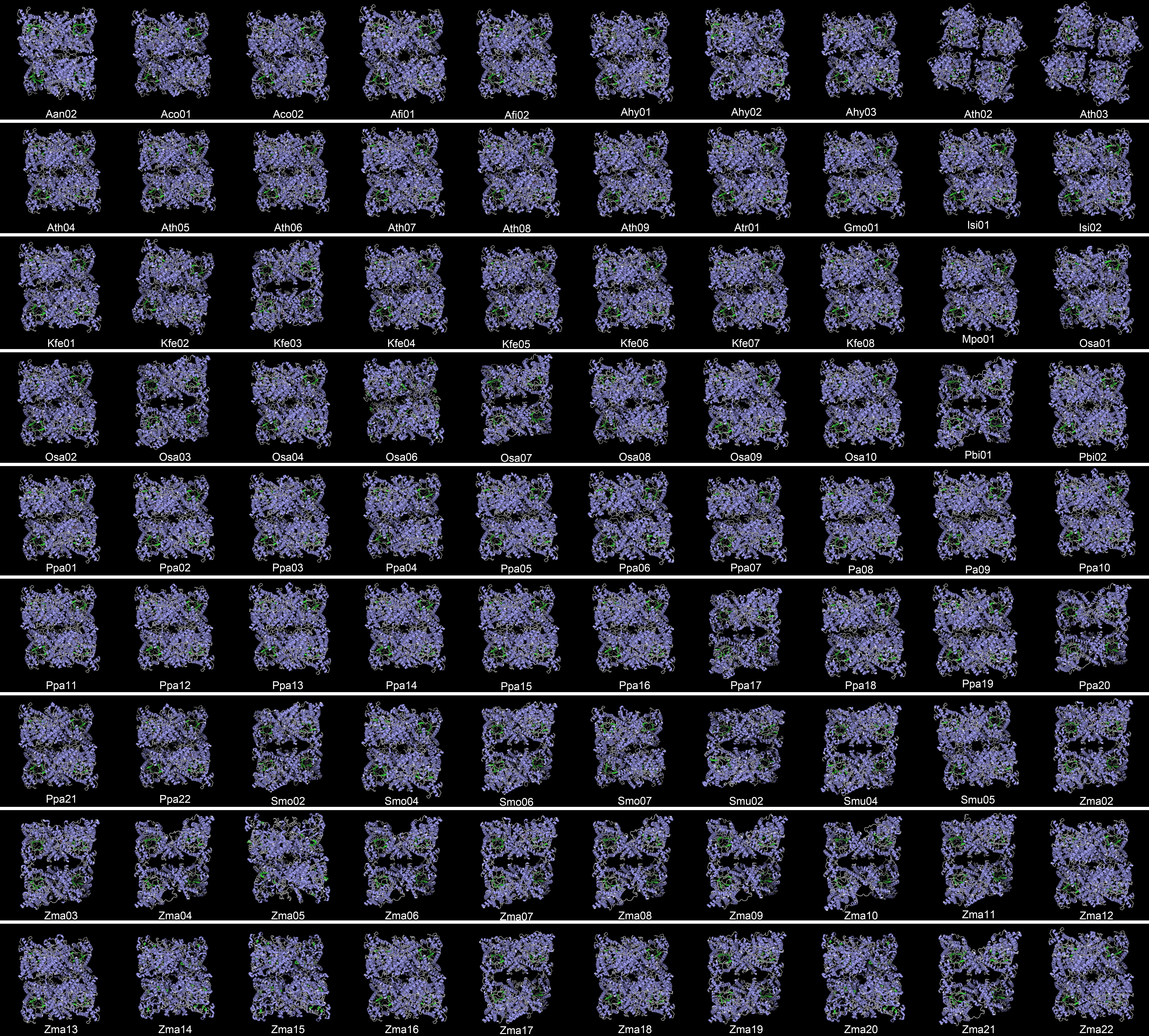

Supplement: Supplemental Information 4 [file peerj-10-12828-s004.jpg]
