# Supplementary material for: Convergent molecular evolution of phosphoenolpyruvate carboxylase gene family in C4 and crassulacean acid metabolism plants
Source: PeerJ. 2022 Jan 20;10:e12828. doi: 10.7717/peerj.12828 (PMC8784020; doi:10.7717/peerj.12828)
Supplement: Supplemental Information 2 — 1–33: PEPC gene/clade combinations in CAM plants. 34–42: PEPC gene/clade combinations in C4 plants. PCOC: Profile Change with One Change model; PC: Profile Change model; OC: One Change model, all models were in detail explained by Rey et al. (2018). Posterior probabilities (pp) for the PCOC, PC, and OC models are summarized by top box colors, and the amino acid colors correspond to different amino acid equilibrium frequencies (i.e., different profiles) of the Profile Change with One Change model (PCOC model). Aan, Anthoceros angustus; Aco, Ananas comosus; Afi, Azolla filiculoides; Ahy, Amaranthus hypochondriacus; Atr, Amborella trichopoda; Ath, Arabidopsis thaliana; Gmo, Gnetum montanum; Isi, Isoetes sinensis; Kfe, Kalanchoe fedtschenkoi; Mpo, Marchantia polymorpha; Osa, Oryza sativa; Pab, Picea abies; Pbi, Platycerium bifurcatum; Ppa, Physcomitrella patens; Smo, Selaginella moellendorffii; Smu, Spirogloea muscicola; Zma, Zea mays. [file Figure S2-34.pdf › Figure S2/Figure S2-34.pdf]

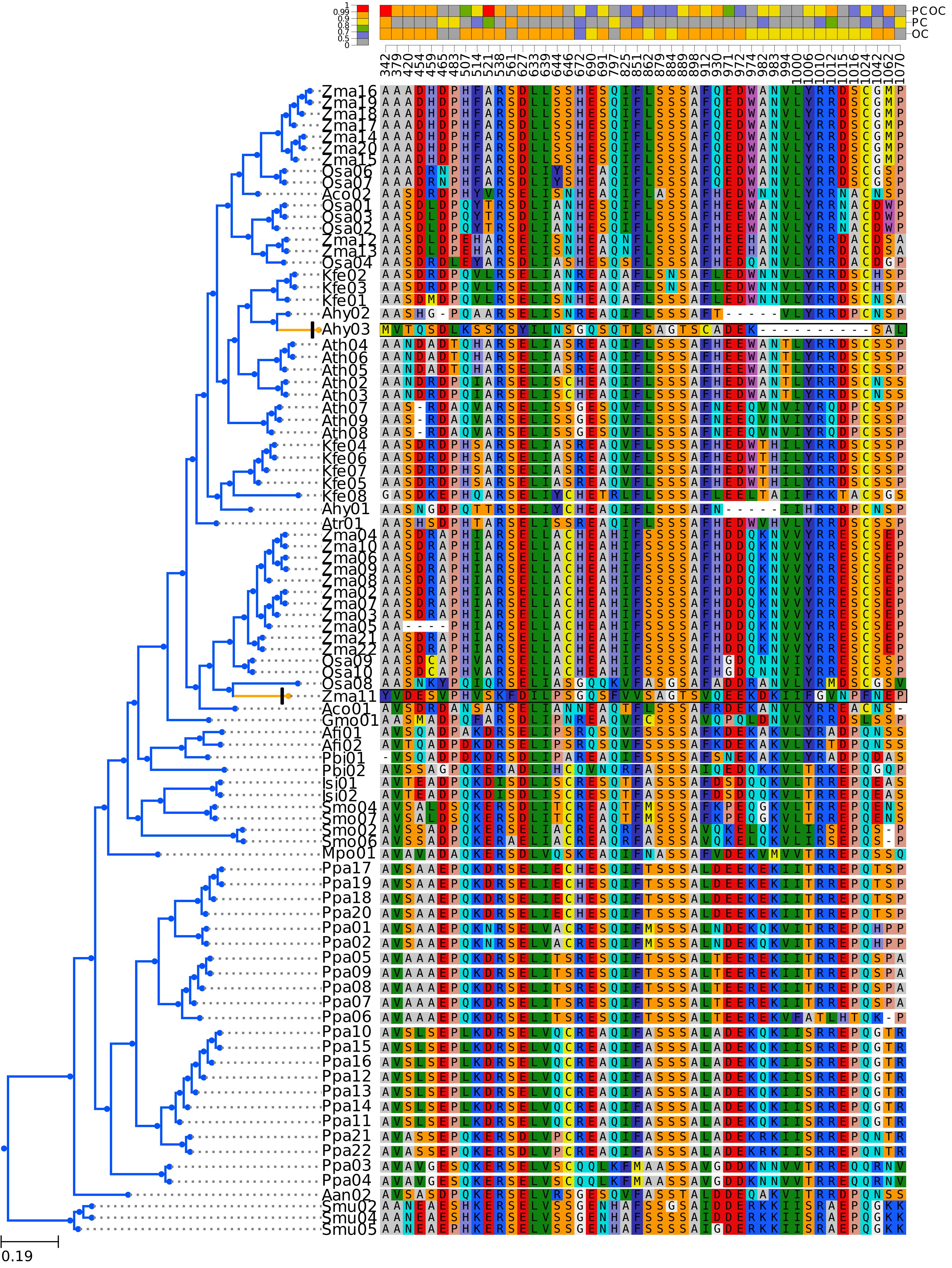
1
0.99
0.9
0.8
0.7
0.5
0
PCOC
PC
OC
342 379 420 454 459 465 483 507 514 521 538 561 627 633 639 644 646 672 690 691 797 825 851 862 879 884 889 898 912 930 971 974 982 983 994 1000 1006 1010 1012 1015 1016 1024 1042 1062 1070
Zma16
Zma19
Zma18
Zma17
Zma14
Zma20
Zma15
Osa06
Osa07
Aco02
Osa01
Osa03
Osa02
Zma12
Zma13
Osa04
Kfe02
Kfe03
Kfe01
Ahy02
Ahy03
Ath04
Ath06
Ath05
Ath02
Ath03
Ath07
Ath09
Ath08
Kfe04
Kfe06
Kfe07
Kfe05
Kfe08
Ahy01
Atr01
Zma04
Zma10
Zma06
Zma09
Zma08
Zma02
Zma07
Zma03
Zma05
Zma21
Zma22
Osa09
Osa10
Osa08
Zma11
Aco01
Gmo01
Afi01
Afi02
Pbi01
Pbi02
Isi01
Isi02
Smo04
Smo07
Smo02
Smo06
Mpo01
Ppa17
Ppa19
Ppa18
Ppa20
Ppa01
Ppa02
Ppa05
Ppa09
Ppa08
Ppa07
Ppa06
Ppa10
Ppa15
Ppa16
Ppa12
Ppa13
Ppa14
Ppa11
Ppa21
Ppa22
Ppa03
Ppa04
Aan02
Smu02
Smu04
Smu05
0.19
